# Integration of the Biot–Gassmann Fluid Substitution Method and Machine Learning-Based Velocity–Stress Relationship for Estimating In Situ Stresses

**DOI:** 10.1021/acsomega.5c09669

**Published:** 2026-01-28

**Authors:** Ayyaz Mustafa, Guanyi Lu, Andrew P. Bunger

**Affiliations:** † Department of Civil and Environmental Engineering, 6614 University of Pittsburgh, Pittsburgh 15261, Pennsylvania, United States; ‡ Department of Civil and Environmental Engineering, University of Pittsburgh, Pittsburgh 15261, Pennsylvania, United States; § Department of Chemical and Petroleum Engineering, University of Pittsburgh, Pittsburgh 15261, Pennsylvania, United States

## Abstract

Recent advancements have shown that in situ stresses can be reliably estimated through an integrated machine/deep learning (ML/DL)-based framework, which relies on models trained and validated using true triaxial ultrasonic velocity (TUV) experimental data that involve measurements of ultrasonic velocity in saturated rocks under varying stress configurations. However, when the goal is to interpret lower frequency measurements, it may be more appropriate to run experiments on dry rocks and then obtain Biot–Gassmann-derived equivalent saturated velocities (low-frequency approximation) and employ these quantities for training ML/DL models to predict in situ stress. Whether the dispersion effect of frequency on the velocity–stress relationship substantially impacts in situ stress prediction is an important and unresolved question. This work presents an enhancement of ML/DL-based workflow by training and implementing ML/DL models using equivalent saturated acoustic velocities (low-frequency) obtained by applying Biot–Gassmann fluid substitution on the ultrasonic velocities of dry cores. The models were trained on TUV data sets derived from three subsurface cores extracted from the geothermal well 16B(78)-32 at the Utah FORGE site. Each core was subjected to 75 unique stress configurations for velocity measurement in the dry state. The ML/DL trained on the TUV data set with equivalent saturated velocities demonstrated promising performance to predict in situ stress in subsurface geological rocks using velocity–stress relationships with *R* ^2^ of 0.86, 0.971, and 0.975 and root mean squared error (RMSE) of 2.59, 1.92, and 1.80 for validation/testing phases of vertical, minimum horizontal, and maximum horizontal stress models, respectively. Additionally, interpretation and explanation by Shapley additive explanations (SHAP) analysis further improved scientific validation and model reliability for estimating in situ stresses.

## Graphical abstract

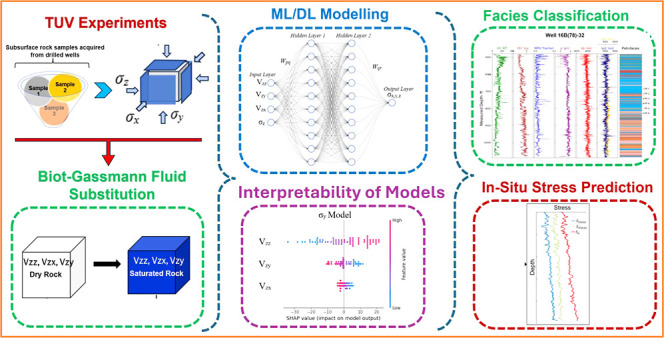

## 1 Highlights

•Comparison of in situ stress prediction results for Biot–Gassmann-derived and measured ultrasonic velocities.
- Model-agnostic explanation of machine learning predictive models for scientific validation
- Exploration of the velocity–stress constitutive relationship by machine learning models
- Integrated workflow to estimate the in situ principal stress in subsurface geological rocks.

## 2 Introduction

A precise estimate of in situ stresses in subterranean rock formations plays a critical role in the planning and execution of various subsurface engineering applications, including geothermal energy production,^1^ mining operations,^2^ coal bed methane production,^3^ groundwater resources,^5^ and tunnel excavation.^4^ Moreover, in situ stress magnitude and direction significantly influence processes such as hydraulic fracture growth, fault reactivation, integrity of wellbores, and stimulation pressure of reservoirs.^1,6^

Several methods have been employed to estimate stresses within subsurface rock formations. Fluid injection techniques implemented in the field including minifrac, leak-off, and diagnostic fracture injection tests are performed to determine the directions and magnitude of the minimum horizontal stress.^7,8^ Additionally, maximum horizontal stress may not be directly measured and is typically inferred using indirect methods.^9,10^ Several other techniques, such as the elastic stiffness method, poroelastic geomechanical models, numerical simulations, and Zoback’s polygon approach, have been developed to generate continuous in situ stress profiles.^11,12^ Nevertheless, these methods require capital investment of time, labor, and money and are prone to uncertainties, mainly owing to the lack of precise tectonic strain data and simplifying assumptions about rock elasticity.

Within this broader context, conducting and interpreting stress tests in geothermal reservoirs present significant challenges primarily due to elevated subsurface temperatures. High-temperature conditions pose a risk of damaging downhole instruments during testing.^13^ Consequently, the use of heat-resistant equipment or the application of wellbore precooling methods becomes necessary, which contributes to higher operational expenses. Moreover, precooling the wellbore can alter the natural thermal stress state, thereby complicating the accurate interpretation of stress measurements.^14,15^

Over the past few years, the applications of artificial intelligence (AI), ML, and DL have dramatically expanded across multiple fields including geo-energy,^16^ tunneling,^17^ geotechnical engineering,^18^ groundwater resource management,^19^ and mining.^20^ Many classification and regression problems within the energy sector have been efficiently addressed through advanced computational methods including artificial neural networks (ANNs), gated recurrent units, convolutional neural networks (CNNs), long-short-term memory, gradient boosting, random forest (RF), and support vector machine (SVM) algorithms.^21–24^ Machine learning-based solutions in this sector offer several benefits including minimized costs, increased productivity, and improved operational efficiency.^16^

Here, we consider a particular workflow that uses machine learning in conjunction with wave velocity measurements in rocks. There are some challenges that arise, which are the motivation for this paper. Elastic wave propagation through the saturated porous rocks results in pressure gradients within pore spaces, which causes a microscopic fluid flow mechanism known as squirt flow. It plays a crucial role in how elastic waves (seismic and acoustic waves) travel through the saturated porous rocks. Due to squirt flow, acoustic waves travel at slightly different velocities at different frequencies. This phenomenon is known as wave dispersion. The interaction between the wave and the fluid flow mechanisms within the pores causes this variation in speed.^25–27^ At higher frequencies, i.e., ultrasonic frequencies, the pore fluid may not have enough time to equilibrate pressure, resulting in higher measured velocities due to increased stiffness compared to the velocities measured at low frequency. Lower-frequency waves might travel deeper than higher-frequency waves traveling through the same rock. Second, energy is dissipated when the fluid squeezes through pore spaces. This energy loss causes the weakening of the wave intensity as they propagate through the rock, resulting in wave attenuation. Pore fluid movement functions as an energy conversion process which transforms wave energy into small fluid motions within pore spaces.^25–28^

The Biot–Gassmann fluid substitution method stands as a widely used approach in rock physics and geomechanics for evaluating seismic wave velocities and elastic moduli alterations caused by different saturating fluids present in porous rocks. It also addresses the dispersion and attenuation effects due to higher frequency components by providing a low-frequency approximation of equivalent velocities. Gassmann (1951)^29^ introduced the initial formulation that was later expanded by Biot (1956)^30^ to establish a direct connection between dry-frame rock measurements and saturated in situ conditions through an analytical formula to compute the bulk modulus of saturated porous rocks based on the properties of the dry rock frame, solid mineral grains, pore fluid, and porosity. The model assumes low-frequency or quasi-static conditions, and hence, pore pressures are assumed to be equilibrated throughout elastic wave propagation, making it specifically relevant in seismic applications (1–100 Hz frequency) and petrophysical logging (10k–20k Hz). Fluid substitution allows the transformation of the seismic response of porous rocks saturated with one type of fluid (e.g., brine) to the response of rocks saturated with another type (e.g., water, oil, or gas). The principle serves as an essential foundation for wide applications in hydrocarbon exploration, reservoir characterization, CO_2_ sequestration, and geothermal energy development.^25,31^ It also comprises an alternative to laboratory testing of saturated rocks at ultrasonic frequencies (100 k-1 M Hz) that overcomes the issue of dispersion that can call into question the degree to which such laboratory-derived wave velocities correspond to the lower frequency velocities relevant to seismic or petrophysical methods.

Elastic wave velocity is fundamentally linked to the elastic moduli of rocks, as the propagation of shear (S-wave) and compressional (P-wave) wave velocities depends on the shear and bulk moduli of the rock matrix. In the context of Biot–Gassmann theory, the elastic wave velocity in porous rocks changes as pore fluid content and stress conditions alter the effective elastic moduli. Gassmann’s equations specifically describe how elastic constants are modified when pore spaces are saturated with fluid, allowing for the computation of saturated elastic moduli and velocities using dry-frame moduli and velocities measured in laboratory experiments. The elastic moduli are affected by effective stress and mineral composition, demonstrating that the acoustic velocity is sensitive to both intrinsic material properties and environmental conditions. Several experimental and theoretical studies reported that alterations in stress or fluid content led to changes in elastic moduli and thus in the acoustic velocities of porous rocks.^25,32–35^

The Biot–Gassmann theory can be applied broadly to various conditions. However, there are a few relevant limitations. Most notably, the mathematical model requires uniform and connected pore spaces and does not hold for fractured systems or chemical interactions between rock and fluid. Nevertheless, the Biot–Gassmann framework provides a first-order approximation to model rock–fluid interactions in subsurface rocks.^25^

While the main focus is on the integration of the Biot–Gassmann fluid substitution into an ML workflow, it is also an objective of the work to demonstrate the interpretability of the resulting ML models. As the applications of ML/DL tools continue to grow across various fields, the need for interpretability and transparency in sophisticated ML/DL models has become increasingly important. Although advanced ML/DL models deliver high predictive performance, they are normally regarded as black boxes due to the nontransparent decision-making mechanisms. This lack of transparency poses a challenge to implementing ML/DL models in crucial scientific domains where understanding the impact of input parameters is vital for scientific credibility and real-world deployment. The new approach of explainable artificial intelligence (XAI) was introduced to resolve this limitation by improving the interpretability of ML/DL models.^36^ Lundberg and Lee (2017)^37^ proposed the Shapley additive explanations (SHAP) that function as a model-agnostic explanation technique developed through cooperative game theory methods. It computes Shapley values to determine the individual influence of each variable on specific predictions, as well as their overall impact on the model’s target prediction, thus allowing comprehensive interpretation of ML/DL models at both global and local scales. Recently, various studies have demonstrated the effectiveness of SHAP analysis for geotechnical and earth sciences domains such as prediction of rock compressive strength and failure behavior,^38^ assessment of landslide susceptibility,^39^ and groundwater contamination.^40^

This study presents the implementation of the ML/DL-based framework, previously proposed by Mustafa et al. (2024),^41^ employing ML/DL models (supervised and unsupervised) to predict the in situ principal stresses in subterranean geological rocks utilizing laboratory-based true triaxial ultrasonic velocities (TUV) and field-based well logs. The effectiveness of the ML-based workflow was previously demonstrated using ultrasonic wave velocities of saturated core samples (obtained from TUV experiments on saturated cores) and sonic logs data for two different wells, namely, 16A(78)-32 ^41^ and 16B(78)-32 ^42^ of the FORGE geothermal reservoir. Unlike previous studies, this work follows a unique approach of implementing a similar ML-based workflow for estimating in situ stress by leveraging a new suite of modified TUV data encompassing equivalent saturated acoustic velocities (low-frequency approximation) and well log data. The equivalent saturated acoustic velocities (low-frequency approximation) were obtained from the Biot–Gassmann fluid substitution method using ultrasonic velocities of dry core samples. Despite various applications of the Biot–Gassmann fluid substitution model to study the subsurface rock characteristics and seismic response, this work is a unique application of the Biot–Gassmann fluid substitution model to obtain equivalent saturated acoustic velocities from dry ultrasonic velocities, which is in turn used for the development of ML models of stress estimation. Although the Biot–Gassmann fluid substitution model is extensively used for reservoir characterization, seismic monitoring, etc., it has not previously been used in such a unique application as in this study. This novel workflow is capable of delivering accurate in situ stress estimations in subsurface rock formations. The study demonstrates the applicability of the modified TUV data set to develop DL/ML models with excellent accuracy of predicted in situ stresses in a geothermal production well 16B(78)-32. This work also encompasses the comparative analysis of the predicted in situ stress that resulted in this study with the previously reported results by ref 42. Moreover, the explainability and interpretability of ML/DL predictive models were illustrated using the SHAP algorithm to understand the models’ prediction mechanism for scientific validation. The underlying physics of the models is also elaborated by the sensitivity/parametric study of the models.

## 3 Methodology

This study implements the DL/ML-based workflow recently proposed by Mustafa et al. (2024; 2026)^41,42^ with a suite of new laboratory triaxial ultrasonic velocity (TUV) data obtained from dry rock samples. The ML/DL workflow predicts the principal stresses in geological formations by integrating supervised and unsupervised ML/DL algorithms with the Biot–Gassmann fluid substitution method. Detailed discussion about the ML/DL-based workflow is illustrated in Mustafa et al. (2024)^41^ and Mustafa et al. (2026).^42^ Furthermore, SHAP analysis was performed to explore the interpretability and explainability of the DL/ML predictive models developed using modified TUV data.

In this work, initial ultrasonic wave velocities from dry core samples are obtained from TUV experiments performed on the dry cores. The TUV laboratory experiments were performed on three subsurface cores, obtained from different depths in well 16B(78)-32, for generating a TUV data set for each individual core. The subsurface core samples represent three different types of rocks: quartz gneiss, granite, and gneiss. Overall, 225 TUV tests were conducted using three core samples, with 75 tests on each individual core. The number of TUV experiments on each individual core sample sufficient to generate generalized ML/DL models was determined by learning curve analysis in our previous study by Mustafa et al. (2026).^42^ The following section provides more details about the TUV experimental procedures. Then, the Biot–Gassmann fluid substitution method was employed to ultrasonic velocities of dry core samples in order to obtain equivalent saturated acoustic velocities (low-frequency approximation). The TUV data set with equivalent saturated velocities is referred to as the modified TUV data set. The objective of obtaining equivalent saturated acoustic velocities (low-frequency approximation), using the Biot–Gassmann fluid substitution method, is to address the dispersion and attenuation effects caused by the squirt flow of pore fluid during the propagation of high-frequency ultrasonic wave velocities. Finally, the Biot–Gassmann-derived equivalent saturated velocities were employed to construct ML/DL predictive models. The data sets used in this work including the modified TUV data set and suite of well logs are associated with a geothermal well, 16B(78)-32, located at the Utah FORGE geothermal site.

A sequence of analyses was performed using modified TUV data sets, including exploratory data analytics (EDA), construction of DL/ML predictive models, parametric/sensitivity analysis, and SHAP analysis of the predictive models. The DL/ML models were constructed for three principal stresses, such as two mutually perpendicular horizontal (σ_*x*_ and σ_*z*_) and vertical (σ_*y*_) stresses. In total, five ML/DL (two ML and three DL) algorithms were adopted—gated recurrent units (GRUs), convolutional neural networks (CNNs), extreme gradient boosting (XGB), random forest (RF), and deep neural networks (DNNs). The ML/DL methods, similar to our previous study,^41^ were employed for better comparison and models’ applicability on both types of TUV data sets. It is critical to note that distinct predictive models of three principal stresses were developed for each individual core using the corresponding TUV data.

Further, predictive models with the best prediction performance and generalization capabilities were selected for the SHAP analysis. SHAP analysis was performed to demonstrate how the input features contributed and interacted in the decision-making mechanism of the DNN models (developed using the modified TUV data set). Thus, global and local interpretations of the predictive models were illustrated. The ML/DL models’ development and SHAP analysis were performed using Python.

Similar to the previous workflow,^41^ the unsupervised ML algorithm K-means was applied for classification of subsurface geological formation into a plurality of petrofacies/clusters based on well log properties, and thereby, petrofacies corresponding to the depths of three subsurface cores were also identified. Subsequently, in situ stresses were predicted by applying modified TUV-based ML/DL models using field sonic logs of the same well. Finally, the results obtained in this work were compared with the previously reported results by ref 42. The entire workflow followed in this study is illustrated in Figure 1. A brief discussion about the ML/DL methods and Biot–Gassmann fluid substitution method is provided before a detailed description of these phases.

**Figure 1:**
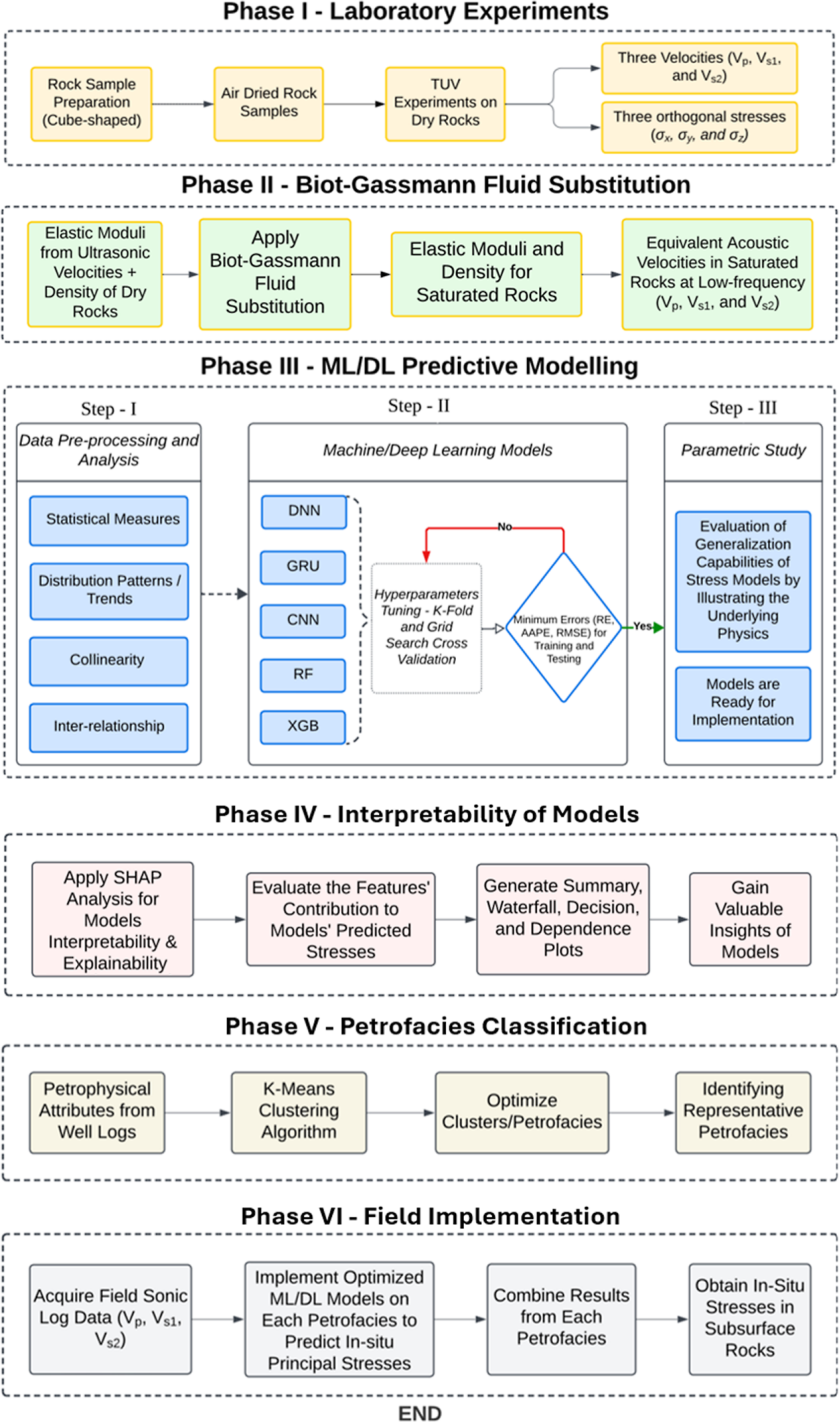
Workflow diagram showing stepwise methodology of this study.

### 3.1 True Triaxial Ultrasonic Velocity Experiments

In this study, a series of TUV laboratory experiments were conducted on subsurface cores to build sufficient data for each individual core, enabling the development of reliable ML/DL predictive models for vertical and two orthogonal horizontal stresses. A detailed experimental procedure of the TUV tests can be found in Mustafa et al.^42^ The core samples were collected from three distinct locations within well 16B(78)-32, encompassing various rock types, such as gneiss, quartz gneiss, and granite. It is worth noting that well 16B(78)-32 includes both vertical and deviated sections, with all samples obtained from the deviated part. As a result, the stress orientations in the local (deviated) coordinate system must be converted to align with the global (in situ principal) stress directions using a three-dimensional (3D) stress transformation. Table 1 summarizes the measured depths and key properties of the core samples. The dynamic Poisson’s ratio and Young’s modulus were computed from ultrasonic velocities measured on dry rock samples.

**Table 1:**
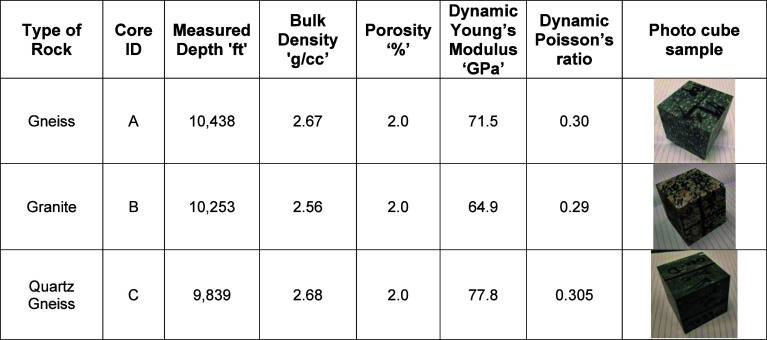
Depths, Important Characteristics, and Photos of 2.6 in. Cube Samples Used for TUV Experiments

The laboratory testing was carried out in sequential stages, including preparation of the sample, drying, and execution of TUV experiments. Cores were cautiously cut and ground to finally obtain cube-shaped specimens with a dimension of 2.6 in. (0.066 m) with a tolerance of 0.001 in. (2.54 × 10^–5^ meters), ensuring perpendicularity of all sides. These samples were then air-dried for 24 h. Then, dry core samples were utilized to conduct TUV tests. It is worth noting that the performance of TUV experiments on fully saturated cores, although intuitive for replicating in situ conditions, introduces velocity dispersion at ultrasonic frequencies due to local (squirt) flow between compliant pores and stiff pores. This dispersion can artificially inflate saturated velocities relative to the low-frequency limit of sonic log measurements. The workflow presented here addresses this issue by (1) conducting all velocity measurements on dry cores (eliminating squirt flow) and (2) applying the Biot–Gassmann theory to obtain equivalent saturated velocities at the low-frequency limit, thereby capturing genuine fluid-saturation effects without experimental artifacts.

The TUV experiments involve recording the travel time of two shear (S-) waves polarized in orthogonal orientations and compressional (P-) waves under numerous configurations of true triaxial stresses. Acoustic transducers, mounted on steel platelets positioned on each face of the cube-shaped sample, were used to transmit and receive ultrasonic signals. The corresponding S- and P-wave velocities were then calculated by using the dimensions and recorded travel times of the cube-shaped samples.

This study focuses on the S- and P-wave velocities propagating along the *z*-direction. True triaxial compression stresses σ_*x*_, σ_*y*_, and σ_*z*_ (independently controlled) were applied along the three mutually orthogonal axes (*x*, *y*, and *z*) of the cube-shaped samples, corresponding to the principal stress directions in TUV experiments. The P-wave velocity measured in the *z*-direction is labeled as *V*_*zz*_, while *V*_*zx*_ and *V*_*zy*_ represent two S-wave velocities polarized in the *y* and *x* directions, respectively, all traveling along the *z*-axis. Typically, *V*_*zy*_ and *V*_*zx*_ are described as velocities of slow and fast shear waves, since their polarities align with two orthogonally oriented stresses. The configuration of the triaxial stress in the TUV setup is illustrated in Figure 2a,b, and a schematic demonstration is provided in Figure 2c.

**Figure 2:**
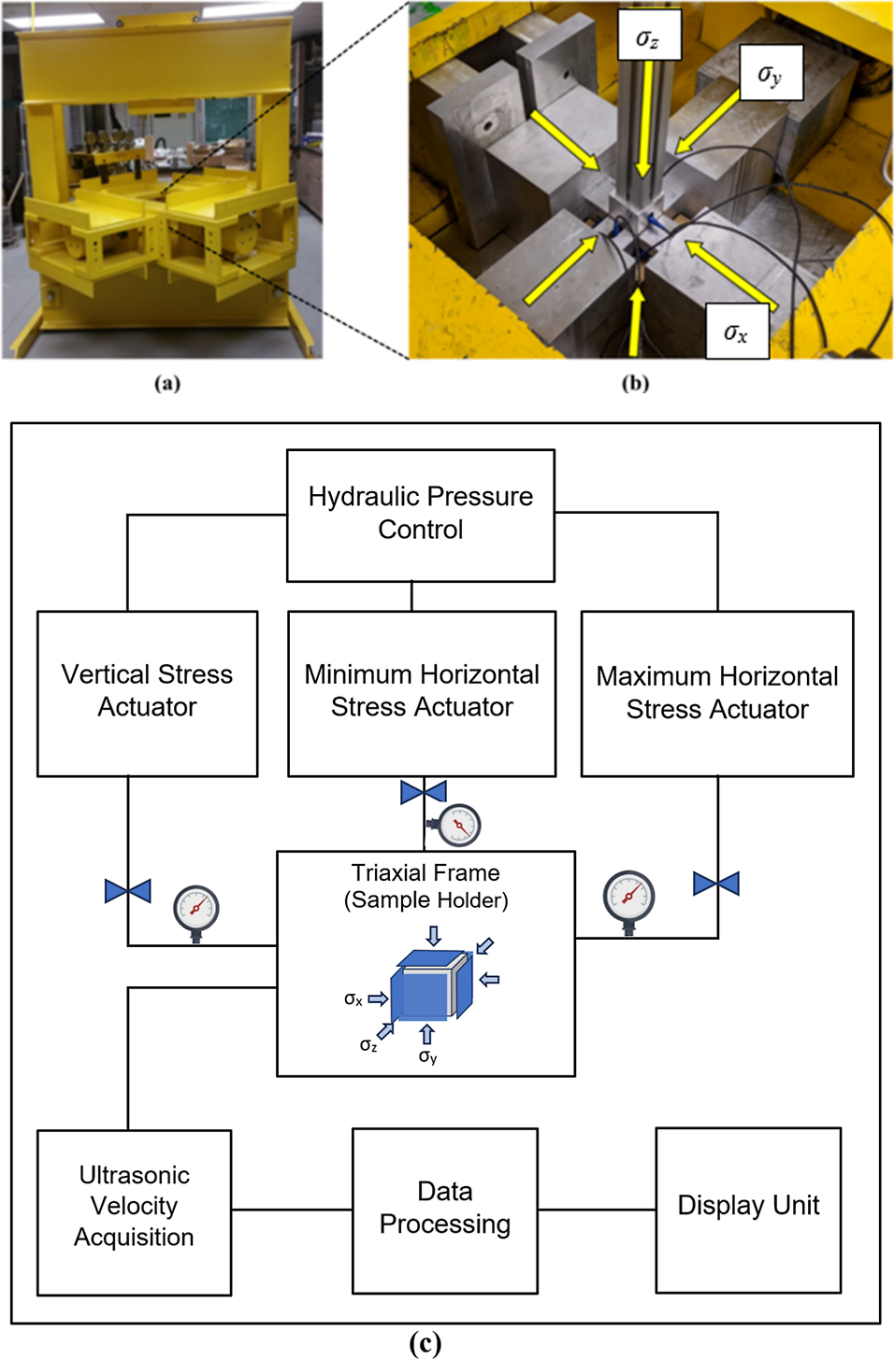
Laboratory setup for TUV experiments; (a) true triaxial compression system; (b) loading frame with an installed rock sample; (after Bunger et al.^43^) (c) schematic illustration of the TUV experimental setup.

### 3.2 Biot–Gassmann Fluid Substitution Method

The Biot–Gassmann fluid substitution method is a theoretical approach for approximating equivalent saturated, low-frequency acoustic velocities utilizing the characteristics of the dry rock frame, solid mineral matrix, fluid, and porosity. This work applies this method to the measured ultrasonic S- and P-wave velocities of dry subsurface core samples to derive their equivalent saturated acoustic velocities, facilitating the analysis of fluid effects under reservoir conditions.

The Biot–Gassmann fluid substitution method is comprised of multiple steps as illustrated by refs 25, 44, and 45 . The steps of the method followed in this work are summarized in Table 2. First of all, bulk modulus (*K*_dry_) and shear modulus (μ_dry_) of dry core samples were computed using laboratory-based ultrasonic velocities (measured in TUV tests) and density of dry rocks using eqs 1 and 2. These moduli represented the stiffness of the rock frame without pore fluid. Then, the bulk modulus of the mineral matrix (*K*_s_) was determined using the Voigt–Ruess–Hill (VRH) average (eqs 3–5). Bulk moduli of individual minerals (*K*_i_) and their corresponding volumetric fractions were combined to estimate Voigt (upper) and Reuss (lower) bounds, and their mean was computed as illustrated in eqs 6 and 7. The percentage fraction of the different minerals present at the core locations in well 16B(78)-32 was obtained from X-ray diffraction (XRD) of subsurface core samples as reported by ref 46. The corresponding bulk moduli of individual minerals (*K*_i_) were obtained from ref 25. Further, bulk moduli of fluid (*K*_f_) and density (ρ_f_) can be computed using the weighted harmonic (Ruess) and weighted arithmetic mean, respectively. It is worth mentioning that pore spaces are considered to be filled with the groundwater encountered in the deep wells at the Utah FORGE site.^47–49^ Thus, the density (ρ_f_) and fluid bulk modulus (*K*_f_) of the encountered groundwater were utilized. Subsequently, Gassmann’s fluid substitution equation was employed to derive the bulk modulus (*K*_sat_) of rock under fluid saturation condition using porosity and the elastic moduli of dry rock, pore-filling fluid, and mineral matrix, as demonstrated in eq 8. It is crucial to note that the shear modulus remained unaltered by fluid content, as illustrated in eq 9. The bulk density of saturated rock (ρ_sat_) was computed using eq 10. Finally, the equivalent saturated P- and S-wave (*V*_p(sat)_ and *V*_s(sat)_) velocities were obtained using eqs 11 and 12, respectively.

**Table 2:** Input and Computed Parameters in the Biot–Gassmann Fluid Substitution Method^25^

step no	parameter description	governing equations	equation no
1	elastic moduli of dry rock	1 $K_{dry}=\left({\left(V_{p\left(dry\right)}\right)}^{2}-\frac{4}{3}{\left(V_{s\left(dry\right)}\right)}^{2}\right)$	equation 1
		*V*_p(dry)_ = P-wave velocity measured in dry rock; *V*_s(dry)_ = S-wave velocity measured in dry rock; *K*_dry_ = bulk modulus of dry rock.	
		2 $\mu_{dry}=\left(\rho_{dry}{\left(V_{s\left(dry\right)}\right)}^{2}\right)$	equation 2
		*V*_s(dry)_ = S-wave velocity measured in dry rock; μ_dry_ = shear modulus of dry rock; ρ_dry_ = density of dry rock.	
2	Voigt–Reuss–Hill averaging—bulk modulus of the mineral matrix	3 $K_{V}=\sum_{i}V_{i}K_{i}$	equation 3
		4 $\frac{1}{K_{R}}=\sum_{i}\frac{V_{i}}{K_{i}}$	equation 4
		5 $K_{s}=\frac{K_{V}+K_{R}}{2}$	equation 5
		*K*_V_ = Voigt average of mineral bulk modulus; *K*_R_ = Ruess average of mineral bulk modulus; *K*_*s*_ = bulk modulus of minerals; *K*_*i*_ = bulk modulus of *i*th mineral; *V*_*i*_ = volume fraction of *i*th mineral.	
3	fluid bulk modulus and density	6 $\frac{1}{K_{f}}=\sum_{i}\frac{S_{i}}{K_{f,i}}$	equation 6
		7 $\rho_{f}=\sum_{i}S_{i}\rho_{f}$	equation 7
		*K*_f_ = bulk modulus of fluid; ρ_f_ = density of fluid.	
4	Gassmann fluid substitution—bulk modulus of saturated rock	8 $K_{sat}=K_{dry}+\frac{{\left[\phi \left(K_{f}-K_{dry}\right)\right]}^{2}}{\phi \left(K_{f}-K_{dry}\right)+\left(1-\phi \right)\left(K_{s}-K_{dry}\right)}$	equation 8
		*K*_dry_ = bulk modulus of dry rock; *K*_s_ = bulk modulus of minerals; *K*_f_ = bulk modulus of fluid; ϕ = porosity of rock; *K*_sat_ = bulk modulus of saturated rock.	
5	shear modulus	9 $\mu_{sat}=\mu_{dry}$	equation 9
		μ_dry_ = shear modulus of dry rock; μ_sat_ = shear modulus of saturated rock.	
6	saturated density	10 $\rho_{sat}=\left(1-\phi \right)\rho_{s}+{\phi \rho }_{f}$	equation 10
		ρ_f_ = density of fluid; ϕ = porosity of rock; ρ_sat_ = density of saturated rock; ρ_s_ = density of solid minerals.	
7	fluid-substituted acoustic wave velocities	11 $V_{p\left(sat\right)}=\sqrt{\frac{K_{sat}+\frac{4}{3}\mu_{sat}}{\rho_{sat}}}$	equation 11
		12 $V_{s\left(sat\right)}=\sqrt{\frac{\mu_{sat}}{\rho_{sat}}}$	equation 12
		*K*_sat_ = bulk modulus of saturated rock; μ_sat_ = shear modulus of saturated rock; ρ_sat_ = density of saturated rock; *V*_p(sat)_ = equivalent P-wave velocity for saturated rock; *V*_s(sat)_ = equivalent S-wave velocity for saturated rock.	

### 3.3 Machine Learning/Deep Learning Methods

This study employed five supervised DL/ML techniques, including two ML and three DL, to construct prediction models of three principal stresses, i.e., σ_*x*_, σ_*y*_, and σ_*z*_. The employed algorithms incorporate CNN, XGB, RF, GRU, and DNN. Note that the distinct models were constructed for each of the principal stress components (σ_*x*_, σ_*y*_, and σ_*z*_) using modified TUV data sets of each core sample individually. Furthermore, the K-means clustering technique was employed to classify the geological formations into a plurality of petrofacies/rock facies. A comprehensive explanation of the principles and operation of each algorithm employed in this study is elaborated in Appendix A.

### 3.4 Data Description

This study employed two distinct types of data sets: core-based experimental TUV data and field-based well log data. The TUV data set was used for training and validating the ML/DL models. This data set includes P-wave and two S-wave velocities (fast- and slow-shear), i.e., as *V*_*zz*_, *V*_*zy*_, and *V*_*zx*_, respectively, measured on dry rock samples subjected to numerous configurations of true triaxial stresses. The measured velocities of dry core samples were then modified to equivalent saturated acoustic velocities using the Biot–Gassmann fluid substitution method. A total of 225 TUV experiments were conducted, 75 tests for each of the three core samples, providing a comprehensive data set for model training and validation.

In the context of a field scenario where the axis of a deviated or horizontal well is aligned along the minimum principal stress direction, the orientation of core samples is such that the σ_*y*_ stress in the TUV experiment corresponds to vertical stress, while σ_*x*_ and σ_*z*_ represent the two orthogonally oriented horizontal stresses. The training and validation of DL/ML models to predict σ_*y*_ (vertical stress) were executed using three input variables such as *V*_*zx*_, *V*_*zy*_, and *V*_*zz*_, whereas models for horizontal stresses (σ_*z*_ and σ_*x*_) were trained using four input variables including *V*_*zy*_, *V*_*zz*_, *V*_*zx*_, and σ_*y*_. It is important to mention that vertical stress (σ_*y*_), which can be reliably estimated in the field using density log, was an additional known input for predicting the horizontal stresses (σ_*z*_ and σ_*x*_). Including σ_*y*_ as an input feature may improve prediction accuracy, as it plays a crucial role in generating horizontal stress within subsurface rocks. The graphical presentation of the modified TUV data points for cores A, B, and C is given in Appendix B, where a suite of features’ values in each row of plots corresponds to a single TUV test.

It is crucial to note that the predictive models can only be applied to the corresponding petrofacies and rock facies that share similar characteristics with core samples used to perform TUV experiments. This ensures the rock types (petrofacies) associated with the training of models are also used for prediction. Therefore, petrophysical properties derived from well logs such as neutron porosity (ϕ), bulk density (ρ), gamma ray (GR), and photoelectric factor (PEF) were utilized to classify subsurface formations into distinct petrofacies within the measured depth range of 4835 to 10872 feet in well 16B(78)-32. Following this classification, the bulk density and sonic logs, acquired from the same interval of depth, were used for executing the field application of the trained ML/DL models.

### 3.5 Exploratory Data Analysis

Prior to the construction of DL/ML models, an extensive exploratory data analysis (EDA) was conducted to better understand the features of the data set. EDA provides critical information regarding statistical behavior, distribution patterns, interfeature relationships, and the relative importance of the input variables. Descriptive statistical measures including mode, median, mean, range, kurtosis, skewness, and standard deviation are summarized in Table 3. The distribution and pattern of input and output variables for core A are illustrated through histogram plots in Figure 3, while corresponding plots for cores B and C are presented in Appendix C. In addition, violin plots were employed to visually demonstrate the distribution of data using kernel density estimates, highlighting features such as interquartile ranges, means, extreme values, and the modality of distributions (unimodal, bimodal, or multimodal). These violin plots for all three cores are included in Appendix D. To examine the degree of linear and nonlinear associations among features, correlation heat maps were generated using Pearson, Kendall, and Spearman methods, as demonstrated for core A in Figure 4. The heat maps for cores B and C are provided in Appendix E. Most feature pairs showed a positive correlation. The mathematical formulations for the three correlation techniques are also presented.

13 $$\rho_{pearson}=\frac{k\sum xy-\left(\sum x\right)\left(\sum y\right)}{\sqrt{k\left(\sum x^{2}\right)-{\left(\sum y\right)}^{2}}\sqrt{k\left(\sum y^{2}\right)-{\left(\sum y\right)}^{2}}}$$

14 $$\rho_{spear\;man}=\rho_{pearson}\frac{cov\left(x,y\right)}{\gamma_{x}\gamma_{y}}$$

15 $$\tau_{kendall}=\frac{n_{c}-n_{d}}{n\left(n-1\right)/2}$$

**Table 3:** Statistical Indicators of the Data Set of Core A

statistical indicators	σ_*x*_ (MPa)	σ_*y*_ (MPa)	σ_*z*_ (MPa)	*V*_*zz*_ (m/s)	*V*_*zx*_ (m/s)	*V*_*zy*_ (m/s)
maximum	22.5	22.5	20	5658.6	3107.8	2933.1
minimum	67.5	89.4	55	6232.9	3327.5	3114.5
median	47	56.1	43.5	6014.0	3249.1	3034.4
mode	50	50	45	6157.7	3175.7	3108.9
mean	45.0	58.5	39.6	6003.3	3234.6	3027.4
St. Dev.	12.0	17.1	9.6	148.1	54.1	48.4
kurtosis	–1.0	–1.0	–0.8	–0.9	–0.6	–0.7
skewness	–0.1	0.1	–0.5	–0.3	–0.5	–0.1

**Figure 3:**
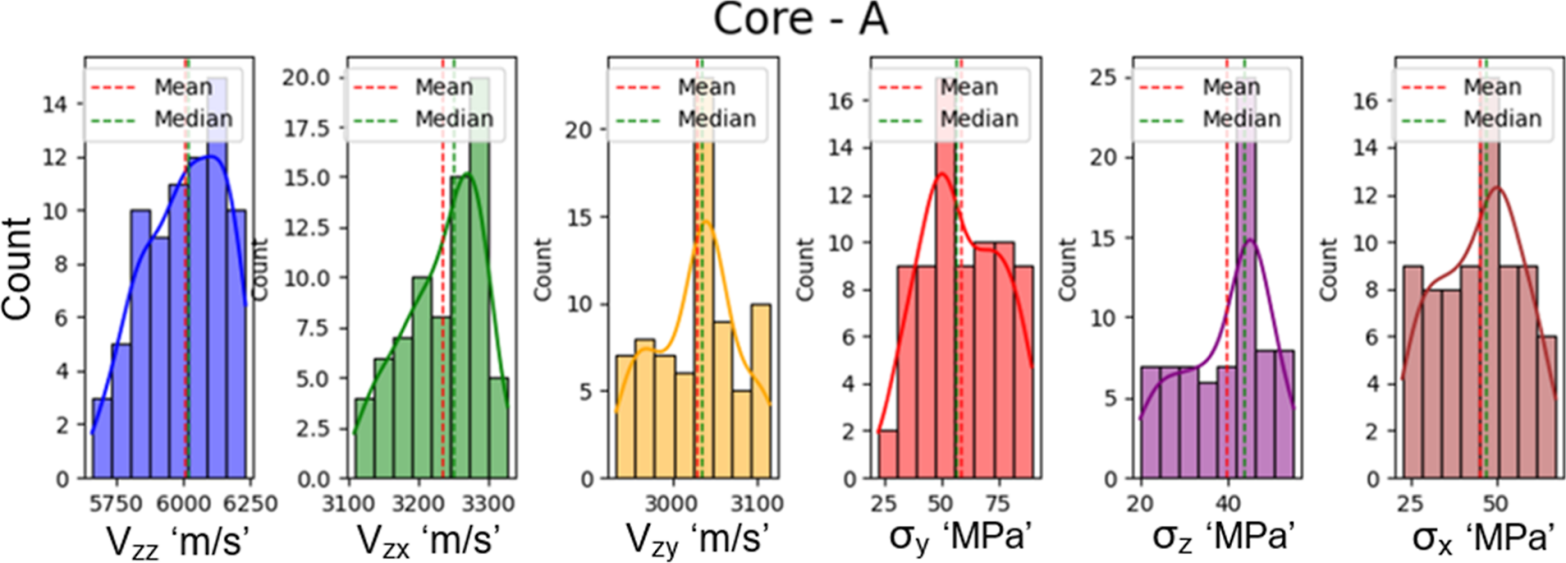
Data distribution shown by histogram plots of output and input features of the TUV data set of core A.

**Figure 4:**
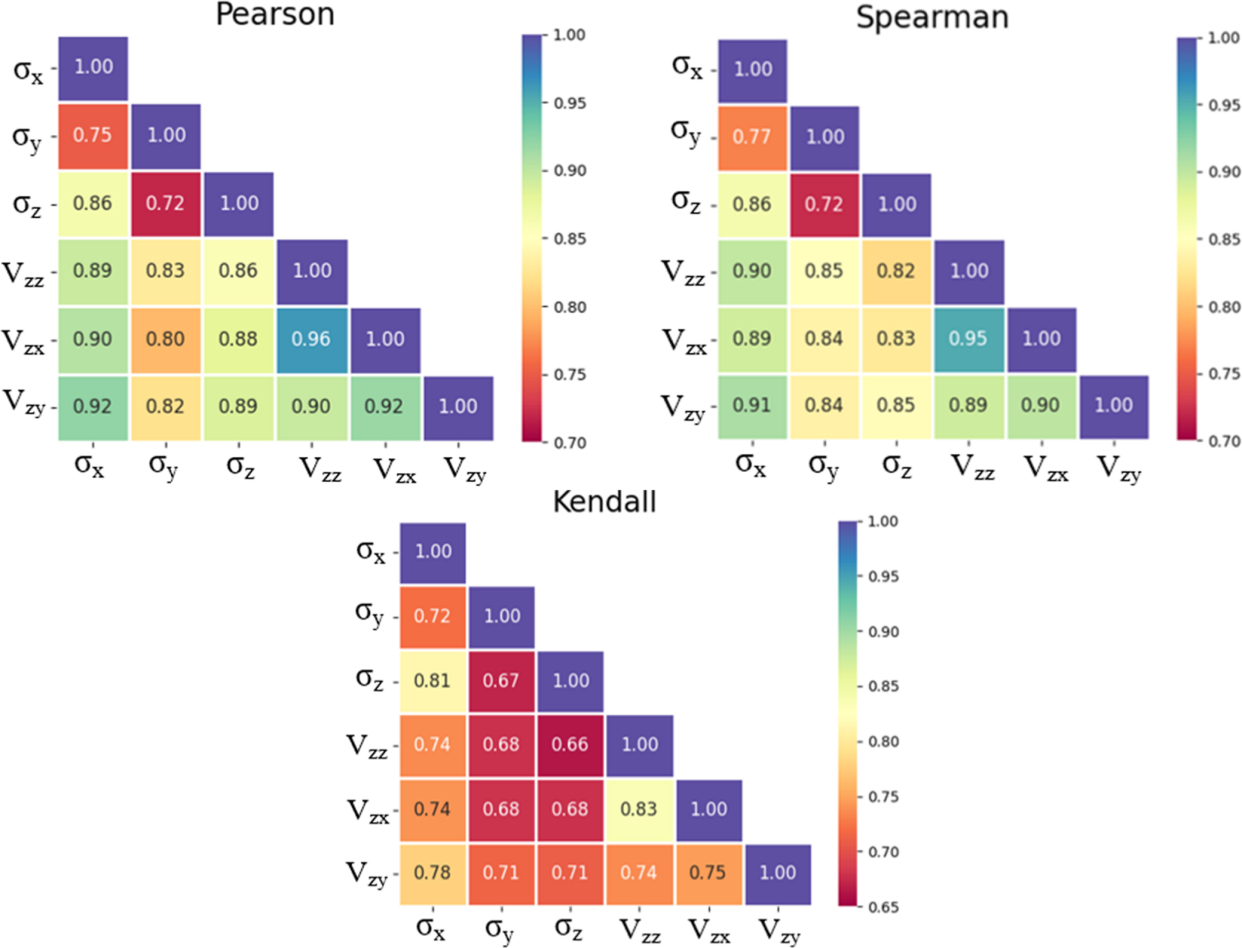
Collinearity between the pair of features for core A is shown as heat maps.

Here, corresponding variables are represented by *x* and *y*, and *k* shows the sample size. Standard deviations (St. Dev.) of the *y* and *x* variables are shown as γ_*y*_ and γ_*x*_, respectively, and cov­(*x*,*y*) shows the covariance. *n*_d_ and *n*_c_ represent the numbers of discordant and concordant pairs of variables (*x* and *y*), respectively, and *n* shows the number of data points.

The relative importance of all input variables in relation to output stresses for σ_*x*_, σ_*y*_, and σ_*z*_ models of cores A, B, and C are illustrated in Appendix F. The methods used to determine feature importance are illustrated in eqs 13–15. The performance of the ML models is closely tied to the feature importance scores. Input features *V*_*zx*_, *V*_*zy*_, and *V*_*zz*_ demonstrated a strong positive correlation with the stress σ_*y*_. The features *V*_*zx*_ and *V*_*zz*_ showed a comparatively weaker correlation than *V*_*zy*_. Similarly, *V*_*zy*_ was observed to have a slightly stronger correlation with σ_*z*_ and σ_*x*_ than *V*_*zx*_ and *V*_*zz*_. Additionally, vertical stress (σ_*y*_) was observed to have a relatively weaker correlation with two horizontal stresses σ_*x*_ and σ_*z*_ compared to velocities (*V*_*zz*_, *V*_*zx*_, and *V*_*zy*_). Further, pairwise scatter plots between input and output variables confirmed that the velocities (*V*_*zx*_, *V*_*zy*_, and *V*_*zz*_) are significantly impacted by the compressive stresses. The cross plots of all output and input features in the form of a pair plot are provided in Appendix F.

### 3.6 Evaluation Metrics

To measure the prediction accuracy of the ML/DL models, graphical representations and statistical accuracy indicators were utilized. The performance indicators including the coefficient of determination (*R* ^2^), average absolute percentage error (AAPE), residual error (RE), and root mean squared error (RMSE) were applied to quantify the models’ accuracies. The mathematical formulations of these metrics are presented as

16 $$R^{2}={\left[\frac{k\sum ab\;-\left(\sum a\right)\left(\sum b\right)}{\sqrt{\left(k\left(\sum a^{2}\right)-{\left(\sum a\right)}^{2}\right)}\sqrt{k\left(\sum b^{2}\right)-{\left(\sum b\right)}^{2}}}\right]}^{2}$$

Here, *k* denotes the total sample count, whereas *a* and *b* represent the total number of variables, respectively.

17 $$Residual\;Error=\left(\sigma_{measured}-\sigma_{predicted}\right)$$

18 $$AAPE\left(\%\right)=\frac{\sum \left|\left(\sigma_{measured}-\sigma_{predicted}\right)/\frac{100}{\sigma_{measured}}\right|}{total\;number\;of\;data\;points}$$

19 $$RMSE=\sqrt{\frac{\sum {\left(\sigma_{measured}-\sigma_{predicted}\right)}^{2}}{total\;number\;of\;data\;points}}$$

Here, σ_measured_ and σ_predicted_ represent experimental and predicted values of stress, respectively.

Additionally, the accuracy of the clustering model was measured through silhouette index (SI), which is a common measure to quantify how well the data points fit in the assigned group or cluster compared to the rest of the clusters.^50^ SI is mathematically stated as

20 $$SI=s\left(j\right)=\frac{\left(u\left(i\right)-v\left(i\right)\right)}{\mathrm{Max}\left\{v\left(i\right),u\left(i\right)\right\}}$$

Here, variable *u*(*i*) is the calculated average distance from a point “*j*” to all neighboring points in the same cluster “*X*_*i*_”. The metric *v*(*i*) measures the smallest average distance of point “*j*” to all points in any other cluster “*X*_*k*_”. The evaluation metrics are mathematically expressed by eqs 16–20.

## 4 Results and Discussion

### 4.1 ML/DL Model Development and Optimization

The ML/DL models were constructed to predict three principal stresses, i.e., vertical stress (σ_*y*_) and two orthogonally oriented horizontal stresses (σ_*z*_ and σ_*x*_), utilizing modified TUV data. As stated previously, three input parameters *V*_*zx*_, *V*_*zy*_, and *V*_zz,_ were employed for constructing the predictive model of stress σ_*y*_. On the other hand, models of two horizontal stresses (σ_*z*_ and σ_*x*_) incorporated four parameters including *V*_*zx*_, *V*_*zy*_, *V*_*zz*_, and σ_*y*_ as inputs. To ensure consistency in feature magnitudes, Min–Max scaling was implemented for data normalization during the preprocessing. Further, the data was split into two portions, i.e., training and testing/validation sets, through a stratified random sampling method to preserve the distribution and minimize biased models’ performance. The data set was partitioned in such a way that training was performed on 70% while validation was performed on the leftover 30% of the data set.

A grid search cross-validation method was applied to tune the hyperparameters of the ML/DL models. Further details about this approach are illustrated in ref 51. This approach involved multiple training iterations to identify the optimal set of hyperparameters that would yield the highest predictive performance. To ensure consistent and reproducible results, a random seed was applied during the model training. Additionally, to prevent issues of overfitting or underfitting and to further enhance model accuracy, the *k*-fold cross-validation method was implemented. In this method, the training set of data was arbitrarily divided into “*k*” equal-sized subsets, known as folds. According to the findings of Belyadi and Haghighat,^52^ optimal performance is typically observed when “*k*” ranges between 5 and 10. In this work, training data was split into seven random folds (*k* = 7). For each iteration, 6-fold (portions) were utilized for training, while the one leftover set was reserved for testing/validation, with each portion of data undergoing the validation process during the entire training process. The ultimate evaluation was based on the average prediction error of all folds. Detailed procedures for constructing and optimizing each of the DL/ML predictive models (CNN, GRU, XGB, DNN, and RF) are provided in Appendix G.

The training and validation prediction errors (RMSE and *R* ^2^) for different numbers of neuron configurations used in the DNN models of stresses σ_*x*_, σ_*y*_, and σ_*z*_ for core A are illustrated in Figure 5. The sensitivity analyses of neuron counts in hidden layers of DNN models of principal stresses (σ_*z*_, σ_*x*_, and σ_*y*_) constructed for cores B and C are provided in Appendix H. The most effective set of hyperparameters of DNN models for core A was determined as summarized in Table 4. The optimized hyperparameter values of the models corresponding to cores B and C are detailed in Appendix I.

**Figure 5:**
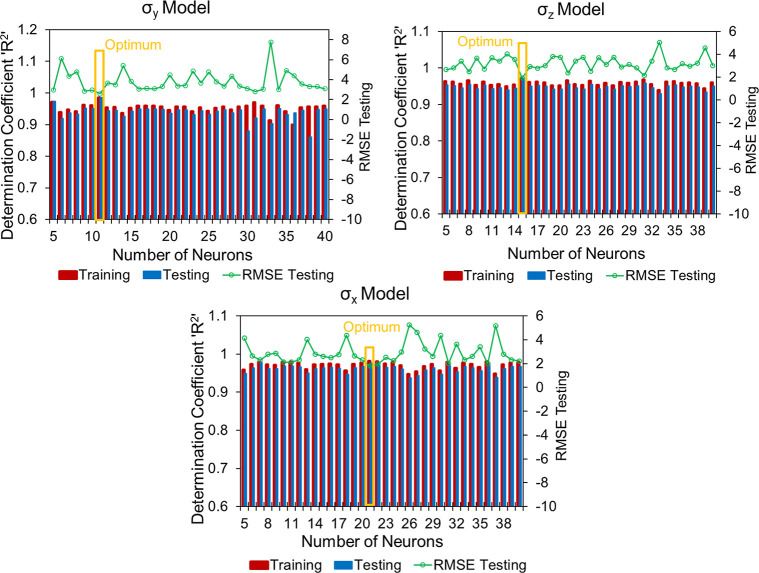
Sensitivity of neuron counts in hidden layers by comparing evaluation measures of σ_*x*_, σ_*y*_, and σ_*z*_ models.

**Table 4:** Tested and Selected Hyperparameters’ Values for the Proposed ML/DL Models of Core A

model type	hyperparameter	tested values	selected value for “σ_*z*_”	selected value for “σ_*y*_”	selected value for “σ_*x*_”
DNN					
	hidden layer count	1–5	2	2	2
	neuron count	5–40	11	21	15
	activation function	tan h, LeakyReLU, ReLU	ReLU	LeakyReLU	tan h
	dropout Rate	0.1–0.45	0.15	0.15	0.15
	optimizer	Nadam, Adam, Sgd	Nadam	Nadam	Adam
	learning rate	0.1–0.00001	0.001	0.0001	0.0001
	loss function	RMSE, MSE	MSE	MSE	MSE
	batch size	64, 32, 16, 8	8	16	8
GRU					
	GRUs	10–220	40	50	45
	optimizer	Nadam, sgd, Adam	Nadam	Nadam	Nadam
	learning rate	0.1–0.00001	0.005	0.005	0.005
	activation function	tan h, LeakyReLU, ReLU	LeakyReLU	ReLU	ReLU
	batch size	64, 32, 16, 8	16	16	16
	dropout rate	0.1–0.45	0.15	0.20	0.15
CNN					
	number of filters	8–32	10	8	8
	kernel size	2–12	4	4	4
	batch size	64, 32, 16, 8	16	8	8
	dense units	8–64	32	16	16
	optimizer	Nadam, sgd, Adam	Adam	Adam	Adam
	activation function for the convolution layer	tan h, LeakyReLU, ReLU	LeakyReLU	Tan h	ReLU
RF					
	sample count necessary to split the internal node	2–15	10	10	8
	sample count at the leaf node	1–6	2	2	2
	total count of trees in the forest	50–2000	900	800	1000
	maximum depth of the trees	3–12	6	5	6
XGB					
	criterion	-	Friedman mse	Friedman mse	Friedman mse
	count of boosting stages to be performed	50–1200	220	150	250
	minimum sample split	1–6	3	3	2
	minimum sample leaf	1–6	2	3	2
	alpha	-	0.02	0.02	0.03
	learning rate	0.1–0.0001	0.001	0.001	0.001
	max. depth	3–12	5	6	5

Excellent prediction performance was observed for the developed predictive models CNN, GRU, DNN, XGB, and RF, reflecting high *R* ^2^ scores and minimal error metrics. However, consistently superior performance was demonstrated by DNN models compared to the rest of the four models of σ_*x*_, σ_*y*_, and σ_*z*_ stresses. The DNN model developed for predicting σ_*y*_ stress achieved an RMSE of 2.04 MPa and an AAPE of 3.46% for the training phase, whereas the validation set yielded an AAPE of 3.97% and an RMSE of 2.59 MPa. The corresponding *R* ^2^ values were found to be 0.985 for training and 0.986 for testing and validation, indicating excellent predictive accuracy. For the stress component σ_*x*_, the DNN model exhibited AAPE values of 3.20% and 3.29%, RMSE values of 1.80 and 1.73 MPa, and *R* ^2^ scores of 0.975 and 0.982 for the testing/validation and training phases, respectively. Additionally, the DNN model exhibited the highest performance for σ_*z*_ stress as well, reflecting RMSE of 1.92 and 1.84 MPa and AAPE of 4.49% and 3.98% for testing/validation and training, respectively. The cross plots between experimental (actual) and predicted stress values for σ_*x*_, σ_*y*_, and σ_*z*_ reflect the models’ performance as shown in Figure 6. The cross plots for cores B and C are presented in Appendix J. The comparison between the experimental and predicted stress values of cores A, B, and C is shown in Appendix K. Predicted stress values demonstrated good agreement with experimentally measured stresses. Additionally, KDE and histogram plots for RE of the DNN predictions are shown in Figure 7 for core A and in Appendix L for cores B and C, exhibiting bell-shaped distributions with peak frequencies corresponding to minimal RE, which reflected excellent predictive performance of the ML/DL models.

**Figure 6:**
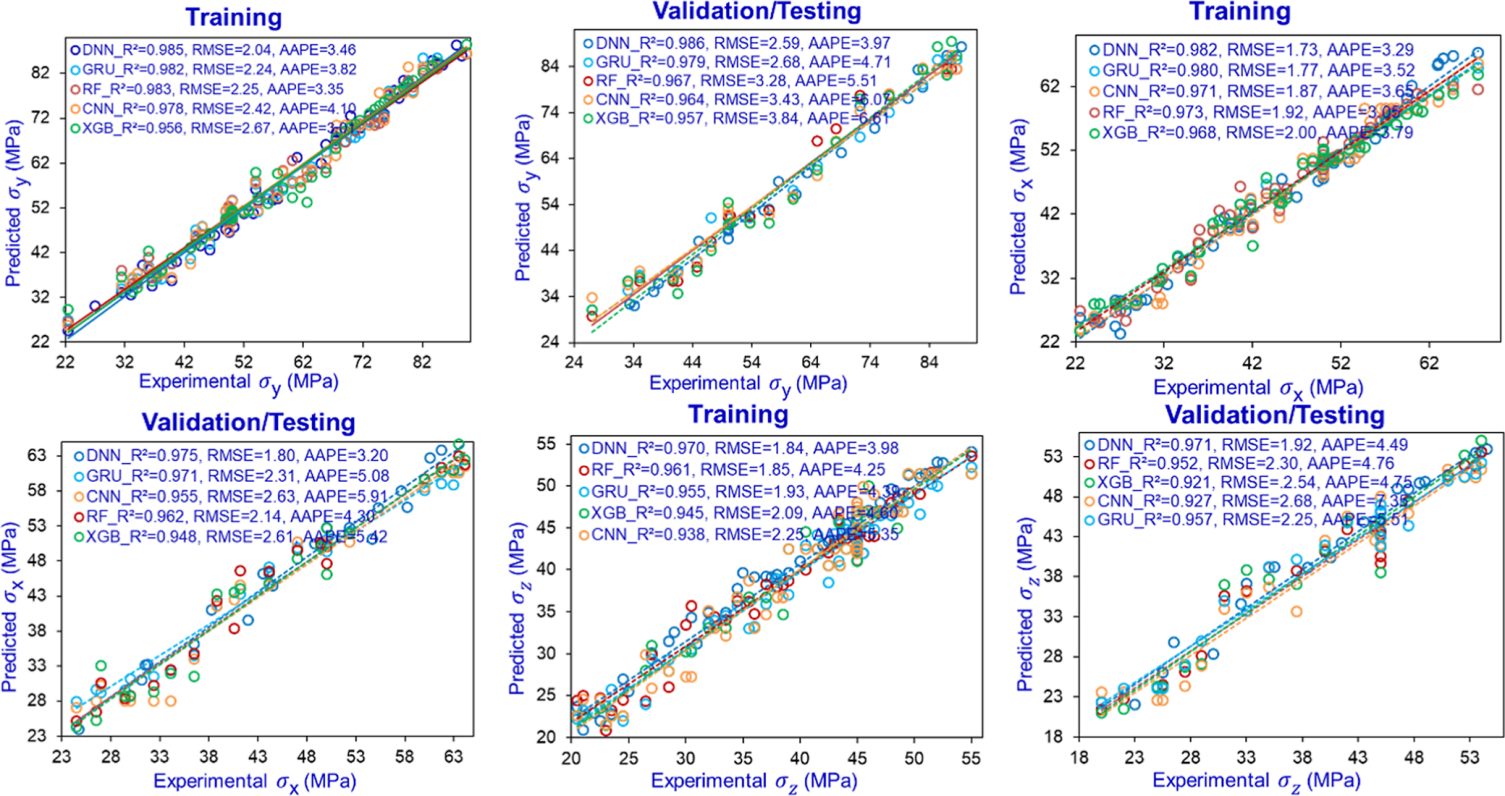
Cross plots between experimental and predicted values of stresses for the CNN, GRU, DNN, XGB, and RF models of σ_*z*_, σ_*y*_, and σ_*x*_ for core A.

**Figure 7:**
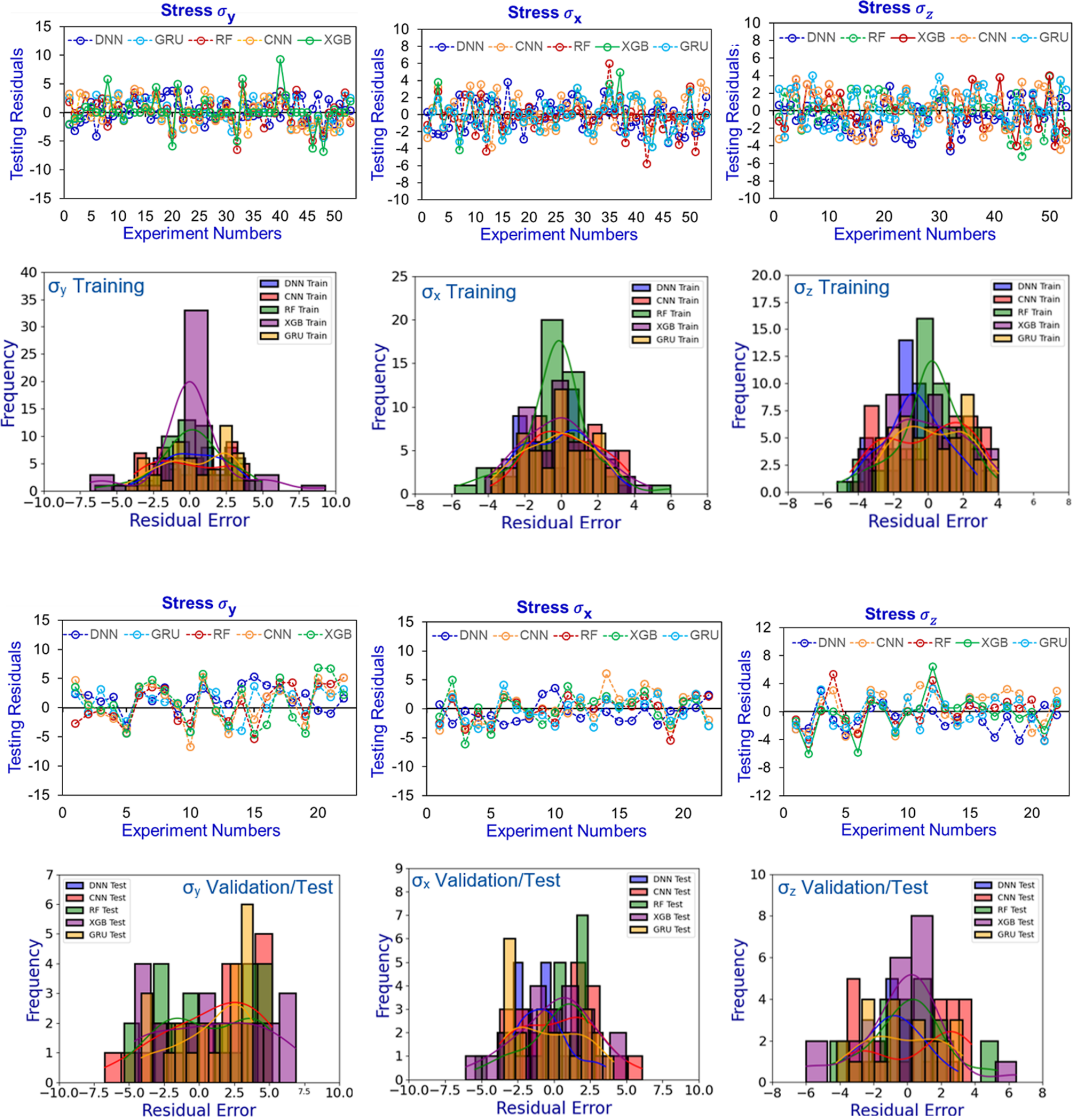
Training and validation residual errors of the predicted stresses σ_*x*_, σ_*y*_, and σ_*z*_ by CNN, GRU, XGB, RF, and DNN models for core A.

The prediction accuracy of the other four predictive models including CNN, XGB, RF, and GRU was also observed to be promising on the validation/testing data set, demonstrating their generalization capacity to blind (unseen) data. However, their performance metrics were marginally lower than those of the DNN models. The XGB models exhibited relatively higher prediction errors, with RMSE values of 3.84, 2.54, and 2.61 MPa and AAPE values of 6.61%, 4.75%, and 5.42% for stresses σ_*x*_, σ_*y*_, and σ_*z*_, respectively. Overall, the predictive models showed robust and reliable performance across both testing/validation and training stages, with minor variations in accuracy. Table 5 presents the accuracy measures of the models for core A, while the metrics of the models for cores B and C are provided in Appendix M.

**Table 5:** Accuracy Metrics of the Testing/Validation and Training Stages for the Proposed CNN, GRU, DNN, XGB, and RF Models of σ_*x*_, σ_*y*_, and σ_*z*_ Stresses

			ML models				
target variable	accuracy metrics	data category	DNN	RF	CNN	GRU	XGB
σ_*y*_	RMSE(MPa)	training	2.04	2.25	2.42	2.24	2.67
		test. and val.	2.59	3.28	3.43	2.68	3.84
	AAPE(%)	training	3.46	3.35	4.10	3.82	3.01
		test. and val.	3.97	5.51	6.07	4.71	6.61
	*R* ^2^	training	0.985	0.983	0.978	0.982	0.956
		test. and val.	0.986	0.967	0.964	0.979	0.957
σ_*z*_	RMSE (MPa)	training	1.84	1.85	2.25	1.93	2.09
		test. and val.	1.92	2.30	2.68	2.25	2.54
	AAPE (%)	training	3.98	4.25	5.35	4.39	4.60
		test. and val.	4.49	4.76	7.35	5.51	4.75
	*R* ^2^	training	0.970	0.961	0.938	0.955	0.945
		test. and val.	0.971	0.952	0.927	0.957	0.921
σ_*x*_	RMSE (MPa)	training	1.73	1.92	1.87	1.77	2.00
		test/val.	1.80	2.14	2.63	2.31	2.61
	AAPE (%)	training	3.29	3.05	3.65	3.52	3.79
		test/val.	3.20	4.30	5.91	5.08	5.42
	*R* ^2^	training	0.982	0.973	0.971	0.980	0.968
		test/val.	0.975	0.962	0.955	0.971	0.948

The generalization ability of the developed DNN models was assessed by using sensitivity or parametric analysis. The parametric and sensitivity study highlights the importance of input features in relation to the target output. A synthetic set of data was utilized to analyze the impact of each of the input features on the target features that illustrated the governing physics of the predictive models. In the synthetic data set, only one feature is varied while keeping all the inputs constant. Separate data sets were generated for each feature accordingly, and predictions were made using these data sets to examine their influence on the target variable, thereby revealing the physical relationships captured by the models. Further discussion and results of the sensitivity study of the DNN predictive models are provided in Appendix N.

### 4.2 Explainability of DNN Models—Local and Global Interpretation

In this scientific implementation, one of the primary emphases is on improving the explainability of the constructed DNN models, which elaborates the measure of human understanding about the reasoning and decision-making mechanism of complicated predictive models. Although predictive models provide outstanding predictive results, their inherent black-box nature limits transparency and interpretability. To address this issue, Shapley additive explanations (SHAP) analysis was utilized for explaining both local and global behaviors of the models by elucidating the inner mechanism of obtaining particular predictions. A Shapley value is allocated to every single input feature to obtain a particular prediction, providing a quantitative value of the average impact of the feature on the target output. Accordingly, various SHAP visualizations were employed in this work to improve the understanding of how individual input features influence the predictions across various instances.

The summary plots of SHAP values offer a global perception by highlighting the influence of input features on target prediction, relative to the model’s mean output, through integration of feature effects and significance as shown in Figure 8A. Every dot (point) corresponding to the feature’s Shapley value represents a single instance or data point. Input features are ranked based on their overall importance, enabling interpretation of how feature values are associated with their impact on target predictions. For instance, in the case of *V*_*zy*_ and *V*_*zx*_, higher input values are linked with lower SHAP values, indicating that larger values of these features tend to reduce the predicted stress compared to the model’s average output. Additionally, SHAP analysis facilitates understanding of interfeature relationships. The summary SHAP plots for the σ_*x*_ and σ_*z*_ models are illustrated in Appendix O. Furthermore, the mean SHAP plot (Figure 8B) demonstrates the mean values of SHAP magnitudes across all instances, illustrating the overall contribution of each input feature to the target predictions.

**Figure 8:**
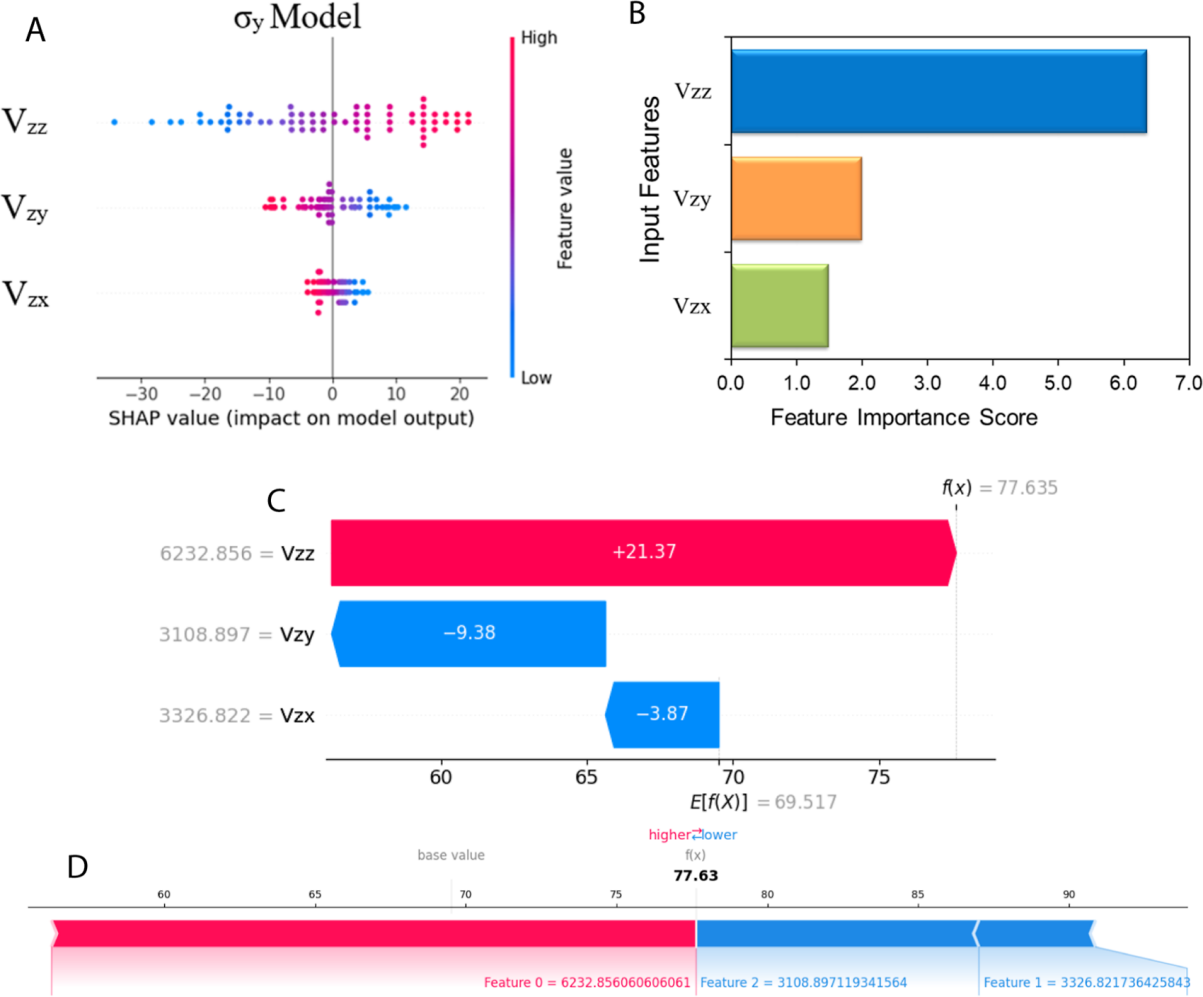
SHAP analysis of the model for σ_*y*_; (A) summary SHAP plots, (B) scores of feature importance; (C) waterfall plot for a particular data point illustrating the influence of features on stress prediction; (D) SHAP force plot illustrating the contribution of individual features to stress prediction.

The SHAP waterfall graph offers a visual illustration of the SHAP values reflecting the extent of influence of individual features on model predictions relative to the mean predicted output, thereby either reducing or amplifying the predicted stress values (σ_*y*_, σ_*z*_, and σ_*x*_) for each data point. The waterfall graph of the σ_*y*_ model is shown in Figure 8C, and the σ_*x*_ and σ_*z*_ model plots are demonstrated in Appendix O. The base values at the bottom of each plot show the average predicted values of stress for all data points, starting at *E*[*f*(*x*)] = 69.5, 45.7, and 31.1 MPa for σ_*y*_, σ_*x*_, and σ_*z*_, respectively. Each feature’s SHAP value demonstrates either a positive (red color) or negative (blue color) effect in moving the base value (anticipated model output) toward the final predicted value of stress for one instance. The predicted values for σ_*y*_, σ_*z*_, and σ_*x*_ within these given specific instances are 77.635, 35.17, and 51.597 MPa, respectively. For these specific instances, *V*_*zz*_ positively contributes to the σ_*y*_, σ_*z*_, and σ_*x*_ predictions by amplifying these stresses by 21.37, 4.42, and 10.78 MPa, respectively. The feature *V*_*zx*_ contributes negatively to the σ_*y*_, σ_*z*_, and σ_*x*_ predictions by reducing these stresses by 3.87, 11.94, and 5.92 MPa, respectively. On the contrary, the feature *V*_*zy*_ contributes positively to the σ_*z*_ by boosting the stress value by 9.07 MPa, while it has a negative contribution toward σ_*y*_ and σ_*x*_ predictions by reducing the stress values by 9.38 and 1.18 MPa, respectively. The SHAP waterfall plot findings align with the SHAP summary plots.

The SHAP force plot is employed for illustrating the SHAP values for individual instances or data points, offering insights similar to the waterfall plot. The force plot demonstrates how each input feature contributes to either increasing or decreasing the predicted stress values, ultimately leading to the final model outputs of 51.597, 35.17, and 77.635 MPa for σ_*x*_, σ_*z*_, and σ_*y*_, respectively. The force plot for the σ_*y*_ model is shown in Figure 8D, while the force plots of σ_*x*_ and σ_*z*_ are provided in Appendix O.

SHAP decision plots were also generated to gain insight into the prediction process of the DNN model for stress σ_*y*_, as illustrated in Figures 9A and 8B. The base value of the model is indicated by a point of line termination at the lower end of the graph, and every single line represents an individual data instance. The trajectory of each line reflects the impact of the SHAP values of the features to achieve the final predicted stress value at the top of the plot. Importantly, the outcomes from the decision plots for individual instances are consistent with those observed in the SHAP force, waterfall, and summary plots. Decision plots for the σ_*x*_ and σ_*z*_ models are presented in Appendix O.

**Figure 9:**
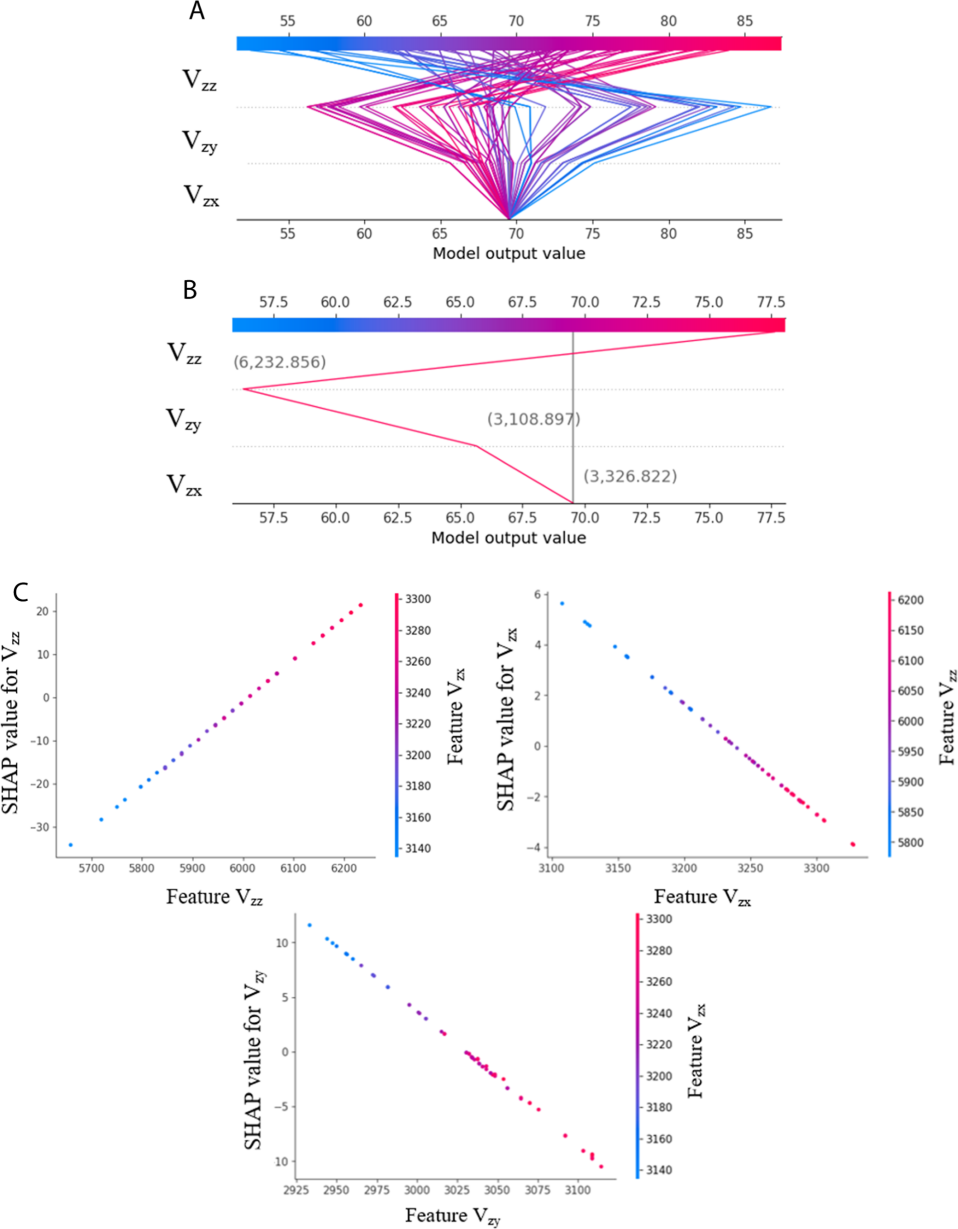
Dependence and decision plots of the stress σ_*y*_ model; (A) decision plot of several data points, (B) decision plot of a particular data point, and (C) three dependence graphs for input features *V*_*zy*_, *V*_*zx*_, and *V*_*zz*_.

SHAP dependence plots were also analyzed to better understand the influence of individual features on target predictions together with their interactions with other input features. Figure 9C presents the relationship between the input features (*V*_*zx*_, *V*_*zy*_, and *V*_*zz*_) and corresponding SHAP values and their impact on stress (σ_*y*_) prediction. The dependence plots for the other two stress models (σ_*x*_ and σ_*z*_) are included in Appendix O. The plot clearly indicates that higher values of *V*_*zz*_ are associated with positive SHAP values, leading to increased σ_*y*_ predictions, and vice versa. In contrast, higher values of *V*_*zy*_ and *V*_*zx*_ correspond to negative SHAP values, thereby contributing to lower predicted σ_*y*_ stress. Additionally, the interacting features show alternating trends, both decreasing and increasing, particularly in the areas around the center of the feature values (Figure 9C).

### 4.3 In Situ Stress Estimation in Well 16B(78)-32—Field Implementation and Scalability

In this phase, first, an unsupervised algorithm, namely, K-means clustering, was applied to categorize the subterranean rocks into distinct rock classes or petrofacies, adopting a similar methodology outlined by ref 41. The detailed illustrations about the petrofacies classification and analysis are provided in Appendix P. Then, the DNN models, trained on the modified TUV data set, were utilized to estimate in situ stresses in the near-field region within the subsurface geological rocks, based on sonic logging velocities obtained from the interval of measured depth ranges from 4835 to 10,872 feet in well 16B(78)-32. It is critical to highlight that the ML/DL models were trained using Biot–Gassmann-derived equivalent saturated velocities rather than ultrasonic dry velocities, as dry velocities are not the true representative of the field conditions, thus leading to the discrepancy in the ultrasonic dry velocities and field sonic logging velocities. Further, it is worth mentioning that the constructed DNN models corresponding to the principal stresses, maximum horizontal (σ_*x*_), minimum horizontal (σ_*z*_), and (vertical (σ_*y*_) stresses, were implemented for predicting the respective in situ stress components, namely, maximum horizontal (Sh_max_), minimum horizontal (Sh_min_), and vertical (S_v_) in the subsurface rocks. The input data, used in the relevant predictions, such as bulk density gradient, *V*_s‑fast_, *V*_s‑slow_, and *V*_p_, were obtained from log measurements along diversely oriented segments of well 16B(78)-32.

The predictive models of principal stresses were employed in accordance with particular stress orientations with respect to the wellbore axis: the normal stress along the wellbore trajectory or axis (σ_*y*_^′^) and two other orthogonally oriented principal stresses in the plane normal to the well axis (σ_*x*_^′^and σ_*y*_^′^). As illustrated in our previous study,^42^ the well comprises both vertical and deviated sections. Therefore, 3D stress transformation was applied to the deviated section to align the stress orientations with global principal stresses. Further details about well sections and stress transformation can be found in ref 42

Note that well logs consisting of sonic and density measurements were obtained from the proximity of the wellbore, where near-wellbore stress concentrations^53^ and thermo-poro-elastic stress disturbances^54,55^ may be anticipated. Thus, DNN predictions of in situ stresses can be affected because of near-wellbore stress changes, thus necessitating transformation to far-field in situ stresses using coupled thermo-poro-mechanical simulations.^53^

It is critical to discuss the implementation and scalability of laboratory-based measurements to the field data set. The laboratory-based TUV experiments, although conducted on small core samples, are designed to capture the critical velocity–stress relationship. The velocity–stress relationship dictates how the rock’s acoustic velocity (specifically, the Biot–Gassmann-based equivalent low-frequency saturated velocity) changes as a function of the three principal stresses. Then, ML models, essentially nonlinear functions, mathematically encapsulate this complex velocity–stress constitutive relationship. These ML models are the key to scaling up. Once trained and validated, this nonlinear function is ready to be applied to any measurement of P-wave and S-wave velocity acquired from the field.

The actual in situ stress prediction is performed using field sonic logging data acquired from the same wellbore. Then, the Biot–Gassmann fluid substitution method is applied to obtain the equivalent low-frequency saturated velocities using the properties of fluids encountered in the same area and depth location. This method of fluid substitution further reduces the uncertainty in the P- and S-wave velocities. Moreover, the continuous sonic logs are then fed, facies-by-facies, into the trained ML/DL models. The models that learned the unique stress–velocity behavior from each core sample directly output the corresponding three principal stresses (vertical, minimum horizontal, and maximum horizontal stresses).

### 4.4 Comparative Analysis

A comparative analysis was performed between results obtained from this study and the previous study that used laboratory experiments performed on saturated rocks.^42^ First, the Biot–Gassmann-derived low-frequency equivalent saturated acoustic velocities were compared with the ultrasonic saturated velocities. The results revealed an observable difference between the P-wave velocities, ranging from 1.5 to 2% approximately, obtained from the ultrasonic measurements and the Biot–Gassmann method, as illustrated in Figure 10. The difference in the P-waves may be attributed to velocity dispersion phenomena in the low-porosity (∼2%) gneiss, granite, and quartz gneiss core samples. Wave propagation at ultrasonic frequencies (1 MHz) may result in the squirt flow, where fluid is squeezed between interconnected pores as the wave propagation induces local fluid pressure gradients within the pore spaces and microcracks. This can cause the bulk modulus to increase and thus increase the P-wave velocities beyond the quasi-static condition. In contrast, the Biot–Gassmann method assumes a quasi-static condition (low-frequency regime) where pressure equilibration occurs, resulting in a lower velocity without high-frequency stiffening effects. This dispersion effect is more pronounced in P-wave velocities due to their sensitivity to fluid bulk modulus, while S-wave velocities show a smaller deviation, approximately 0.2–0.4%, as shear waves are less affected by fluid content and primarily influenced by the slight density increase from fluid saturation. However, crystalline rocks like gneiss, granite, and quartz-gneiss, investigated in this work, demonstrated low magnitudes of dispersion due to their low porosities (∼2%), aligning with the modest 1.5–2% difference observed in the P-wave velocities, which is consistent with the intrinsic properties of these dense metamorphic and igneous rocks. This finding aligns with reported studies on velocity dispersion in low-porosity metamorphic rocks (quartzite) and igneous (granite) rocks, where ultrasonic saturated P-wave velocities exceed Biot–Gassmann low-frequency approximation by similar relative differences (1–3%) caused by the enhanced stiffness from the squirt flow.^25,56–58^ Whether this difference is important is application-specific. We next compare the impact on the stress predictions obtained from models trained on Biot–Gassmann-derived equivalent saturated velocities and ultrasonic saturated velocities.

**Figure 10:**
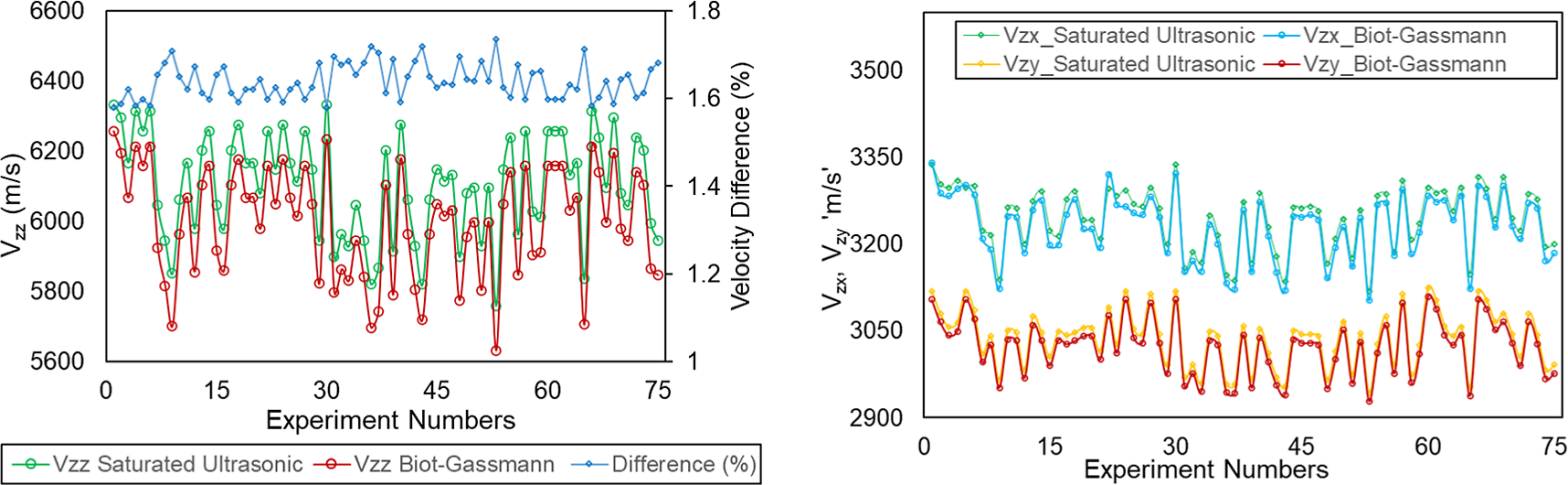
Comparison of Biot–Gassmann-derived equivalent saturated acoustic velocities with measured ultrasonic saturated velocities.

Like a previous study,^42^ this work has also demonstrated a good agreement between the in situ stresses (Sh_min_ and S_v_) obtained from DNN predictions and the field-based elastic geomechanical model (FB-EGM), particularly for the representative petrofacies (PF-6, PF-5, and PF-1). The representative petrofacies (PF) are descriptive of the constitutive elastic behavior of subsurface core (used for the TUV experiment) samples.

The cross plots obtained from this work were compared with the previous study by ref 42, as illustrated in Figure 11. Although both cases reflected excellent generalization capabilities of DNN models for the representative petrofacies (PF-6, -5, and -1). However, DNN models trained using Biot–Gassmann-derived equivalent saturated acoustic velocities demonstrated improved predictive performance for in situ stresses compared to our previous study^42^ in which DNN models were trained on experimentally measured ultrasonic velocities on saturated rocks. Specifically, the cross-plots for representative petrofacies (PF-1, -5, and -6) showed higher *R* ^2^ values in the current study, i.e., 0.843 and 0.91 for Sh_min_ and *S*_v_, respectively, compared to the previous study^42^ (0.813 and 0.865 for Sh_min_ and *S*_v_, respectively). Of particular note is the performance of the model for predicting *S*_v_, owing to the fact that *S*_v_ can be reasonably well-predicted by integrating the density log, and so it comprises a validation case. This is in contrast to the elastic geomechanical model (EGM) for the minimum horizontal stress, which is prone to uncertainty stemming from poor constraints on the tectonic strain, impact of creep, and so forth. This study shows a much tighter grouping of predictions around the unity line for *S*_v_ compared with the previous study that used laboratory experiments on saturated rocks.

**Figure 11:**
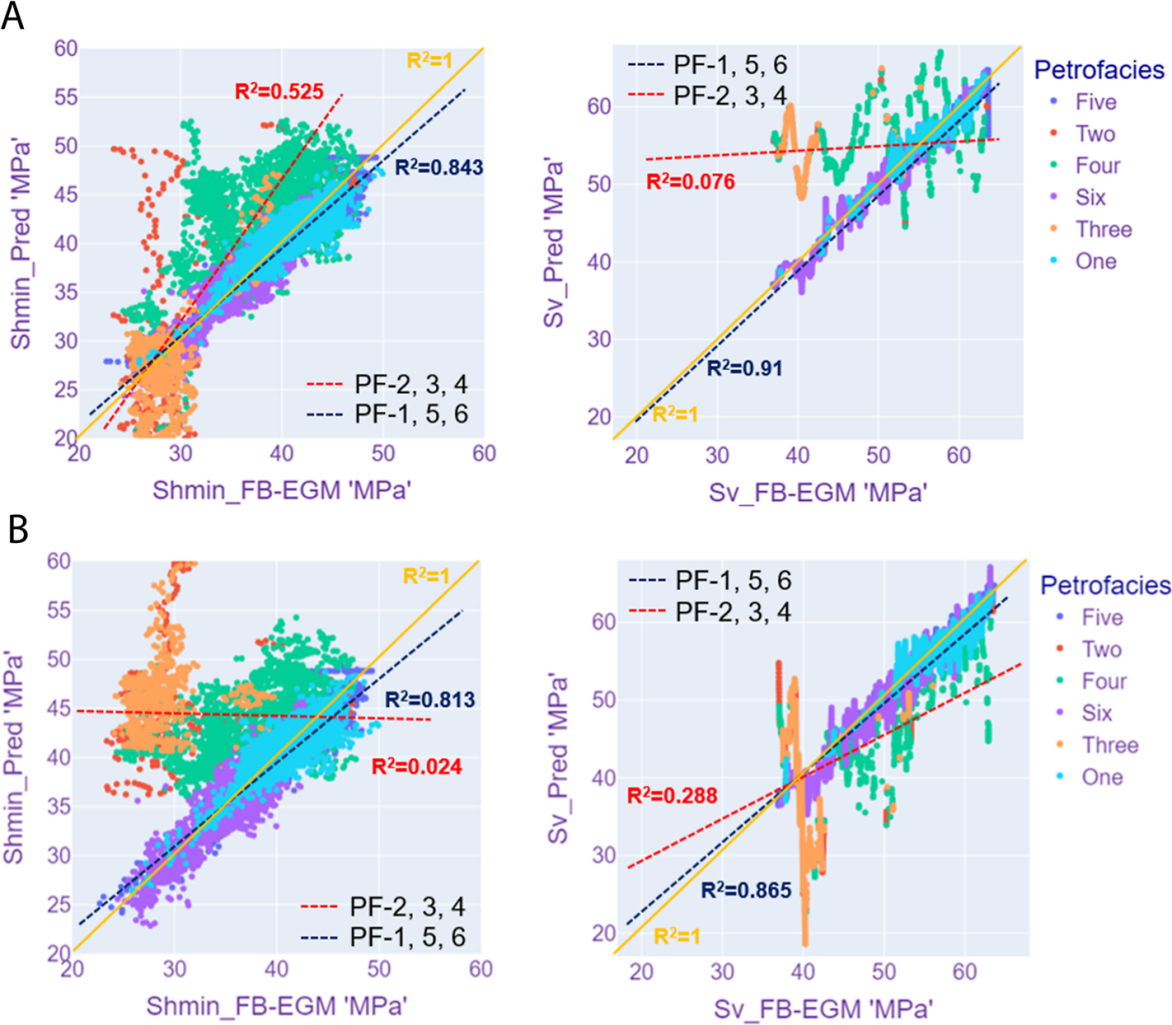
Cross plot between the FB-EGM- and DNN-predicted Sh_min_ (left) and *S*_v_ (right) for representative and nonrepresentative PFs: (A) this study; (B) Mustafa et al.^42^

This improved accuracy of in situ stress prediction indicated that measurements of elastic properties under quasi-static conditions (at low frequencies) better imitate the subsurface reservoir characteristics compared to the elastic properties measured at ultrasonic frequencies. The frequency of the equivalent saturated acoustic velocities (derived from Biot–Gassmann) has better alignment with field-scale sonic logging data. This is understood to stem from the fact that laboratory TUV measurements are acquired at ultrasonic frequencies (1 MHz), whereas field sonic logs operate at lower frequencies (10–20 kHz). Second, the ultrasonic velocities measured directly on saturated core samples may include dispersion effects (velocity increase due to squirt flow or local fluid pressure gradients). This subtle discrepancy between high-frequency laboratory data and low-frequency field conditions could result in slightly different in situ stresses. The Biot–Gassmann method mitigates dispersion effects by providing a low-frequency saturated equivalent of dry core velocities, therefore leading to enhanced generalization of the models to field conditions. Thus, predicted principal in situ stresses in the well 16B(78)-32 evidenced slightly higher *R* ^2^ values of 0.91 and 0.843 for vertical stresses and minimum horizontal stresses, respectively, possibly due to the harmonization of frequency domains.

Moreover, comparisons between the FB-EGM- and DNN-predicted principal stresses for this work and a previously reported study^42^ are presented in Figure 12. In both cases, FB-EGM- and DNN-based principal stresses are in good harmony, indicating model generalization capabilities. However, in situ stresses obtained in this work more closely resemble the FB-EGM-based in situ stresses with less fluctuation compared to a previous study.^42^ In this work, achievement of improved accuracy of predicted in situ stresses indicated that the velocity–stress relationship captured at low-frequency conditions emulates the constitutive behavior of subsurface rocks under more precise reservoir conditions, particularly for representative PF zones (PF-1, 5, 6). The findings of this work demonstrate that the stress–velocity constitutive relationship can be more effective if the frequency of measured velocities matches the frequency range of sonic logging measurements.

**Figure 12:**
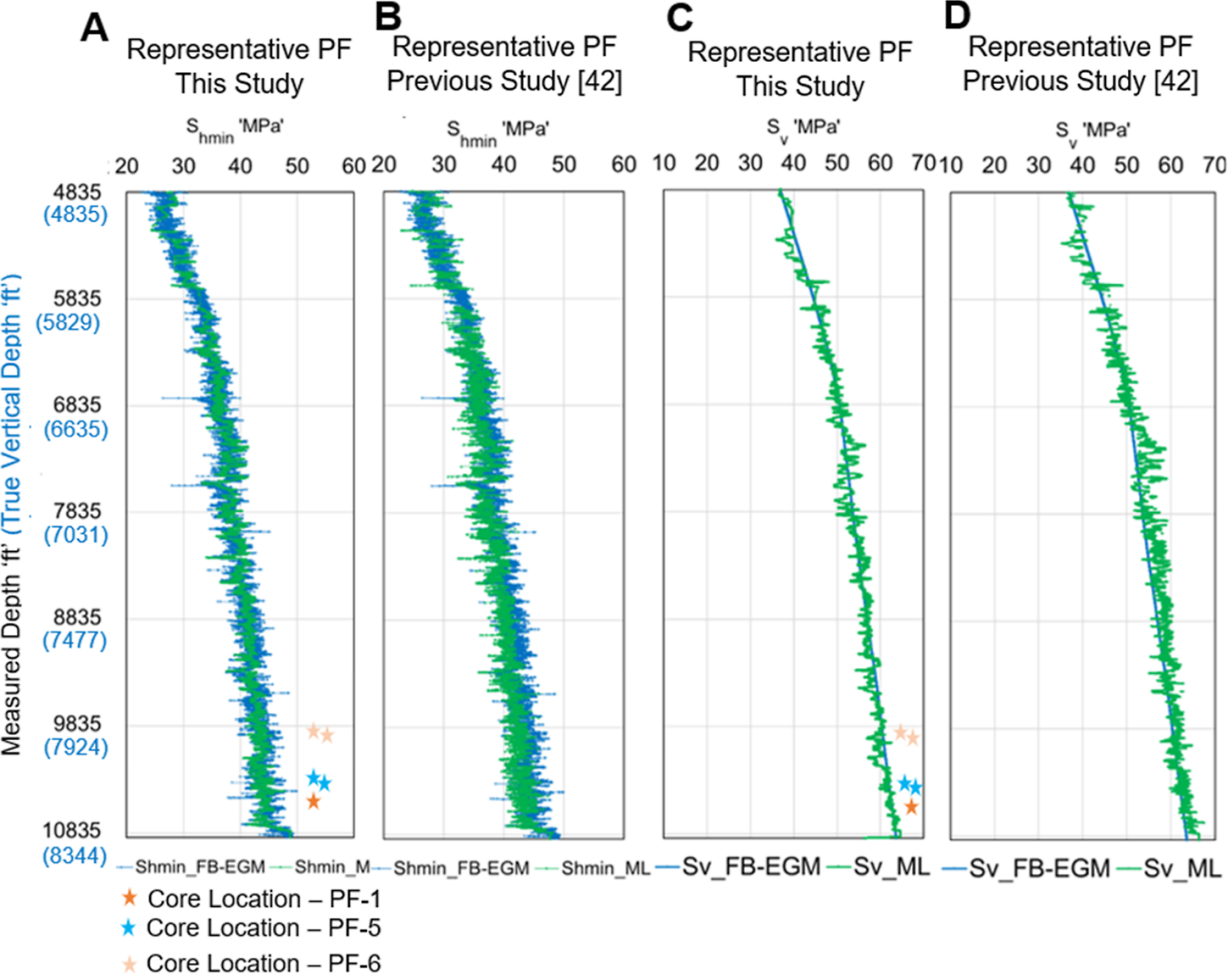
A comparison between DNN-predicted and FB-EGM-based in situ stresses in well 16B(78)-32; (A) comparing the Sh_min_ for representative PF, results of this study; (B) comparing the Sh_min_ for the representative PF, reported in Mustafa et al.;^42^ (C) comparing *S*_v_ for representative PF, results of this study; (D) comparing *S*_v_ for representative PF, reported in Mustafa et al.^42^ Stars represent the locations/depths of core samples of different PFs.

In this work, the improved prediction performance offers a more realistic, low-frequency approximation of acoustic wave propagation in subsurface formations. This better aligns with the frequency range of field sonic logs used as inputs in stress prediction and reduces the mismatch between laboratory and field conditions. Hence, this approach preserves the facies-specific elastic behavior of the subsurface rocks, effectively replicating field conditions, thereby enhancing the robustness of DNN-based in situ stress predictions in subsurface geological formations. Additionally, the Biot–Gassmann method incorporates fluid and poroelastic effects analytically, enabling the transformation of clean dry-frame measurements into more realistic field-representative input features. This approach may also act as a mathematical smoothing process, reducing noise in the velocity–stress relationship and strengthening the facies-specific elastic behavior captured in the DNN models. Consequently, the DNN models trained using the Biot–Gassmann-based saturated velocities provided more robust and accurate stress predictions across the representative petrofacies in the geothermal well.

Although this workflow provides an efficient approach for estimating in situ principal stresses by discovering and applying stress–velocity relationships, it has certain limitations. First, the velocity–stress constitutive relation, captured by ML/DL models, is primarily valid for representative petrofacies (PFs) that exhibit petrophysical and lithological features analogous to core sampling locations. Consequently, to expand the applicability of these models to other PFs identified in the same well, it is essential to acquire subsurface core samples from at least one zone or layer of each PF. Furthermore, principal in situ stresses may only be estimated accurately if the orientation of subsurface cores is known. Additionally, DL/ML models should be trained on the TUV data set that is acquired from unfractured/intact cores. Hence, the in situ stress magnitudes predicted by the models will be uncertain in regions with high fracture intensity or fault shear zones where local stress changes might occur. Despite these limitations, this study provides a reliable and consistent estimate of the in situ stresses in subterranean rocks under suitable conditions. Thus, this study offers an economical and time-efficient solution to obtain in situ principal stresses compared to conventional approaches and expensive field-based fluid injection tests such as microfracturing or mini-frac tests.

## 5 Conclusions

Accurate determination of in situ principal stresses in subsurface rock formations remains a major challenge due to inherent uncertainties and limitations of existing methods. For this challenge, an ML/DL-based workflow has previously been demonstrated where the model is trained on laboratory triaxial ultrasonic velocity measurements on saturated rocks.^42^ Here, we show that using dry rocks with the Biot–Gassmann correction gives a somewhat better result. Moreover, these experiments can be carried out without time-consuming saturation steps and at high temperatures without concern for inducing a phase change in the pore fluid. Moreover, explainability and transparency of complicated DL predictive models are critical for scientific applications and for improving human intuition of the decision-making mechanism of the models.

This research adopts the integrated framework outlined by Mustafa et al.^42^ to train the ML/DL models using low-frequency Biot–Gassmann-derived velocities and implement the models for accurately estimating in situ principal stresses in subsurface rocks of a geothermal well. Comparative analysis revealed that the superior and generalized prediction results were obtained from DNN models with RMSE values of 2.59, 1.92, and 1.80 for validation/testing predictions of the principal stress (σ_*x*_, σ_*y*_, and σ_*z*_) models. Further, analysis revealed the dispersion effect of frequency on the velocity–stress relationship and hence on in situ stress prediction. Thus, this leads to a small improvement in predicted in situ stresses using low-frequency Biot–Gassmann-derived saturated velocities compared to ultrasonic saturated velocities^42^ due to better frequency alignment with field measurements. A parametric study revealed the good generalization capacity of the DNN models. Further, critical insights and reasoning behind the complex process of DNN predictive models were elaborated by SHAP analysis, explaining the interaction and impact of input features on the ultimate prediction of models.

The DNN models, trained on Biot–Gassmann-derived saturated acoustic velocities, demonstrated promising functionality on field-based sonic logs for estimating in situ principal stresses in the zones of representative PF in well 16B(78)-32. Thus, the proposed ML/DL workflow with the modified TUV data set (using Biot–Gassmann equivalent saturated acoustic velocities) delivers an economical and time-efficient alternative to traditional methods of estimating in situ stress. Further, reliable predicted stresses can be beneficial in planning and designing subsurface activities, such as completions, drilling, hydraulic fracturing stimulations, subsurface mining, and tunnel excavation.

## Supplementary Material



## Notes

Appendices A–P and table of contents (TOC) graphic (PDF)

## #

Now at Civil, Environmental, and Geo-Engineering Department, University of Minnesota, MN, USA

## References

[1] Pinilla, D. ; Fulton, P. ; Jordan, T. Preliminary determination of in-situ stress orientation and magnitude at the Cornell University Borehole Observatory (CUBO) geothermal well, Ithaca NY. InProceedings, 48th Workshop on Geothermal Reservoir Engineering Stanford University, Stanford, CA, USA, 6–8 Feb 2023. (SGP-TR-224), 2023.

[2] Li X., Chen S., Wang S., Zhao M., Liu H. (2021). Study on in
situ stress distribution law of the deep mine: taking Linyi mining
area as an example. Adv. Mater. Sci. Eng..

[3] Cao L., Yao Y., Cui C., Sun Q. (2020). Characteristics of
in-situ stress
and its controls on coalbed methane development in the southeastern
Qinshui Basin, North China. Energy Geosci..

[4] Mortimer L., Aydin A., Simmons C. T., Love A. J. (2011). Is in situ stress
important to groundwater flow in shallow fractured rock aquifers?. J. Hydrol..

[5] He B. G., Li H. P., Feng X. T., Meng X. R. (2024). Estimating
the complete
in-situ stress tensor along deep tunnels with frequent rockbursts
near a steep valley. Bull. Eng. Geol. Environ..

[6] Kruszewski M., Montegrossi G., Parisio F., Saenger E. H. (2022). Borehole observation-based
in situ stress state estimation of the Los Humeros geothermal field
(Mexico). Geomech. Energy Environ..

[7] Ibrahim, M. M. , Ibrahim, A. F. , Ibrahim, M. , Pieprzica, C. ; Ozkan, E. Determination of ISIP of non-ideal behavior during diagnostic fracture injection tests. Proceeding, SPE Annual Technical Conference and Exhibition, Calgary, Canada, 30 Sept-02 Oct 2019. SPE, 2019.

[8] Nolte K. G. (1988). Principles
for fracture design based on pressure analysis. SPE Prod. Eng..

[9] Chang C., Jo Y., Oh Y., Lee T. J., Kim K. Y. (2014). Hydraulic Fracturing
In-Situ Stress Estimations in a Potential Geothermal Site, Seokmo
Island, South Korea. Rock Mech. Rock Eng..

[10] Zang A., Stephansson O., Heidbach O., Janouschkowetz S. (2012). World stress
map database as a resource for rock mechanics and rock engineering. Geotech. Geol. Eng..

[11] Fjaer, E. ; Holt, R. M. ; Raaen, A. ; Horsrud, P. Petroleum Related Rock Mechanics, 2 ed.; Elsevier, 2008.

[12] Gholami R., Rasouli V., Aadnoy B., Mohammadi R. (2015). Application
of in situ stress estimation methods in wellbore stability analysis
under isotropic and anisotropic conditions. J. Geophys. Eng..

[13] Sinha A., Joshi Y. K. (2011). Downhole electronics cooling using
a thermoelectric
device and heat exchanger arrangement. J. Electron.
Packag..

[14] Lu, G. ; Lu, Y. ; Kelley, M. ; Raziperchikolaee, S. ; Bunger, A. Interpretation of Mini-frac Test Data Accounting for Wellbore Cooldown in an EGS Well at the Utah FORGE Site: A Numerical Study. 2023 Geothermal Rising Conference, Reno, 1–5 October 2023. GRC Transactions 2023, vol-47, p 2023.

[15] Lu G., Kelley M., Raziperchikolaee S., Bunger A. P. (2024). Modeling the impact
of thermal stresses induced by wellbore cooldown on the breakdown
pressure and geometry of a hydraulic fracture from an EGS well. Rock Mech. Rock Eng..

[16] Mahmoud, A. A. ; Elkatatny, A. ; Abdulraheem, A. Machine learning applications in the petroleum industry. In Book Chapter, Handbook of Energy Transitions; CRC Press, 2022.

[17] Shan F., He X., Xu H., Armaghani D. J., Sheng D. (2023). Applications of Machine
Learning in Mechanized Tunnel Construction: A Systematic Review. Eng..

[18] Zhang W., Gu X., Hong L., Han L., Wang L. (2023). Comprehensive review
of machine learning in geotechnical reliability analysis: Algorithms,
applications and further challenges. Appl. Soft
Comput..

[19] Haggerty R., Sun J., Yu H., Li Y. (2023). Application of machine learning in
groundwater quality modelling - A comprehensive review. Water Res..

[20] Jung D., Choi Y. (2021). Systematic Review of
Machine Learning Applications in Mining: Exploration,
Exploitation, and Reclamation. Minerals.

[21] Dargahi-Zarandi A., Hemmati-Sarapardeh A., Shateri M., Menad N. A., Ahmadi M. (2020). Modeling minimum
miscibility pressure of pure/impure CO 2 -crude oil systems using
adaptive boosting support vector regression: Application to gas injection
processes. J. Pet. Sci. Eng..

[22] Hassanvand M., Moradi S., Fattahi M., Zargar G., Kamari M. (2018). Estimation
of rock uniaxial compressive strength for an Iranian carbonate oil
reservoir: Modeling vs. artificial neural network application. Pet. Res..

[23] Mahmoud A. A., Elkatatny S., Al-Abduljabbar A. (2021). Application
of machine learning models
for real-time prediction of the formation lithology and tops from
the drilling parameters. J. Pet. Sci. Eng..

[24] Serfidan A. C., Uzman F., Türkay M. (2020). Optimal estimation
of physical properties
of the products of an atmospheric distillation column using support
vector regression. Comput. Chem. Eng..

[25] Mavko, G. , Mukerji, T. ; Dvorkin, J. The Rock Physics Handbook (2nd ed.). Cambridge University Press, 2009.

[26] Gurevich B., Mavko G., Zhukov A. (2009). Velocity dispersion and attenuation
in rock with two scales of porosity. Geophysics.

[27] Dvorkin J., Nur A. (1992). Squirt flow in fully
saturated rocks. Geophysics.

[28] Mavko G., Jizba D. (1991). Estimating seismic
velocity dispersion from rock properties. Geophysics.

[29] Gassmann F. (1951). Über
die Elastizität poröser Medien. Vierteljahrsschr. Naturforsch. Ges. Zuerich.

[30] Biot M. A. (1956). Theory
of propagation of elastic waves in a fluid-saturated porous solid.
I. Low-frequency range. J. Acoust. Soc. Am..

[31] Berryman J. G. (1999). Origin
of Gassmann’s equations. Geophysics.

[32] Wang, Z. Z. Fundamentals of seismic rock physics, 2010.

[33] Lee, M. W. Velocity ratio and its application to predicting velocities; US Department of the Interior, US Geological Survey: Reston, VA, USA, 2003.

[34] Song Y., Hu H., Rudnicki J. W. (2016). Deriving
Biot-Gassmann relationship by inclusion-based
method. Geophysics.

[35] Lee M. W. (2002). Biot–Gassmann
theory for velocities of gas hydrate-bearing sediments. Geophysics.

[36] Ali S., Akhlaq F., Imran A. S., Kastrati Z., Daudpota S. M., Moosa M. (2023). The enlightening role of explainable artificial intelligence in medical
& healthcare domains: A systematic literature review. Comput. Biol. Med..

[37] Lundberg, S. M. ; Lee, S.-I. A unified approach to interpreting model predictions. Adv. Neural Inf. Process. Syst. 2017, 30.

[38] Ding X., Hasanipanah M., Rezaei M. (2025). Assessment of mechanical properties
of rock using deep learning approaches. Measurement.

[39] Sun D., Chen D., Zhang J., Mi C., Gu Q., Wen H. (2023). Landslide susceptibility mapping
based on interpretable machine learning
from the perspective of geomorphological differentiation. Land.

[40] Yang S., Luo D., Tan J., Li S., Song X., Xiong R., Wang J., Ma C., Xiong H. (2024). Spatial mapping and
prediction of groundwater quality using ensemble learning models and
shapley additive explanations with spatial uncertainty analysis. Water.

[41] Mustafa A., Kelley M., Lu G., Bunger A. P. (2024). An Integrated Machine
Learning Workflow to Estimate In-Situ Stresses Based on Downhole Sonic
Logs and Laboratory Triaxial Ultrasonic Velocity Data. J. Geophys. Res. Mech. Learn. Comput..

[42] Mustafa A., Lu G., Bunger A. P. (2026). Evolution of learning
curve and white box machine learning
models for estimating in-situ stresses based on velocity-stress relationship. Fuel.

[43] Bunger A. P., Higgins J., Huang Y., Hartz O., Kelley M. (2024). Integration
of Triaxial Ultrasonic Velocity and Deformation Rate Analysis for
Core-Based Estimation of Stresses at the Utah FORGE Geothermal Site. Geothermics.

[44] Smith T. M., Sondergeld C. H., Rai C. S. (2003). Gassmann fluid substitutions: A tutorial. Geophysics.

[45] Kumar, D. A tutorial on Gassmann fluid substitution: Formulation, algorithm and Matlab code. Matrix 2006, 2(1).

[46] Jones, C. Utah FORGE: Well 16B(78)-32 Cuttings X-Ray Diffraction Data. [Data set]. https://gdr.openei.org/submissions/1731; Geothermal Data Repository; Energy and Geoscience Institute at the University of Utah, 2025.

[47] Kirby, S. M. , Simmons, S. , Paul, C. I. , Stan, S. . Groundwater Hydrogeology and Geochemistry of the Utah FORGE Site and Vicinity, Utah Geological Survey Miscellaneous Publication 169-E, 2 plates, scale 1; 2019.

[48] Simmons, S. ; Kirby, S. ; Jones, C. ; Moore, J. ; Allis, R. ; Brandt, A. ; Nash, G. The geology, geochemistry, and geohydrology of the FORGE deep well site, Milford, Utah. 41st Workshop on Geothermal Reservoir Engineering 2016, Vol 2016, pp-1–10.

[49] Project Report Enhanced Geothermal System Testing and Development at the Milford, Utah FORGE. 2016, Retrieved on 08/05/2026. https://www.energy.gov/sites/prod/files/2016/09/f33/Conceptual_Geologic_Model_FORGE_Milford_UT.pdf.

[50] Rousseeuw P. J. (1987). Silhouettes:
A graphical aid to the interpretation and validation of cluster analysis. J. Comput. Appl. Math..

[51] Liashchynskyi P., Liashchynskyi P. (2019). Grid search,
random search, genetic algorithm: A big
comparison for NAS. arXiv.

[52] Belyadi, H. ; Haghighat, A. Machine learning guide for oil and gas using Python; Elsevier; Vol. 10; 2021.

[53] Kirsch E. G. (1998). Die Theorie
der Elastizitat und die Bedurfnisse der Festigkeitslehre. Zeitshrift des Vereines deutscher Ingenieure.

[54] Tao Q., Ghassemi A. (2010). Poro-thermoelastic
borehole stress analysis for determination
of the in-situ stress and rock strength. Geothermics.

[55] Lu, G. , Lu, Y. , Kelley, M. , Raziperchikolaee, S. , Bunger, A. P. Thermo-poro-elastic stress alteration around an EGS well due to cold fluid circulation. In 58th US Rock Mechanics/Geomechanics Symposium; ARMA; 2024.

[56] Schijns, H. M. . Experimental investigation of seismic velocity dispersion in cracked crystalline rock.2014.

[57] Murphy W., Winkler K. W., Kleinberg R. L. (1986). Acoustic
relaxation in sedimentary
rocks; dependence on grain contacts and fluid saturation. Geophysics.

[58] Prasad M., Manghnani M. H. (1997). Effects of pore and differential pressure on compressional
wave velocity and quality factor in Berea and Michigan sandstones. Geophysics.

