# Supplementary material for: Integration of the Biot–Gassmann Fluid Substitution Method and Machine Learning-Based Velocity–Stress Relationship for Estimating In Situ Stresses
Source: ACS Omega. 2026 Jan 28;11(5):7841–60. doi: 10.1021/acsomega.5c09669 (PMC12902857; doi:10.1021/acsomega.5c09669)
Supplement: Supplementary file 1 [file ao5c09669_si_001.pdf]

Integration of Biot-Gassmann Fluid Substitution Method and Machine Learning-based Velocity-Stress Relationship for Estimating In-situ Stresses

Authors: Ayyaz Mustafa[a], Guanyi Lu[b], Andrew P. Bunger[a,c*]

[a] Department of Civil and Environmental Engineering, University of Pittsburgh, PA, USA.

[b] Department of Civil and Environmental Engineering, University of Pittsburgh, PA, USA. Now at Civil, Environmental, and Geo-Engineering Department, University of Minnesota, MN, USA.

[c] Department of Chemical and Petroleum Engineering, University of Pittsburgh, PA, USA.

Corresponding Author: Andrew P. Bunger* (bunger@pitt.edu)

Authors Email Addresses: Ayyaz Mustafa: aym40@pitt.edu; Guanyi Lu: lug@umn.edu; Andrew P. Bunger: bunger@pitt.edu

Appendices

Appendix A: Details about the ML/DL algorithms used in this study.

A-1. Deep Neural Network (DNN)

The Deep Neural Network (DNN), a form of Artificial Neural Network (ANN) characterized by multiple hidden layers, has been widely employed in machine learning for diverse applications including function approximation, data mining, pattern recognition, and predictive modeling [1]. DNNs utilized various learning algorithms, activation functions, and network structures to solve complex engineering problems efficiently [2, 3]. By replicating the structure and operations of the human nervous system, DNNs effectively addressed problem-specific computational tasks. The network's foundational components (artificial neurons) were structured according to the specific requirements of each engineering application [4, 5]. Through a range of techniques, DNNs were able to capture intricate nonlinear relationships among input features, enabling the generation of highly accurate, stable, and robust predictive outputs [6].

Deep Neural Networks (DNNs) are primarily classified into two types: feedforward neural networks (FNNs) and feedback neural networks (FBNNs) [7, 8]. FNNs are networks that include multiple layers of interconnected perceptrons that propagate a signal in one direction, from the input layer toward the output layer, in the absence of feedback loops. This linear arrangement allows for a sequential modification of the input signal across multiple hidden layers using activation functions to extract complex features and provide accurate performance. In contrast, FBNNs, although structurally similar to FNNs, integrate a feedback mechanism into the network [9, 10]. This mechanism returns error signals back through the network, allowing iterative updates to the weights of the neurons. The error correction process continues until the model converges by minimizing the prediction error, thereby improving the overall accuracy of the model's output [11, 12].

A-2. Convolutional Neural Network (CNN)

Convolutional Neural Networks (CNNs) were originally developed for computer vision applications, particularly image and object recognition [13]. Their scope has broadened to encompass regression tasks, especially in contexts where identifying spatial or structural trends in the data is critical. CNN excel in processing data presented in grid forms and are applicable to structured numerical or tabular data when formatted appropriately [14]. A standard CNN architecture incorporates pooling, convolutional, and fully connected layers. The convolutional layers are mainly used for extracting spatial and hierarchical features by deploying filters (kernels) across the input data, permitting the detection of local patterns and their integration into higher-level representations. Pooling layers (max pooling) decrease the dimensionality of feature maps, boost computational efficiency while diminishing the likelihood of overfitting. The final phase, the fully connected layers, assimilates the extracted features for predictive tasks [14].

In regression applications, Convolutional Neural Networks (CNNs) employ convolutional layers to derive features that reflect intrinsic spatial correlations within the data. These extracted features are subsequently processed through fully connected layers to translate the high-dimensional representations into continuous output values [15]. This capability renders CNNs particularly effective for predicting physical parameters, analyzing time-series data structured as matrices, and estimating real-world quantities such as temperature or stress [16]. The hierarchical learning of spatial features by CNNs positions them as a powerful alternative to conventional machine learning approaches in regression tasks [17, 18].

A-3. Gated Recurrent Units (GRU)

Cho et al. (2014) [19] first proposed the Gated Recurrent Unit (GRU) that is a simpler version of the Recurrent Neural Network (RNN) and is considered as a more efficient version of the Long Short-Term Memory (LSTM) network in terms of computations and time. Like LSTM, the GRU was developed to solve the vanishing gradient problem experienced by the conventional RNN, thus making it suitable for time series forecasting and regression prediction. The operation of GRUs relies on two important gates: the update gate, which controls amount of information to be added to the current hidden state, and the reset gate, which

controls the amount of information to be forgotten. Unlike LSTM, GRUs do not utilize a separate memory cell, while the hidden and memory states are merged into one vector. This simplification in the architecture allows GRUs to have a high prediction accuracy with reduced computation, which is useful particularly in environments with limited resources [19] Using this two-gate mechanism improves the capability of the model to learn temporal dependencies in regression [20].

Gated Recurrent Units (GRUs) have achieved competitive results on regression tasks, particularly those with sequential or time-dependent data, as a consequence of their capability to effectively learn temporal dependencies and produce accurate real-valued predictions. In comparison to Long Short-Term Memory (LSTM) networks, GRUs are less complex and faster, which makes them an attractive option for problems involving simpler datasets or those with constraints on computational resources [21]. Their efficiency and forecasting performance have led to their widespread use in a various applications, including speech analysis, meteorological modeling, and sentiment analysis, where both short- and long-range dependencies need to be effectively modeled [20]. Owing to their capability to strike a compromise between model simplicity, computational efficiency, and predictive performance, GRUs have also become a widely used modeling strategy in current machine learning studies and real-world scenarios [22].

A-4. Random Forest (RF)

Random Forest (RF) is a robust supervised machine learning technique that employs an ensemble learning method to carry out classification and regression tasks. Ensemble learning combines the predictions of several base models to yield more robust and accurate predictions than individual base learners. In RF, this is achieved by constructing a set of decision trees, each of which is trained on a bootstrap sample of the actual dataset, with a randomly selected subset of variables considered at each split in the tree building process. The final prediction is obtained by averaging the predictions (in regression) or using majority voting (in classification) among all the individual trees. This ensemble approach significantly improves the model's generalization ability and stability, making RF an effective tool across a wide range of predictive modeling tasks [23]. Further methodological insights are available in the original work by Svetnik et al., [24].

A-5. Extreme Gradient Boosting (XGB)

Extreme Gradient Boosting (XGB) is a supervised ensemble machine learning algorithm that may be utilized for both classification and regression tasks. It is an scalable and optimized implementation of the gradient boosting technique proposed by Friedman et al. [25] that was further refined by Chen et al. [26] to enhance the accuracy and generalizability of the resulting models. Unlike the traditional gradient boosting techniques, which generally use only the first-order derivative of the loss function to steer optimization, XGB utilizes both first- and second-order derivatives, which provide more accurate estimation of the direction and global magnitude of the gradient. This leads to more accurate model updates during training. XGB also implements both L1 (lasso) and L2 (ridge) regularization methods to minimize overfitting and foster the development of robust and generalizable predictive models.

The effectiveness of the XGB algorithm was enhanced by its unique ability to process missing values, which streamlined the preprocessing phase. Additionally, its architecture enabled parallel and distributed computing, greatly expediting computations compared to traditional gradient-based methods. These benefits resulted in XGB's superior predictive performance as reported in several studies [27, 28, 29].

A-6. K-Means Clustering

In the 1950s and 1960s, different researchers independently proposed the K-means clustering algorithm which is an unsupervised ML algorithm. This algorithm groups inputs into a predefined plurality of clusters [30, 31, 32]. Generally, the number of clusters is assumed as a hyperparameter and is used to initialize the centroids of randomly chosen clusters in the data set [33].

The K-means method starts with adding predefined centroids to the dataset. Each data point is then associated with the closest centroid, usually using the distance method of Euclidean measure. After association, the centroids are updated by calculating the mean of each cluster. This whole procedure continues to repeat until the association reaches stability and there are no further changes in the ensuing steps. Due to its straightforward operation and efficient procedure, K-means is widely and frequently used in many fields [33]. The main purpose of the algorithm is to reduce the within-cluster sum of square error

so that the clusters can be grouped closer to one another, hence creating homogeneity in all clusters [33, 34]. It should also be noticed that increasing the number of clusters yields to reduce the sum of square error since more clusters fit the data distribution closely [35].

Appendix B: Graphical representation of TUV Datasets of cores-B and C used for ML models

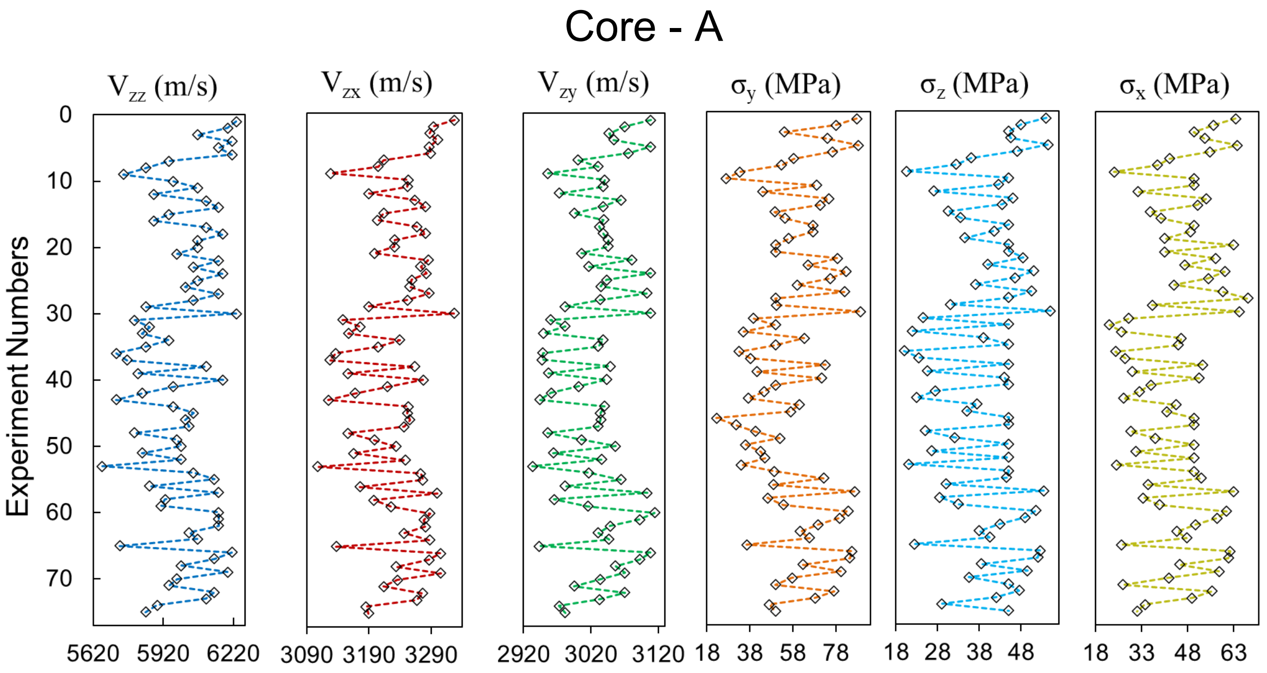

Figure S1: A suite of TUV dataset for core-A.

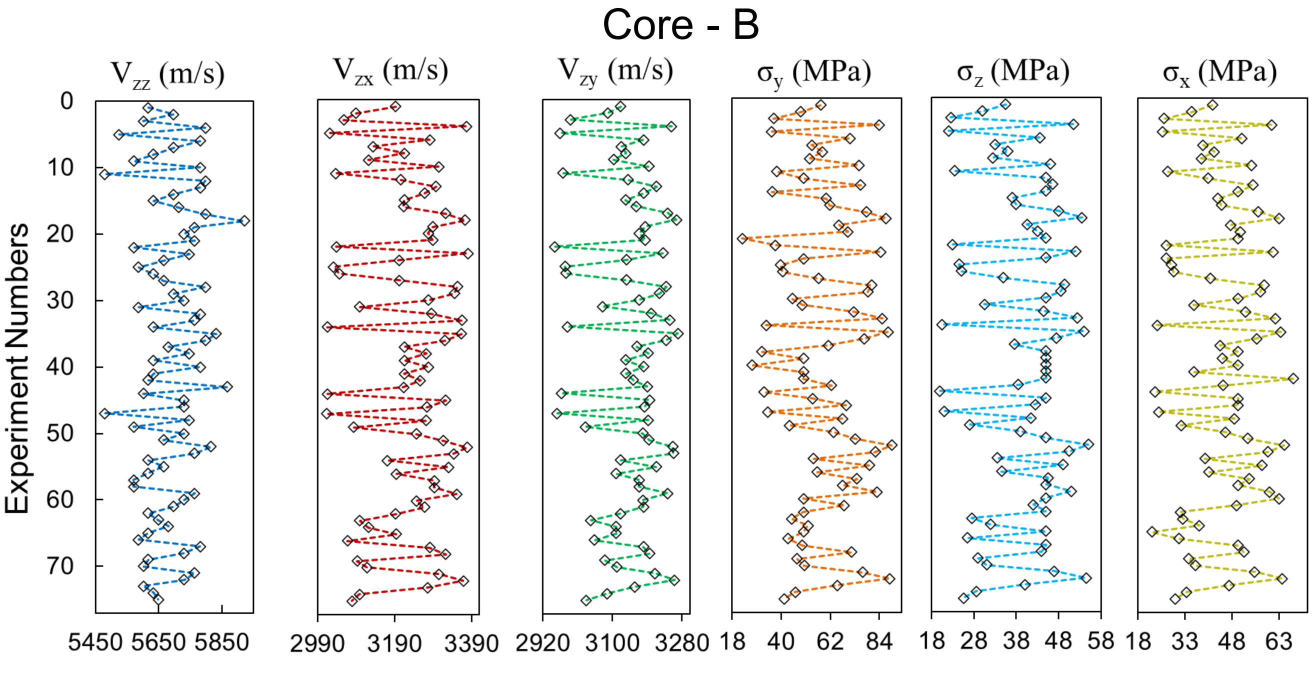

Figure S2: The suite of TUV dataset for core-B

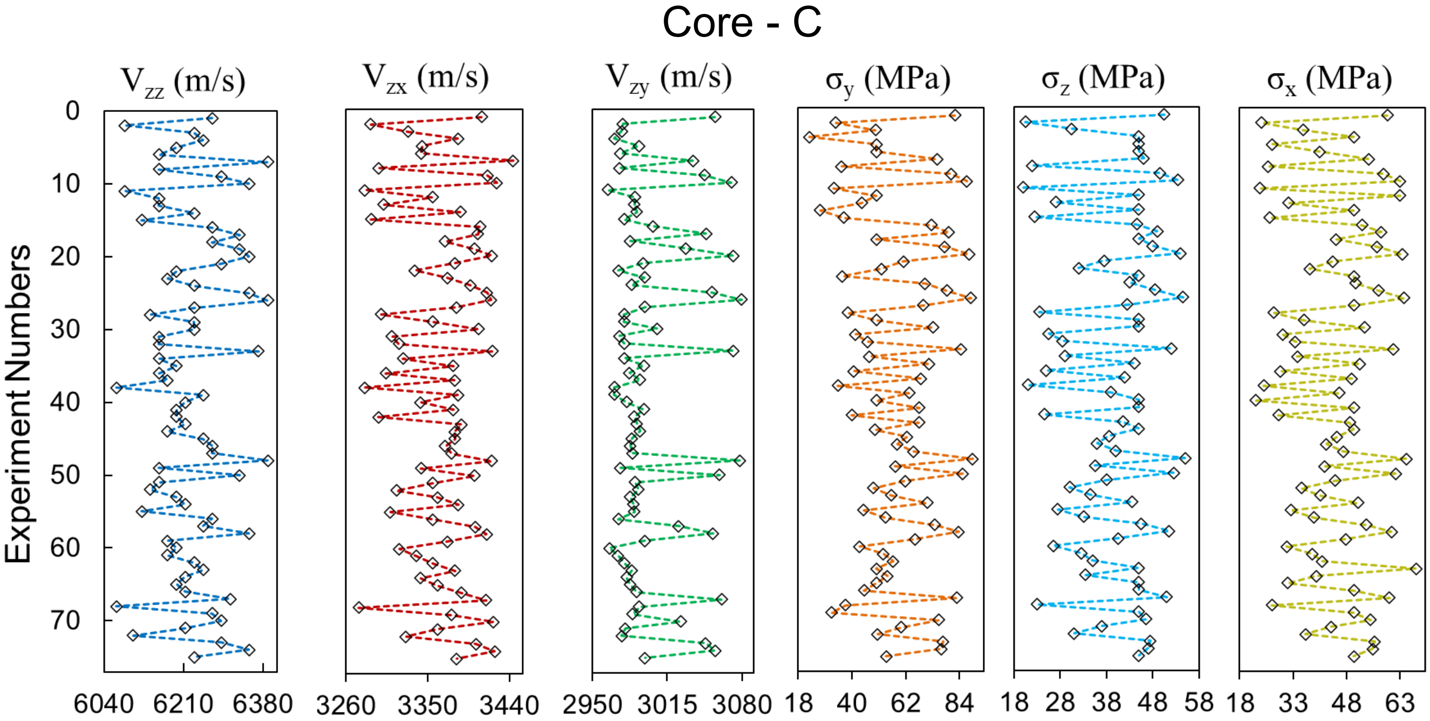

Figure S3: The suite of TUV dataset for core-C

Appendix C: Histograms of datasets for cores-B, and C.

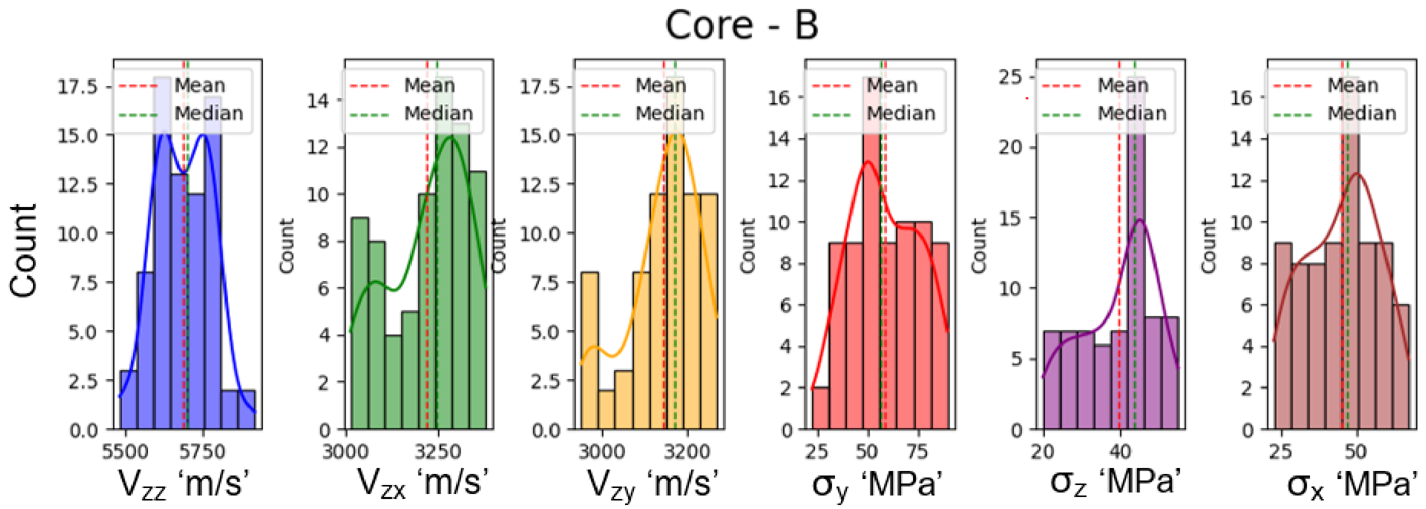

Figure S4: Histograms showing the data distribution of TUV data used to develop models for core-B

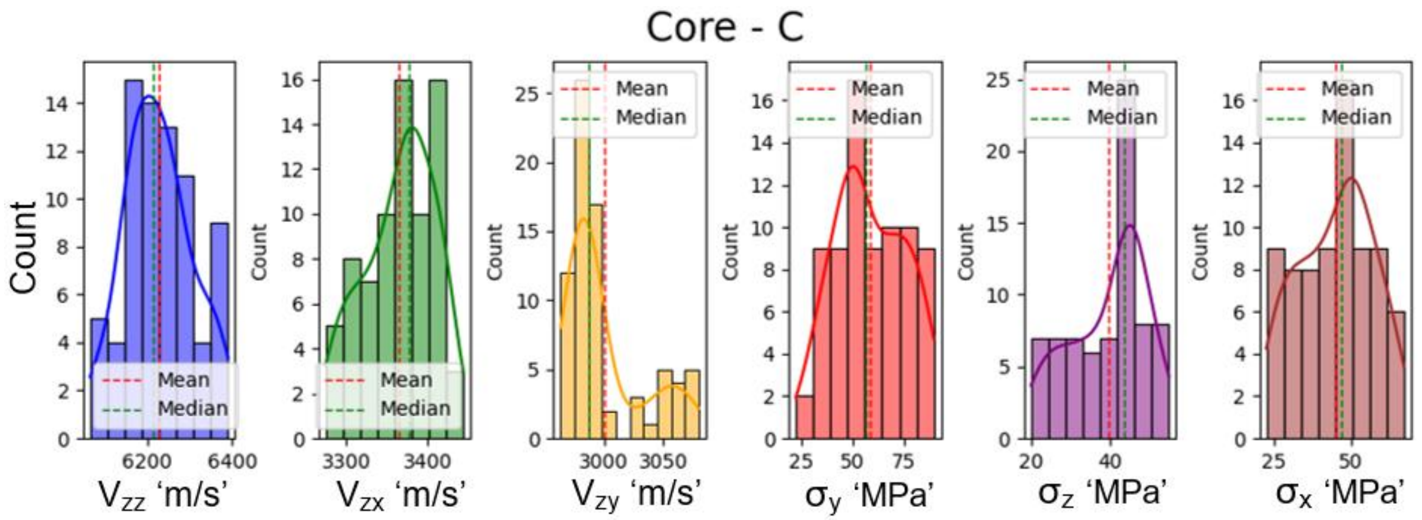

Figure S5: Histograms showing the data distribution of TUV data used to develop models for core-C

Appendix D: Violin Plots showing distribution of features for cores-A, B, and C.

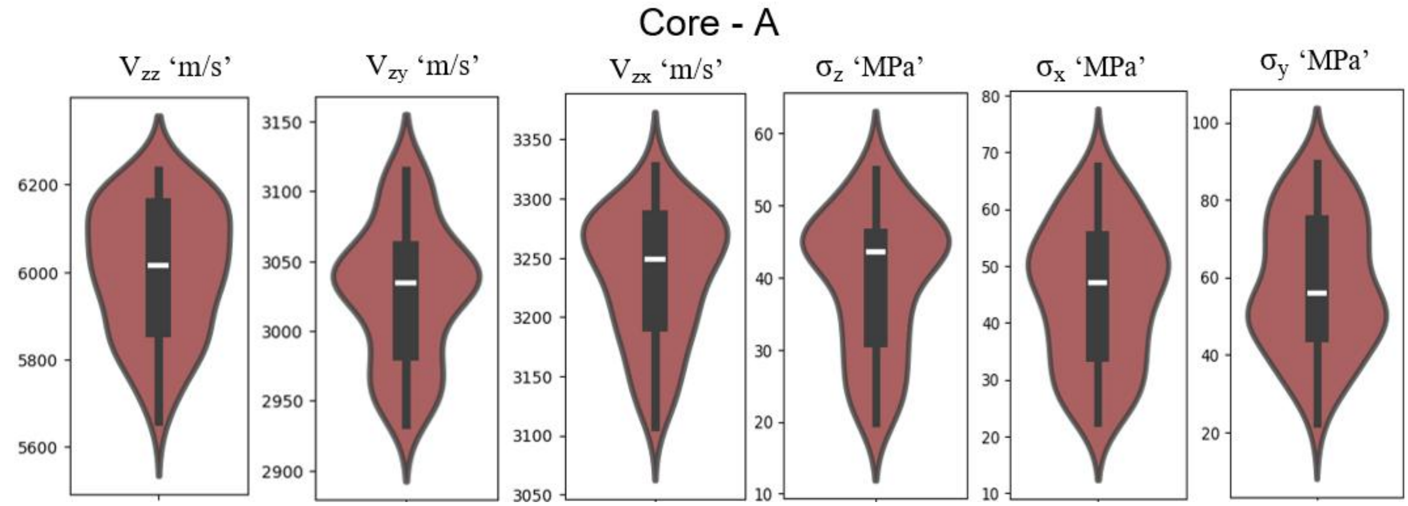

Figure S6: Violin plots showing distribution of features for core-A

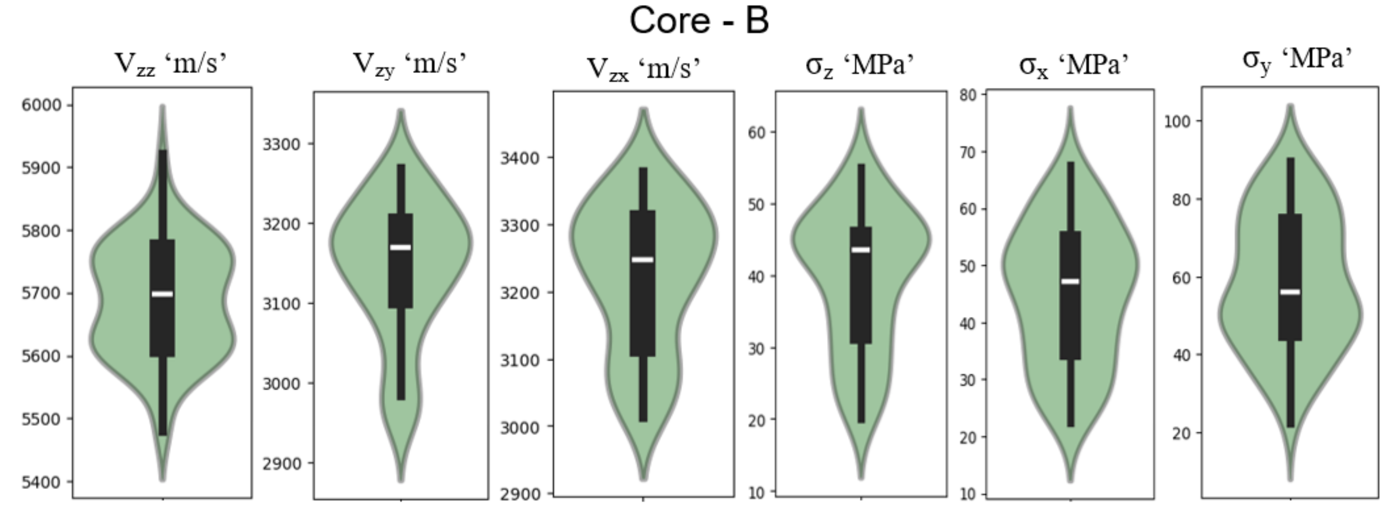

Figure S7: Violin plots showing distribution of features for core-B

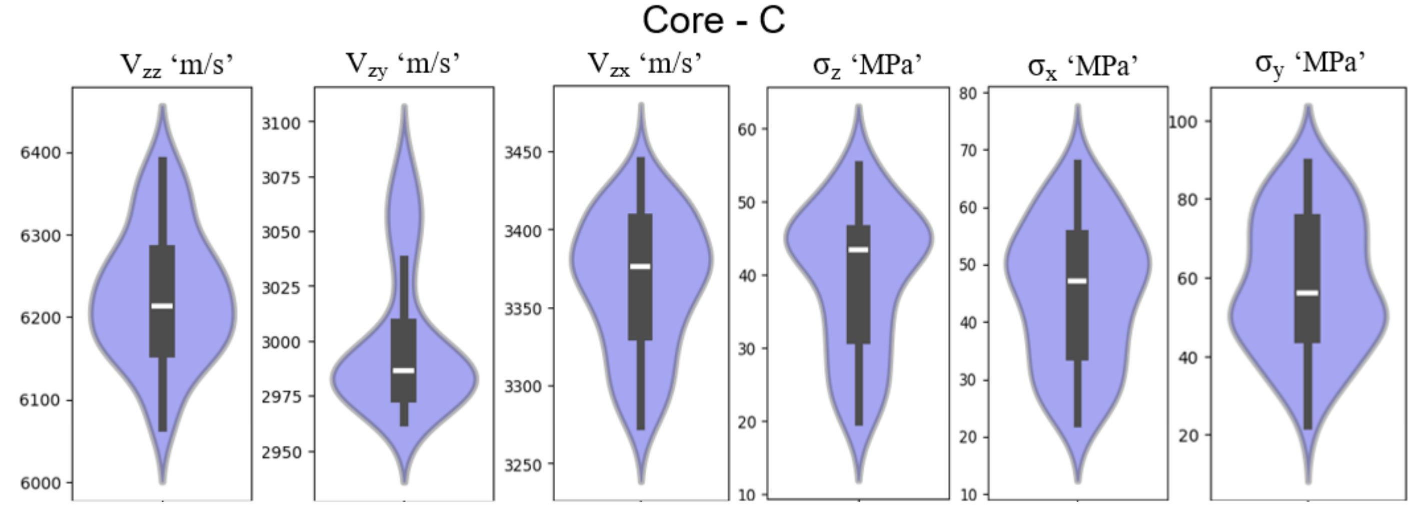

Figure S8: Violin plots showing distribution of features for core-C

Appendix E: Heatmaps shows collinearity between all pairs of features.

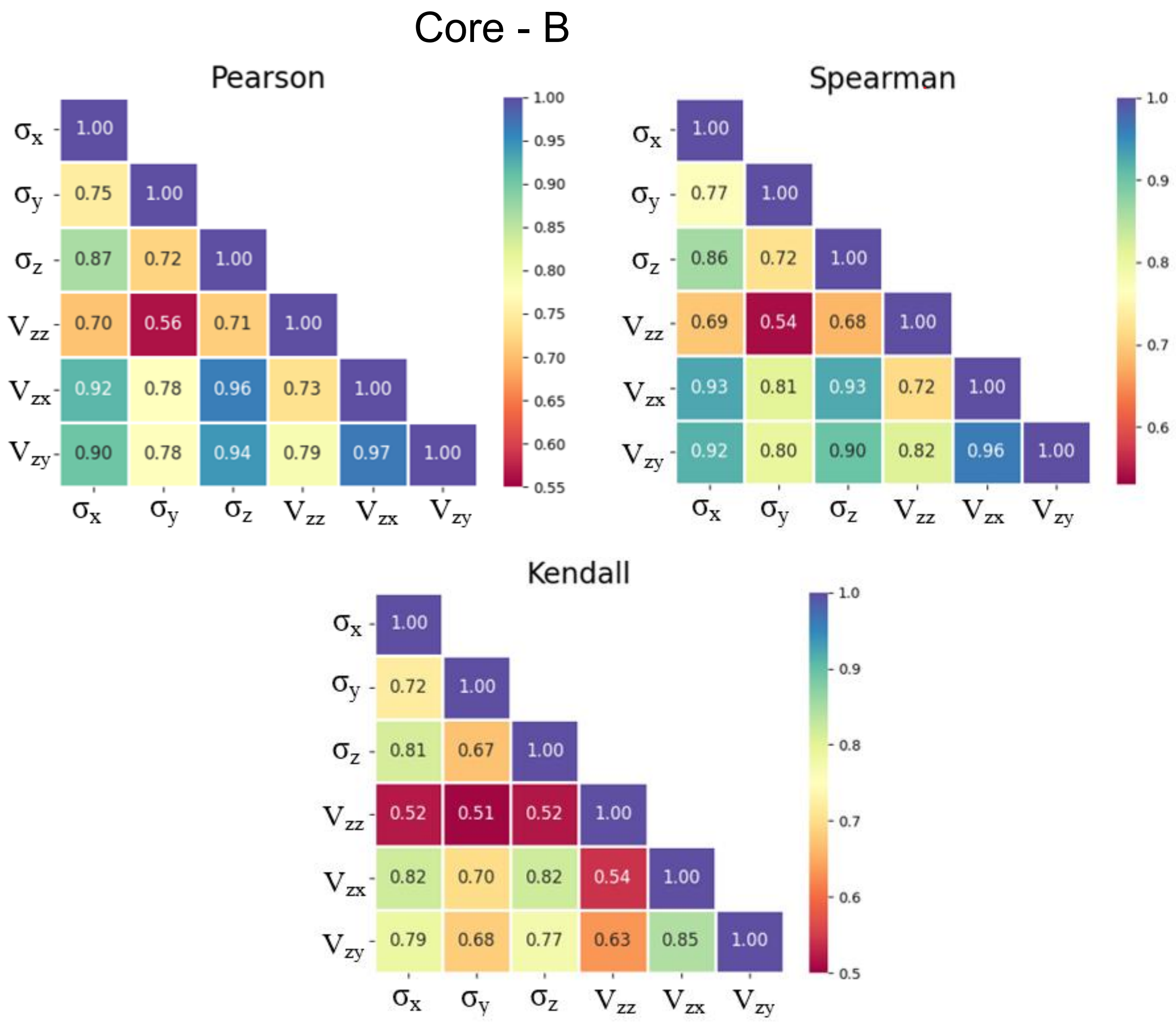

Figure S9: Heatmaps showing collinearity between all pairs of features for core-B.

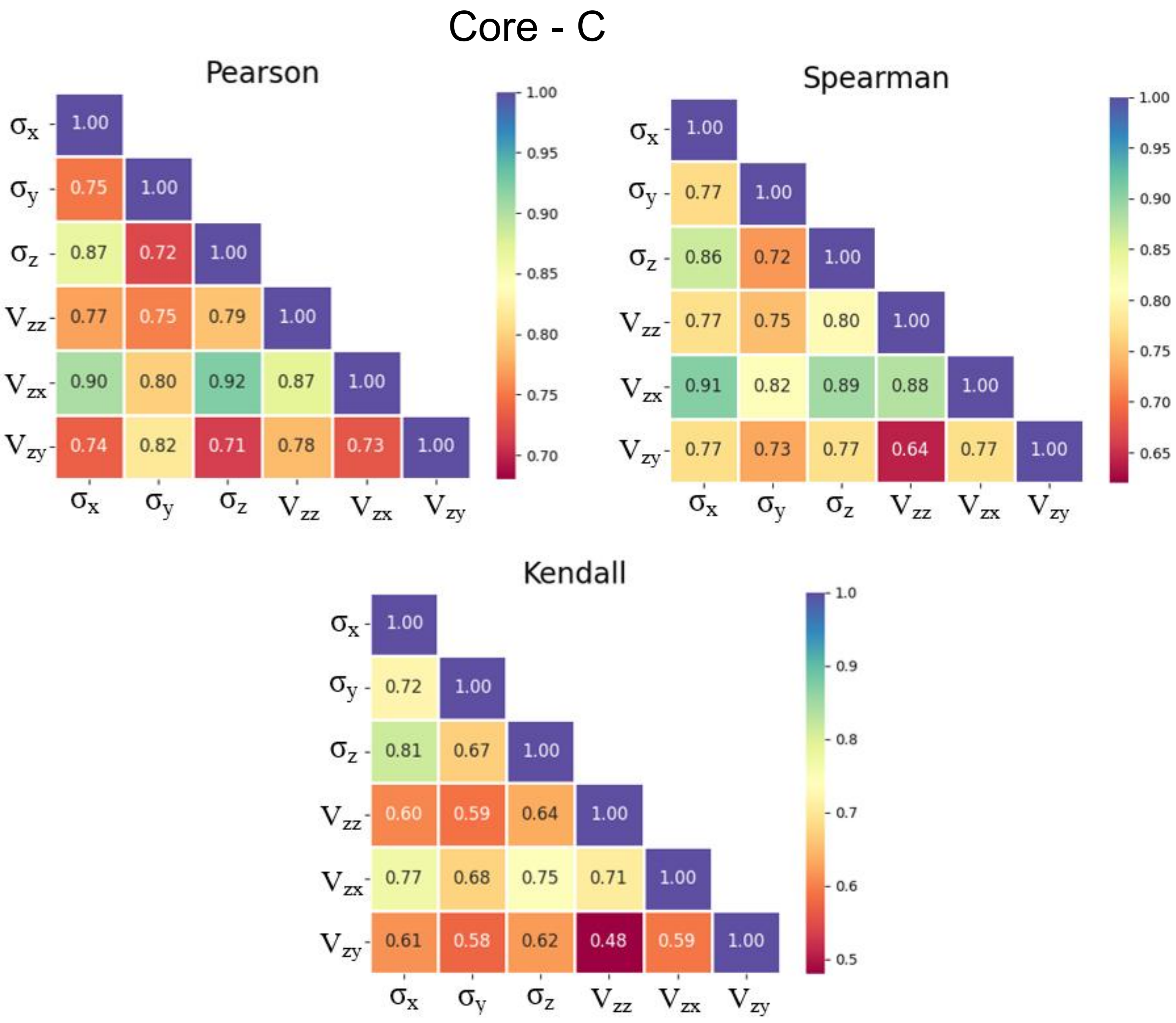

Figure S10: Heatmaps showing collinearity between all pairs of features for core-C.

Appendix F: Relative importance of input features for σ_y, σ_z, and σ_x stress models. Last figure shows the pairplot of each pair of input and features.

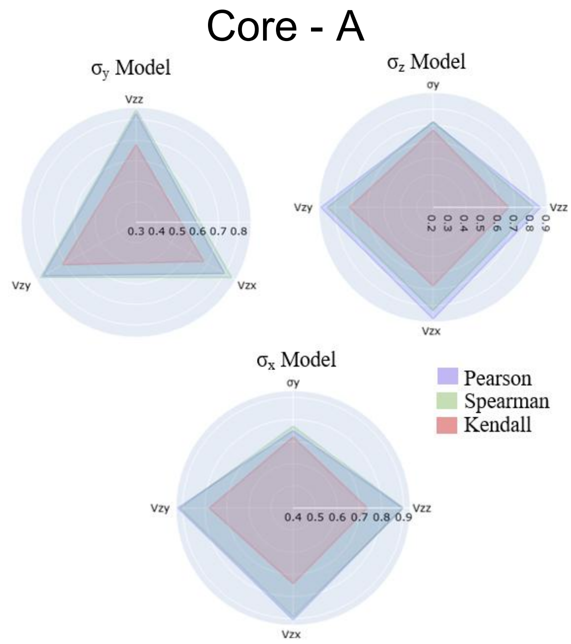

Figure S11: Radar chart showing relative importance of input features for σ_x, σ_y, and σ_z stress models of core-A.

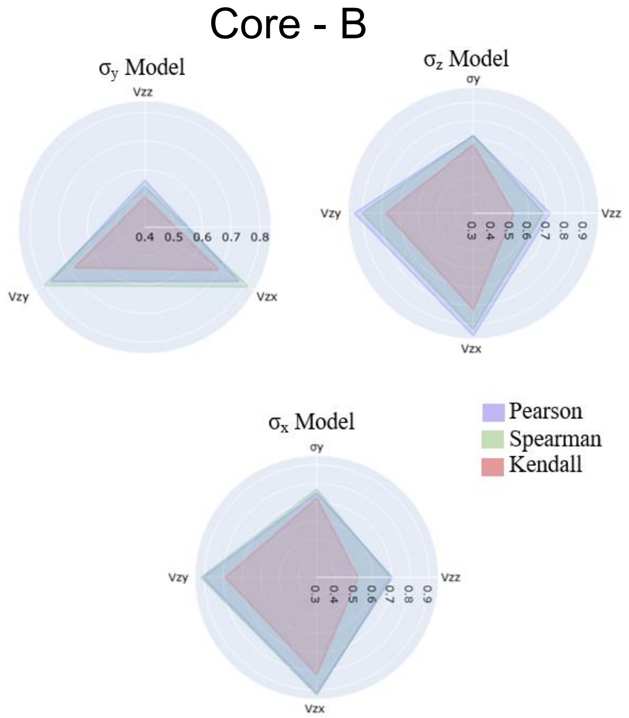

Figure S12: Radar chart illustrates relative importance of input features for σ_x, σ_y, and σ_z stress models of core-B.

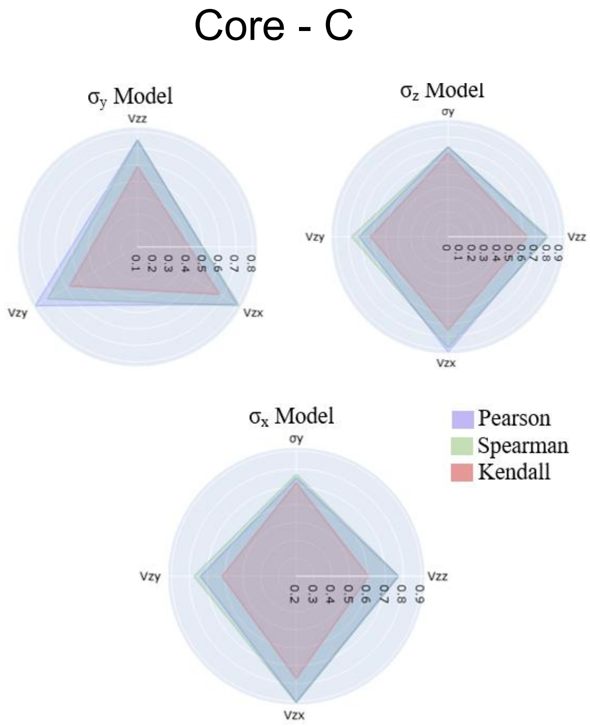

Figure S13: Radar chart illustrates relative importance of input features for σ_x, σ_y, and σ_z stress models of core-C.

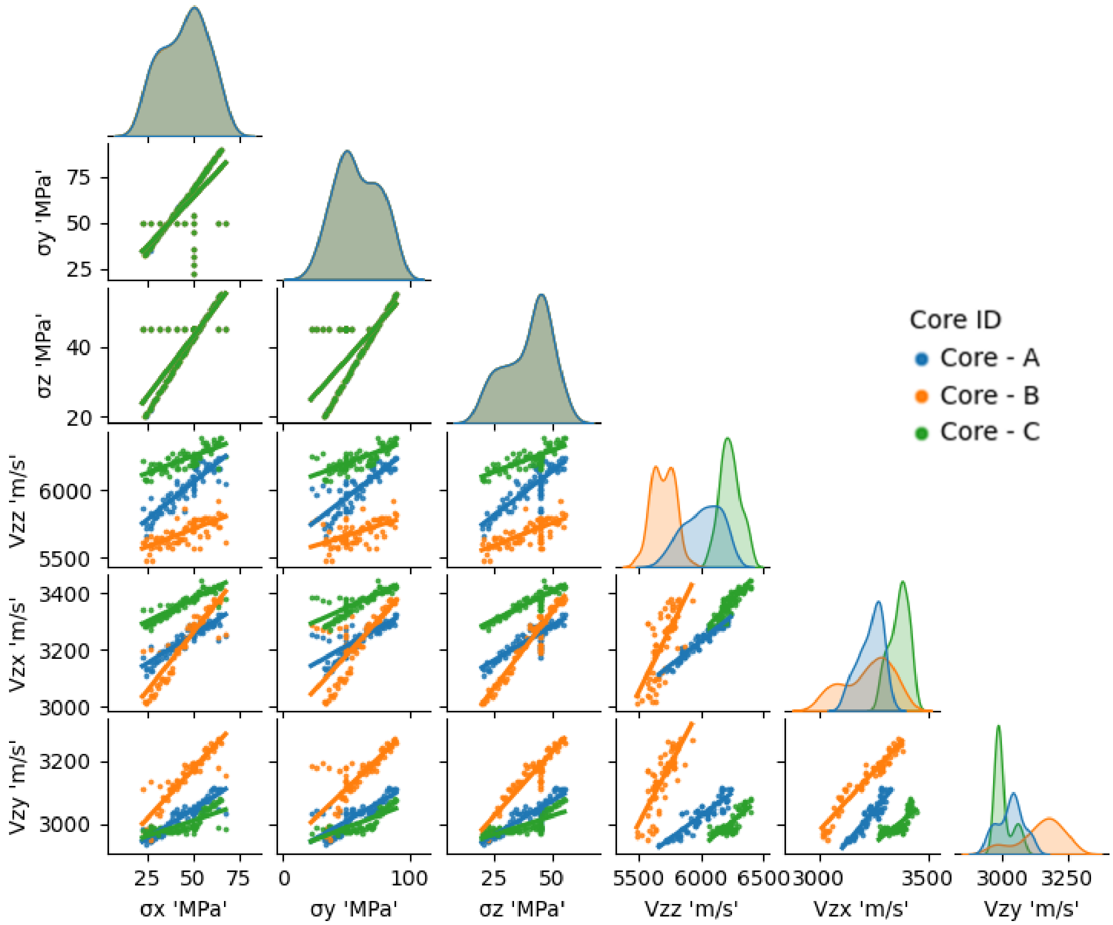

Figure S14: Pair plot illustrates cross plots between the features' pairs for all cores.

Appendix G: Details about stepwise process of construction and optimization of ML/DL models.

In Deep Neural Network (DNN) models, the architecture is fundamentally governed by two primary components: the hidden layers count and number of neurons count in each layer. A representative neural architecture adopted in this study as illustrated in Figure S15. Another essential element influencing model performance is the activation function. To systematically determine the optimal set of hyperparameters, a sensitivity analysis was conducted. This analysis followed a staged approach to ensure a coherent and structured process in developing DNN architecture. Specifically, the sensitivity assessment of neuron count per hidden layer was performed across a range of 5 to 40 neurons to identify the most effective configuration. The corresponding coefficient of determination (R^2) and Root Mean Squared Error (RMSE) values for both testing/validation and training phases, using different neuron counts, were evaluated for the DNN models

predicting stresses σ_x, σ_y, and σ_z for core-A, as shown in Figure 5 of the main manuscript. The optimal number of neurons, based on the lowest validation RMSE and highest R^2, was found to be 11 for σ_y, 15 for σ_z, and 21 for σ_x. At these optimal configurations, the validation RMSE and R^2 were recorded as 2.59 and 0.986 for σ_y, 1.92 and 0.971 for σ_z, and 1.80 and 0.975 for σ_x, respectively. The detailed neuron sensitivity analyses for DNN models applied to cores B and C are provided in Appendix-F.

The optimization of the hidden layers count, the evaluation of neuron counts, and the selection of appropriate activation functions were carried out during the model construction phase, wherein the input, hidden, and output layers were defined. A dropout layer was additionally incorporated between the hidden and input layers to enhance generalization. Following this, the DNN models were compiled by selecting suitable optimizers, learning rates, loss functions, and accuracy metrics to facilitate effective model training and performance evaluation. The training process involved simulations using various combinations of hyperparameters across multiple epochs and batch sizes. Ultimately, the most effective set of hyperparameters was identified and adopted for final model training, as detailed in Table 4 in the main manuscript. Moreover, to prevent overfitting and mitigate performance degradation on the validation set, the early stopping technique was implemented during training.

An essential step in the development of optimized models involved executing multiple realizations to identify the most effective random seed functions, which established a unique computational identity for the DNN implementation in this study. The integration of the optimal hyperparameter values with the best-performing realizations resulted in the development of fully optimized DNN models for these stress components. The discrepancy between the experimentally measured and DNN-predicted stress values was quantified using the selected loss function, namely the mean squared error.

Alongside the DNN model, four additional predictive models, comprising two deep learning (DL) models, namely GRU and CNN, and two ML models including RF and XGB, were trained and tested/validated using the same modified TUV dataset. During the training process, the optimal values of

the respective hyperparameters for each model were determined. The tuning of hyperparameters of the DL and ML models was done using the Grid Search Cross-Validation (CV) approach.

The GRU model was developed following a structured workflow. Once the data was preprocessed and partitioned, the network architecture was defined with an initial GRU layer (serving as the input layer) followed by a second GRU layer, then a dropout layer to mitigate overfitting, and concluding with an output layer to produce the predictions. The steps for model compilation, training, and validation is almost similar to DNN. Hyperparameter tuning was performed with grid search cross-validation on specified ranges of GRU unit counts (10–200), dropout rates (0.1–0.5), batch sizes (32–64), and learning rates (0.001–0.1). The combination that resulted in the lowest loss was chosen to finalize the architecture, which was subsequently applied to the validation/test dataset. The optimized hyperparameter values are summarized in Table 4 in the main manuscript.

The CNN architecture was engineered to assess its capability in addressing regression tasks on a tabular dataset. The network began with an input layer, succeeded by two one-dimensional convolutional layers (Conv1D) configured with specific filter counts, kernel sizes, and ReLU activation functions. Outputs from these convolutional layers were then flattened and passed to a fully connected dense layer, also employing ReLU activation. A final layer is an output layer that is comprised of one neuron and uses linear activation function to generate continuous predictions. Hyperparameter optimization was conducted via grid search cross-validation over ranges for kernel size (2–10), filter count (8–64), dense layer units (8–64), learning rate (0.0001–0.1), batch size (8–64), and optimizer choice (Adam, Nadam, SGD). The parameter set yielding the lowest loss was chosen, and the resulting CNN model was applied to the validation dataset to evaluate its generalization performance on unseen data.

Additionally, two machine learning models such as XGB and RF were constructed. Hyperparameters for these models were optimized using a grid search cross-validation. The predictive accuracy of the proposed five DL and ML models was then evaluated and compared to identify the most suitable model for

field deployment. The finalized hyperparameter settings for each model are detailed in Table 4 in the main manuscript.

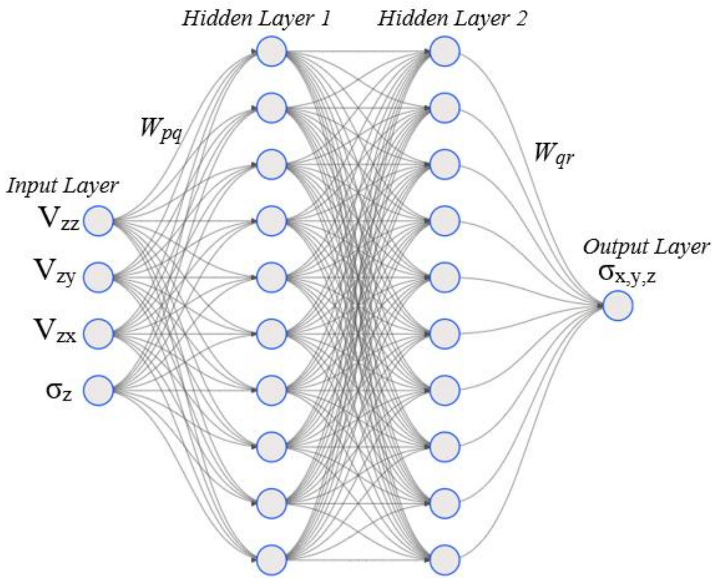

Figure S15: DNN topology showing neurons structure.

Appendix H: Analysis of Neurons sensitivity using accuracy measures of σ_x, σ_y, σ_z models for Core-B and -C.

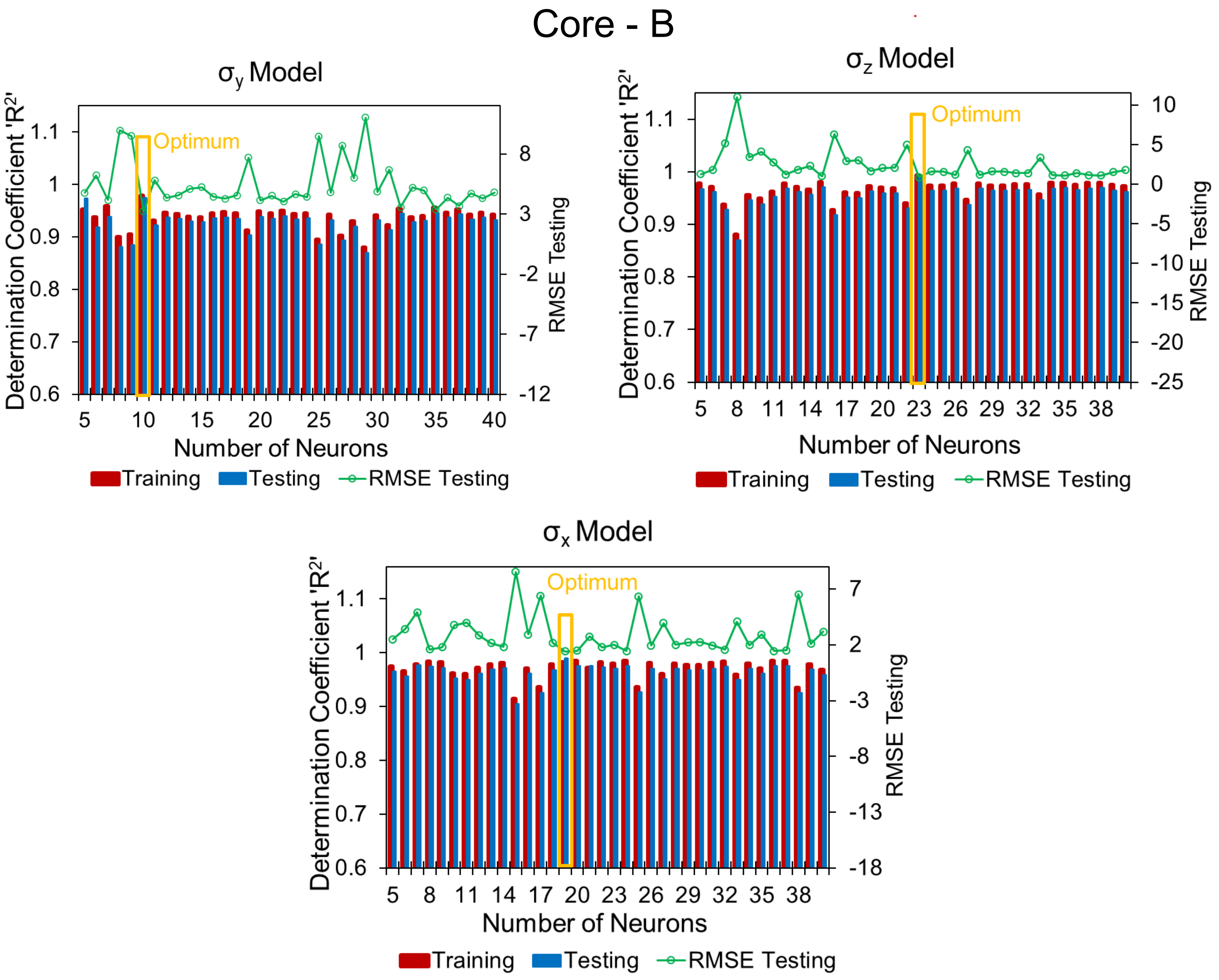

Figure S16: Analysis of Neurons sensitivity using accuracy measures of σx, σy, σz models for core-B.

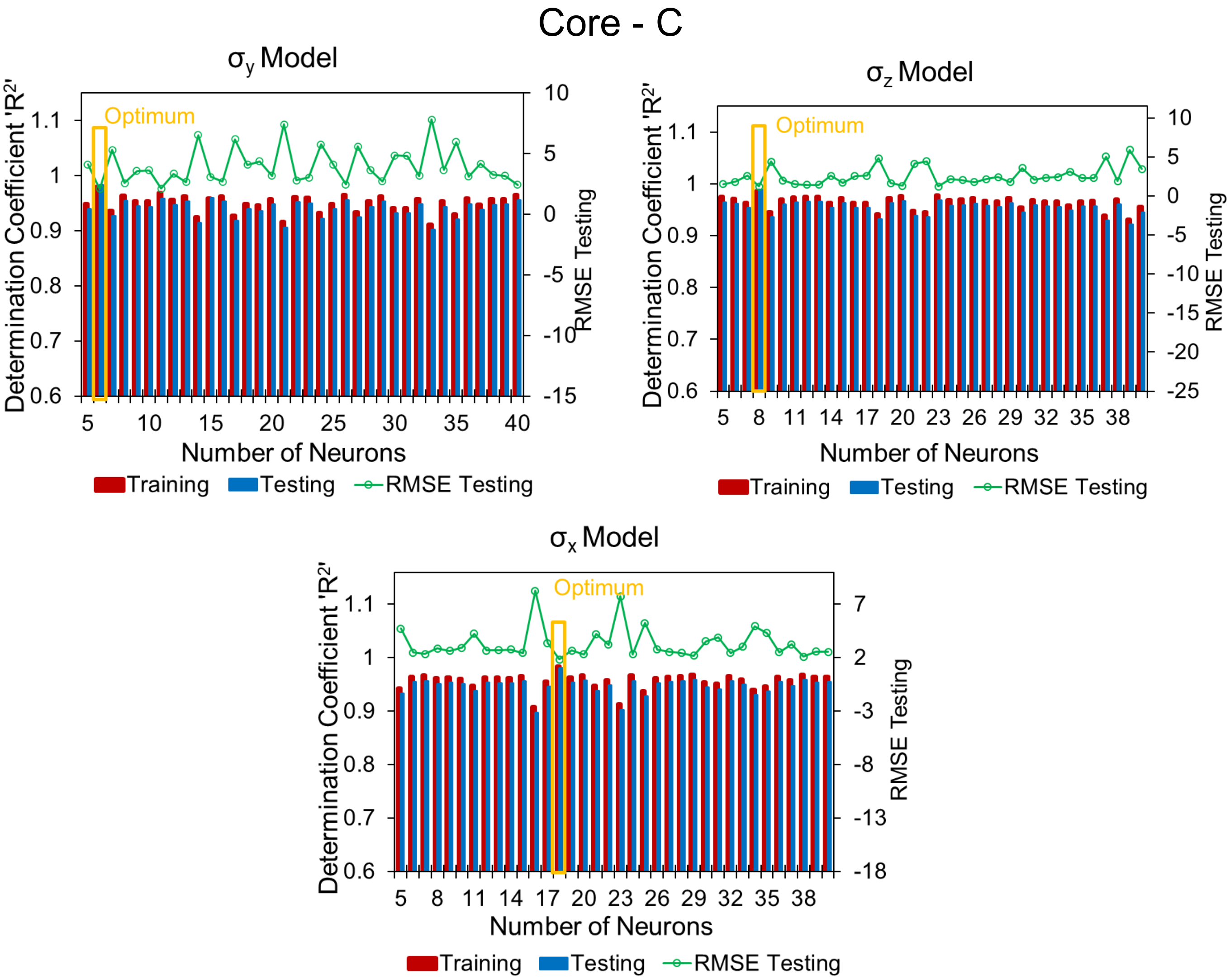

Figure S17: Analysis of Neurons sensitivity using accuracy measures of σx, σy, σz models for core-C.

Appendix I: Optimum values of hyperparameters of the proposed DL/ML models

Table S1: Optimum values of hyperparameters used for σ_Z, σ_X, σ_Y models of Core-B.

Model Type	Hyperparameter	Tested Values	Selected value for 'σ_z'	Selected value for 'σ_y'	Selected value for 'σ_x'
DNN	Hidden layers count	1-5	2	2	2
	Neurons count	5-40	11	21	15
	Activation function	Tanh, LeakyReLU, ReLU	ReLU	LeakyReLU	Tanh
	Dropout Rate	0.1-0.45	0.15	0.15	0.15
	Optimizer	Nadam, Adam, Sgd	Nadam	Nadam	Adam
	Learning rate	0.1 – 0.00001	0.001	0.0001	0.0001
	Loss Function	RMSE, MSE	MSE	MSE	MSE
	Batch size	64, 32, 16, 8	8	16	8
GRU	GRU Units	10-220	40	50	45
	Optimizer	Nadam, sgd, Adam	Nadam	Nadam	Nadam
	Learning rate	0.1 – 0.00001	0.005	0.005	0.005
	Activation Function	Tanh, LeakyReLU, ReLU	LeakyReLU	ReLU	ReLU
	Batch Size	64, 32, 16, 8	16	16	16
	Dropout rate	0.1-0.45	0.20	0.20	0.20
CNN	Number of Filters	8-32	10	8	8
	Kernel Size	2-12	4	4	4
	Batch Size	64, 32, 16, 8	16	8	8
	Dense Units	8-64	32	16	16
	Optimizer	Nadam, sgd, Adam	Adam	Adam	Adam
	Activation Function for Convolution layer	Tanh, LeakyReLU, ReLU	LeakyReLU	Tanh	ReLU
RF	Sample count necessary to split the internal node	2-15	10	10	8
	Sample count at the leaf node	1-6	2	2	2
	Total count of trees in the forest	50-2000	900	800	1000
	Maximum depth of the trees	3-12	6	5	6
XGB	Criterion	-	friedman mse	friedman mse	friedman mse
	Count of boosting stages to be performed	50-1200	220	150	250
	Minimum sample split	1-6	3	3	2
	Minimum sample leaf	1-6	2	3	2
	Alpha	-	0.02	0.02	0.03
	Learning rate	0.1-0.0001	0.001	0.001	0.001
	Max. depth	3-12	5	6	5

Table S2: Optimum values of hyperparameters used for σ_Z, σ_X, σ_Y models of Core-C.

Model Type	Hyperparameter	Tested Values	Selected value for 'σ_z'	Selected value for 'σ_y'	Selected value for 'σ_x'
**DNN**	Hidden layers count	1-5	2	2	2
	Neurons count	5-40	11	21	15
	Activation function	Tanh, LeakyReLU, ReLU	ReLU	LeakyReLU	Tanh
	Dropout Rate	0.1-0.45	0.15	0.15	0.15
	Optimizer	Nadam, Adam, Sgd	Nadam	Nadam	Adam
	Learning rate	0.1 – 0.00001	0.001	0.0001	0.0001
	Loss Function	RMSE, MSE	MSE	MSE	MSE
	Batch size	64, 32, 16, 8	8	16	8
**GRU**	GRU Units	10-220	40	50	45
	Optimizer	Nadam, sgd, Adam	Nadam	Nadam	Nadam
	Learning rate	0.1 – 0.00001	0.005	0.005	0.005
	Activation Function	Tanh, LeakyReLU, ReLU	LeakyReLU	ReLU	ReLU
	Batch Size	64, 32, 16, 8	16	16	16
	Dropout rate	0.1-0.45	0.15	0.20	0.15
	Loss Function	MSE, RMSE	MSE	MSE	MSE
**CNN**	Number of Filters	8-32	10	8	8
	Kernel Size	2-12	4	4	4
	Batch Size	64, 32, 16, 8	16	8	8
	Dense Units	8-64	32	16	16
	Optimizer	Nadam, sgd, Adam	Adam	Adam	Adam
	Loss Function	MSE, RMSE	MSE	MSE	MSE
	Activation Function for Convolution layer	Tanh, LeakyReLU, ReLU	LeakyReLU	Tanh	ReLU
**RF**	Sample count necessary to split the internal node	2-15	10	10	8
	Sample count at the leaf node	1-6	2	2	2
	Total count of trees in the forest	50-2000	900	800	1000
	Maximum depth of the trees	3-12	6	5	6
**XGB**	Criterion	-	friedman mse	friedman mse	friedman mse
	Count of boosting stages to be performed	50-1200	220	150	250
	Minimum sample split	1-6	3	3	2
	Minimum sample leaf	1-6	2	3	2
	Alpha	-	0.02	0.02	0.03
	Learning rate	0.1-0.0001	0.001	0.001	0.001
	Max. depth	3-12	5	6	5
	Loss	MSE, RMSE	RMSE	RMSE	RMSE

Appendix J: Cross plots between experimental and predicted stresses σ_x, σ_y, σ_z for the CNN, XGB, DNN, RF, GRU models.

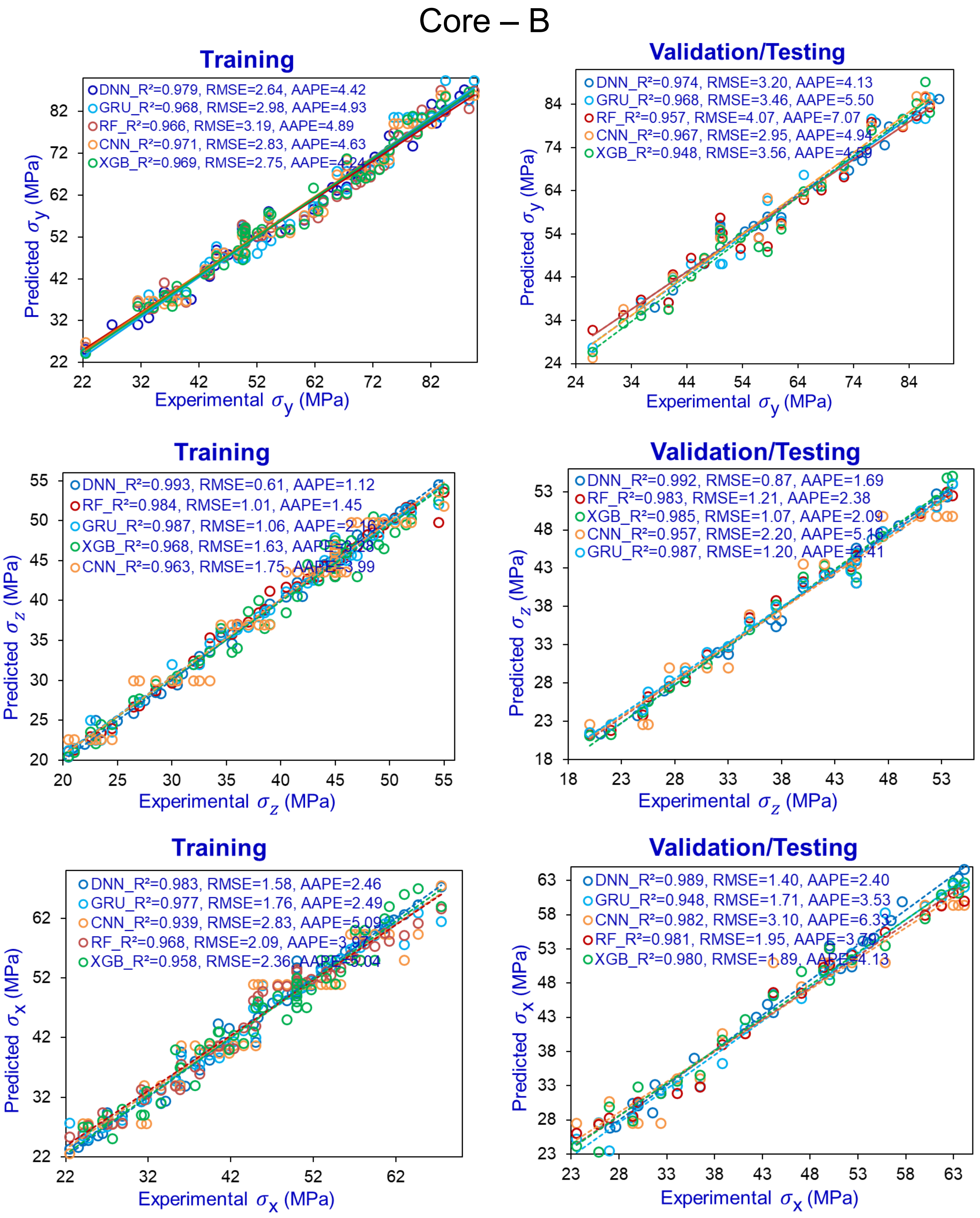

Figure S18: Cross plots between experimental and predicted stresses σ_x, σ_y, σ_z for the CNN, XGB, DNN, RF, GRU models for Core-B.

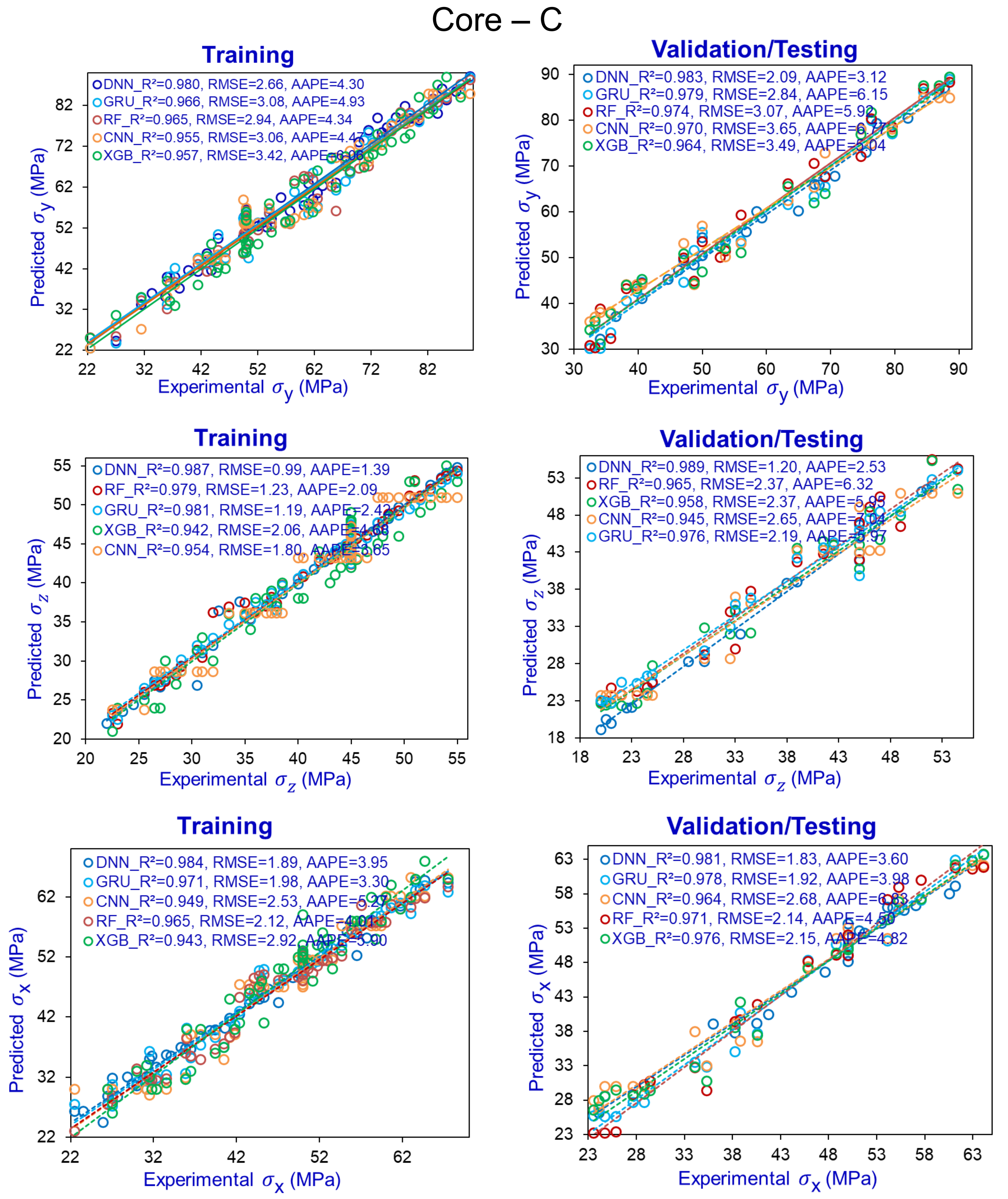

Figure S19: Cross plots between experimental and predicted stresses σ_x, σ_y, σ_z for the CNN, XGB, DNN, RF, GRU models for Core-C.

Appendix K: Comparison between experimental and predicted stresses σ_x, σ_y, σ_z for CNN, XGB, DNN, RF, GRU models for Core-A, -B and -C.

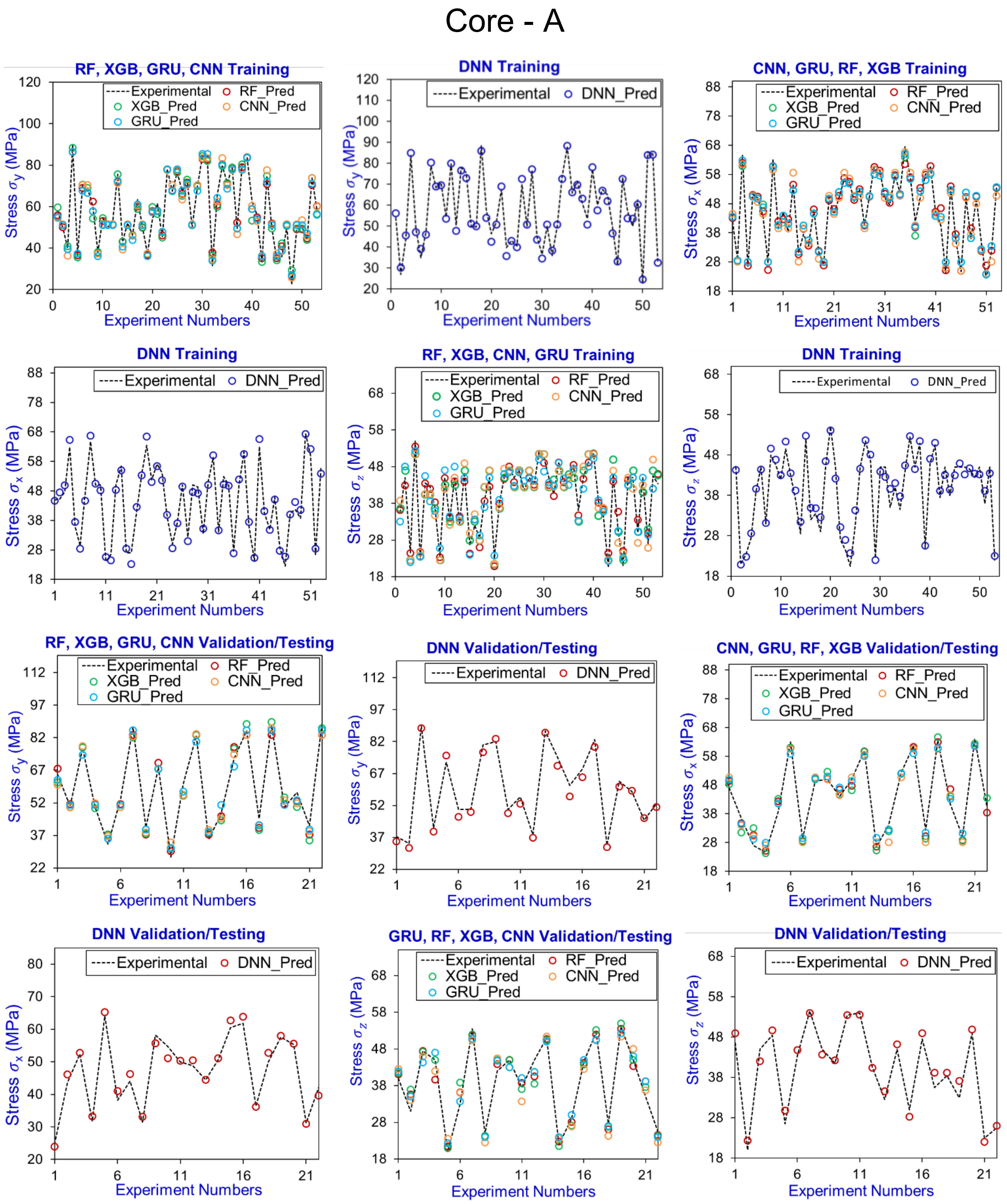

Figure S20: Comparison between experimental and predicted stresses σ_x, σ_y, σ_z for CNN, XGB, DNN, RF, GRU models for Core-A.

Core - B

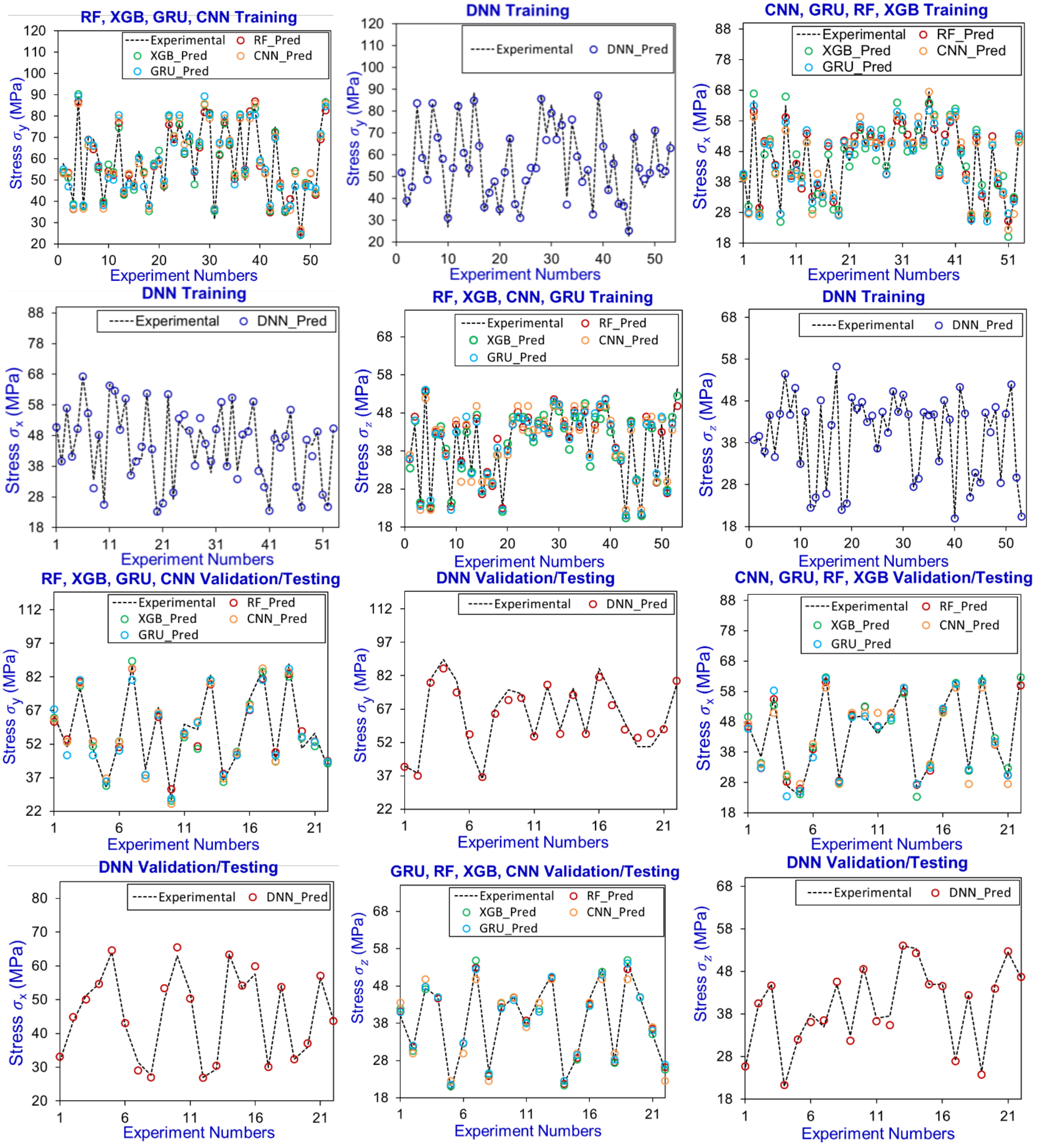

Figure S21: Comparison between experimental and predicted stresses σ_x, σ_y, σ_z for CNN, XGB, DNN, RF, GRU models for Core-B.

Core - C

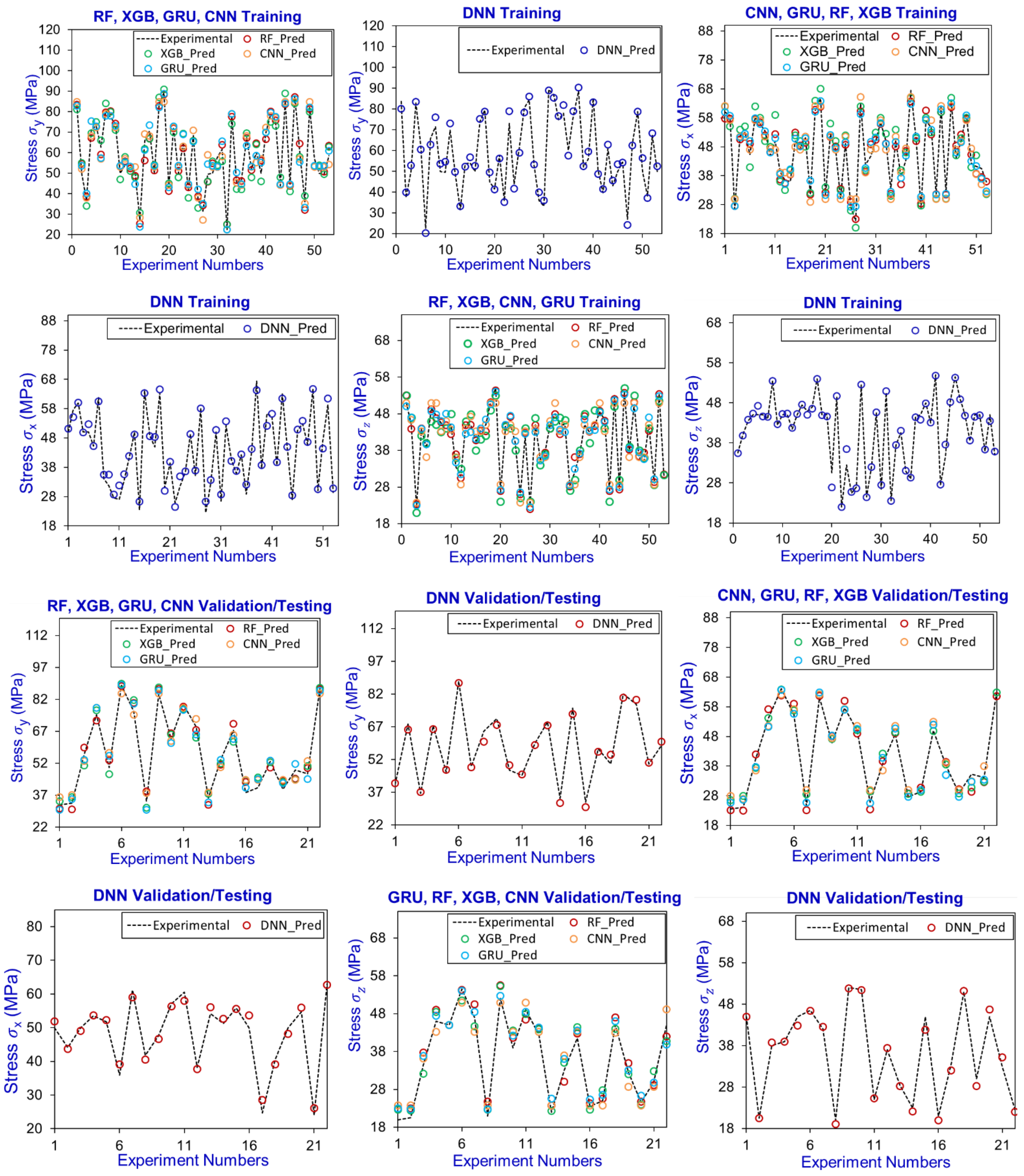

Figure S22: Comparison between experimental and predicted stresses σ_x, σ_y, σ_z for CNN, XGB, DNN, RF, GRU models for Core-C.

Appendix L: Residual errors of testing/validation and training stages of CNN, XGB, DNN, RF, GRU models of σ_x, σ_y, σ_z stresses for core-B and -C.

Core-B

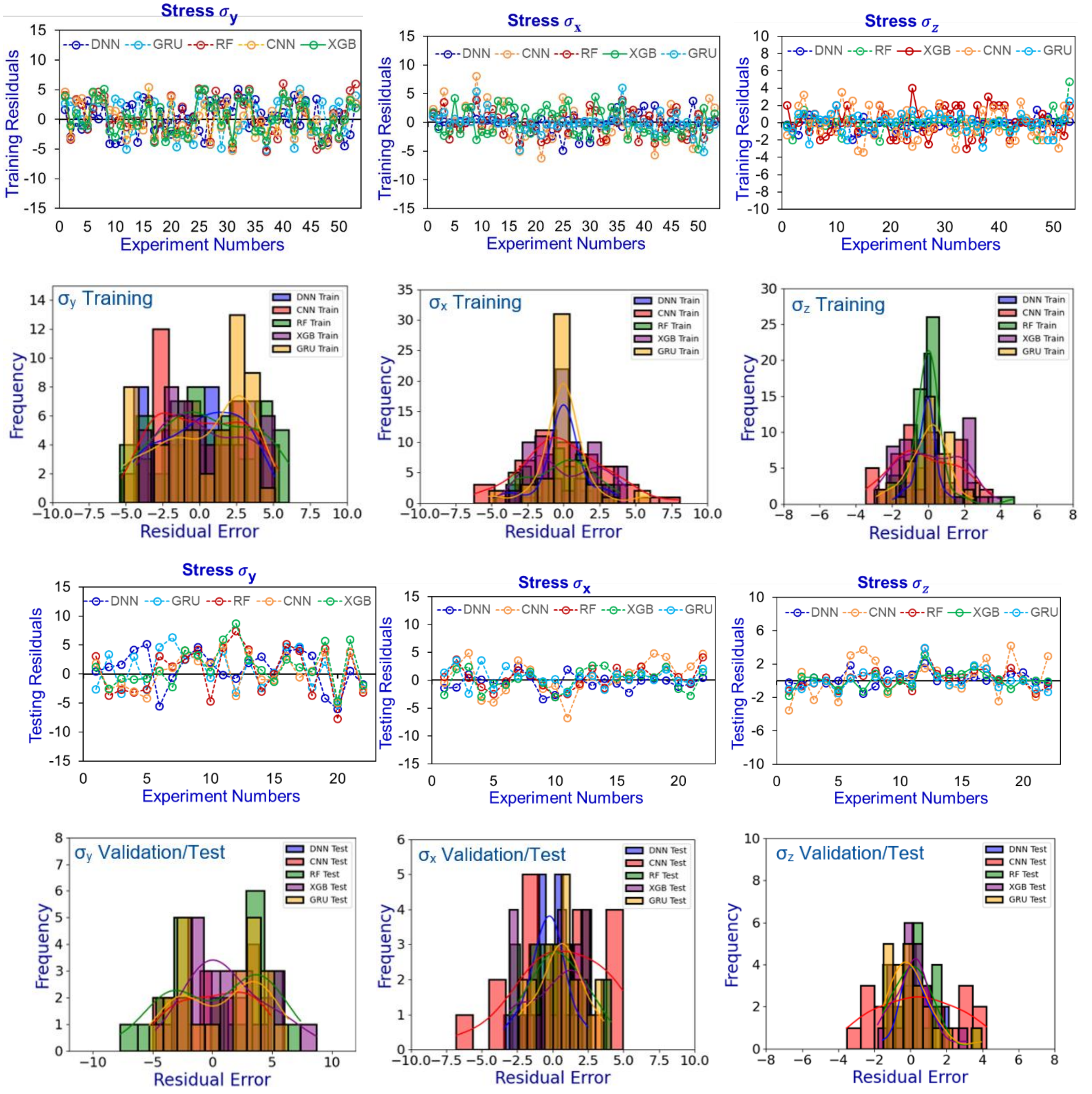

Figure S23: Residual errors of testing/validation and training stages of CNN, XGB, DNN, RF, GRU models of σ_x, σ_y, σ_z stresses for Core-B.

Core-C

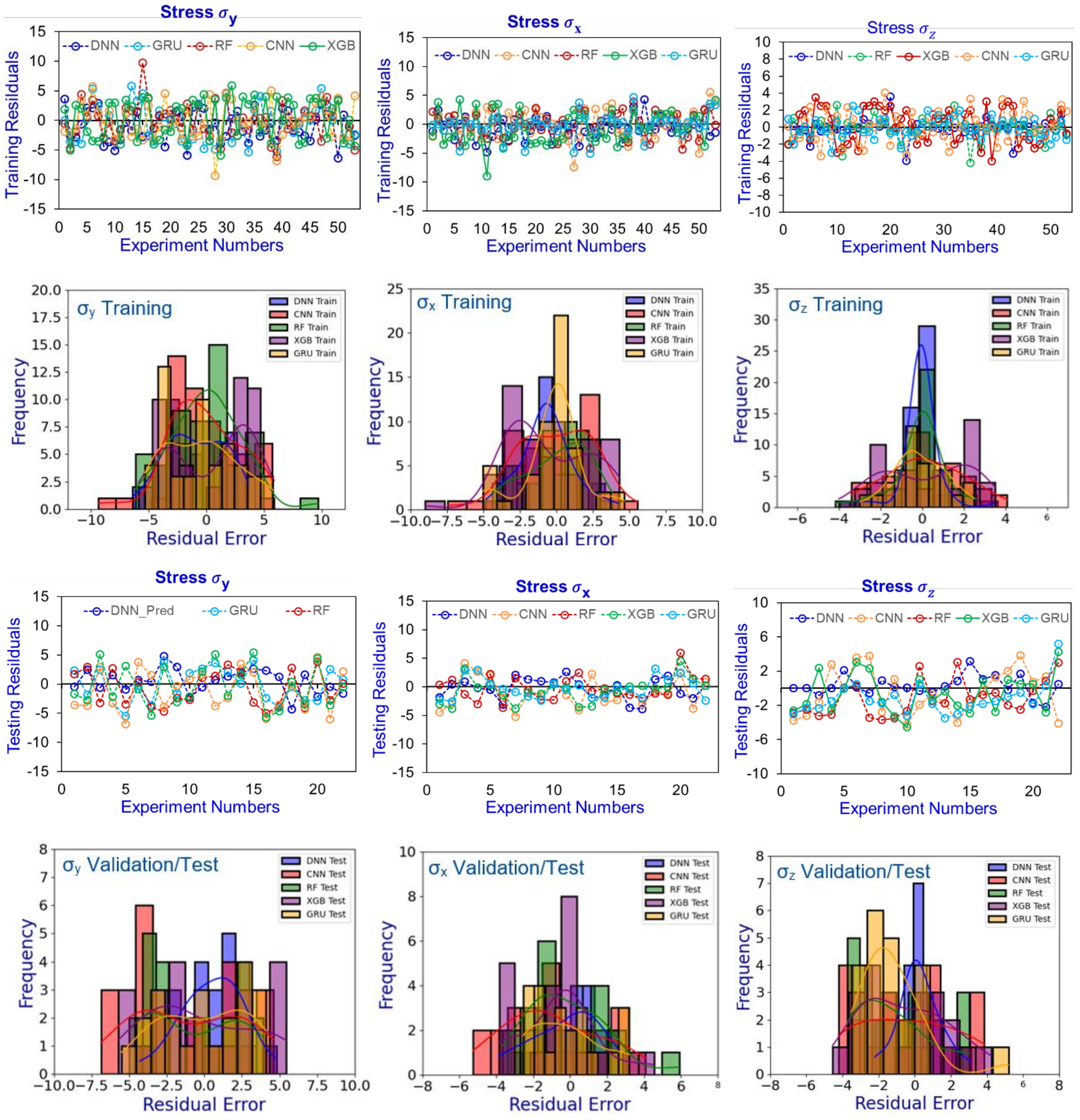

Figure S24: Residual errors of testing/validation and training stages of CNN, XGB, DNN, RF, GRU models of σ_x, σ_y, σ_z stresses for Core-C.

Appendix N: Results of parametric/sensitivity study of DNN models.

Parametric analysis was conducted on the three stress models (σ_x, σ_y, and σ_z) to assess the effects of the input features V_{zx}, V_{zy}, and V_{zz} on the target output σ_y. For V_{zz}, its influence was isolated by varying V_{zz} while holding V_{zy} and V_{zx} constant. The same approach was applied to evaluate the effects of V_{zx} and V_{zy} on σ_y. Equivalent schemes were used to perform parametric analysis for the σ_x and σ_z models. Each analysis revealed distinct response curves that illustrated the physical relationships between individual input features and the predicted stresses. The emergence of characteristic patterns for each feature and output pairing demonstrated the strong generalizability of the predictive models of σ_y, σ_z, and σ_x stresses. The detailed findings from these analyses are shown in Figures S25 through S27.

Distinct curve patterns emerged, characterizing the constitutive relationships between each ultrasonic velocity input (V_{zz}, V_{zx}, and V_{zy}) and the predicted stresses (σ_x, σ_y, and σ_z). The parametric analysis demonstrated that these velocity-stress relationships were inherently non-monotonic, deviating from intuitive linear trends. Similar non-monotonic behaviors between compressional/shear wave velocities and principal stresses were reported by Bunger et al. [36] and Mustafa et al. [37]. These findings suggested that, beyond certain stress thresholds, increases in vertical and horizontal stresses produced rollovers in the velocity response, periods of both rising and falling velocities, reflecting the complex interplay between in-situ stress and acoustic wave propagation.

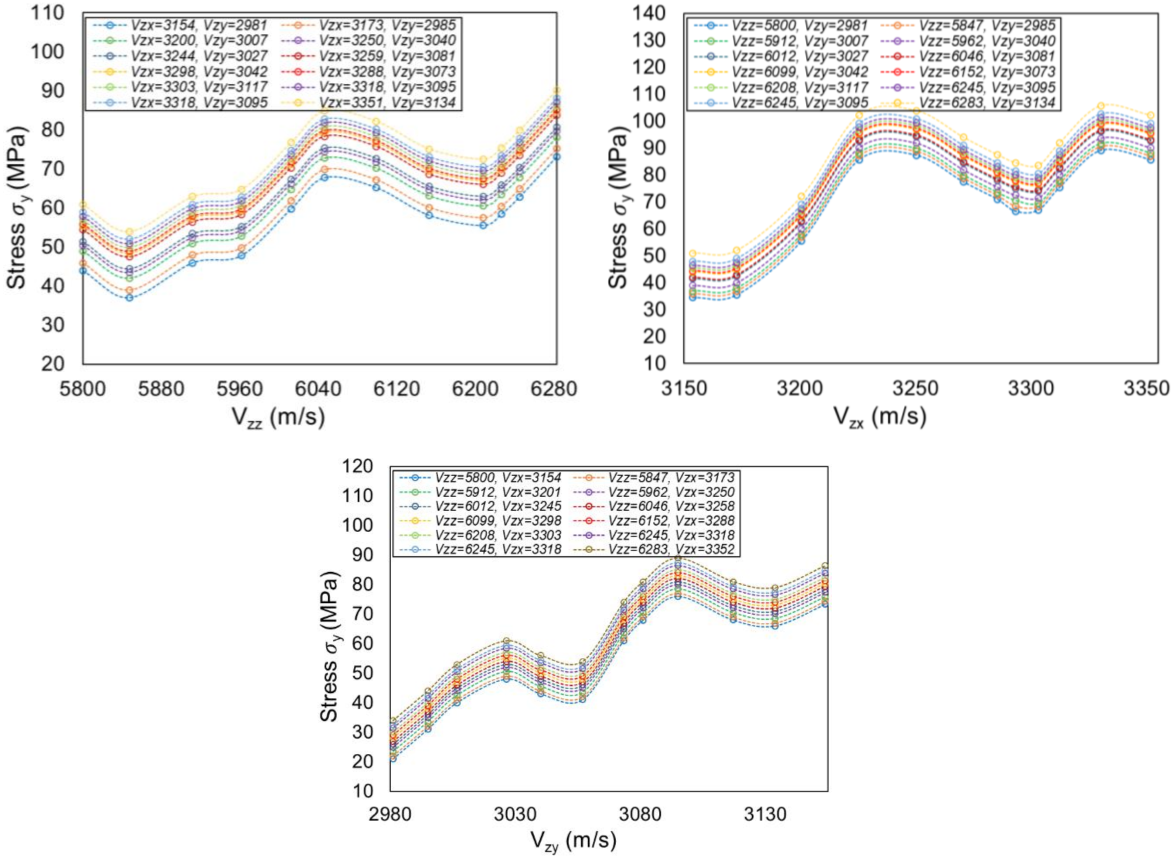

Figure S25: Parametric/Sensitivity study exhibiting influence of input features on predicted σ_y stress.

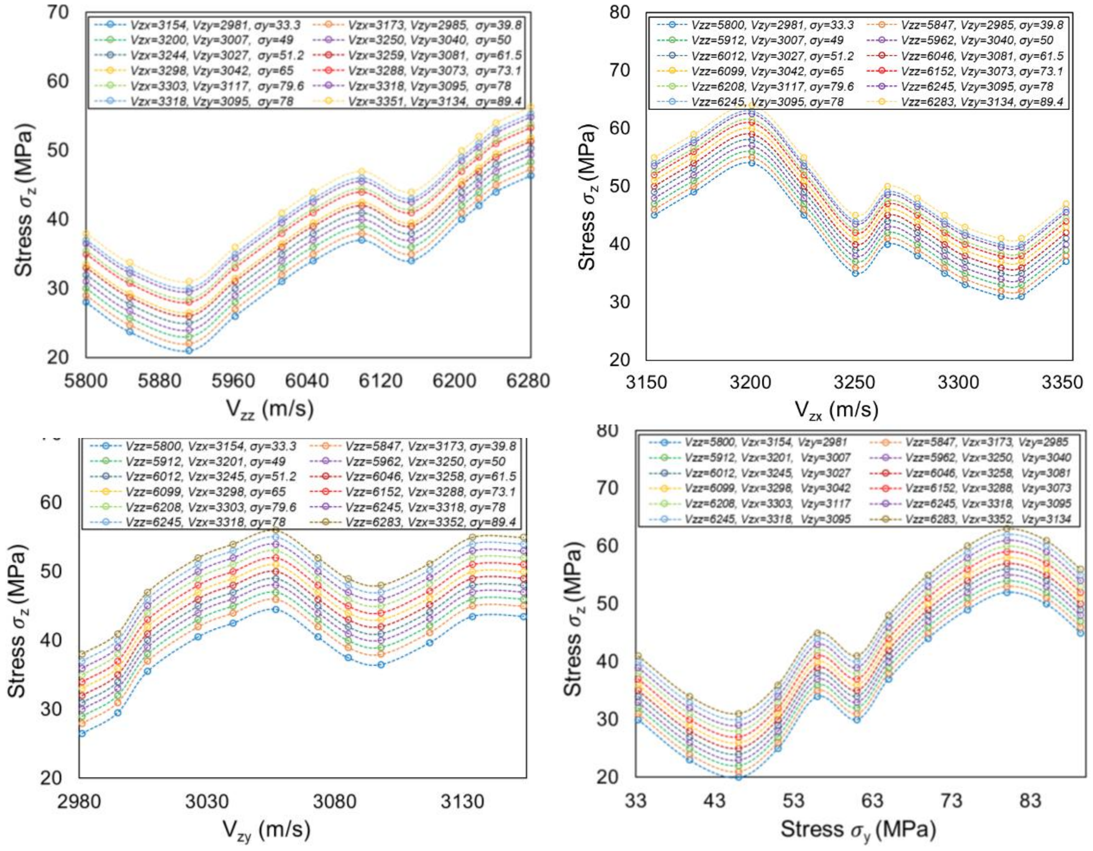

Figure S26: Parametric/Sensitivity study exhibiting influence of input features on predicted σ_z stress.

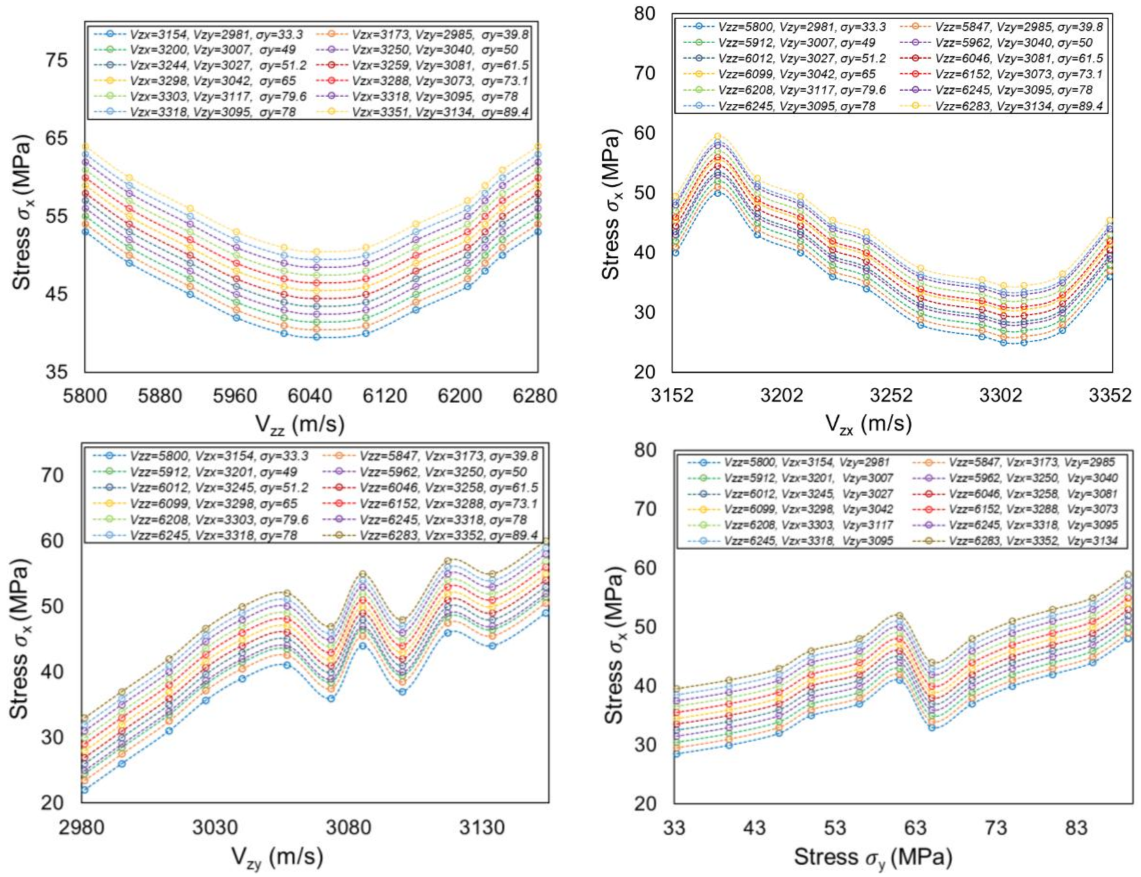

Figure S27: Parametric/Sensitivity study exhibiting influence of input features on predicted σ_x stress.

Appendix O: Analysis of SHAP results of DNN models for σ_x and σ_z stresses.

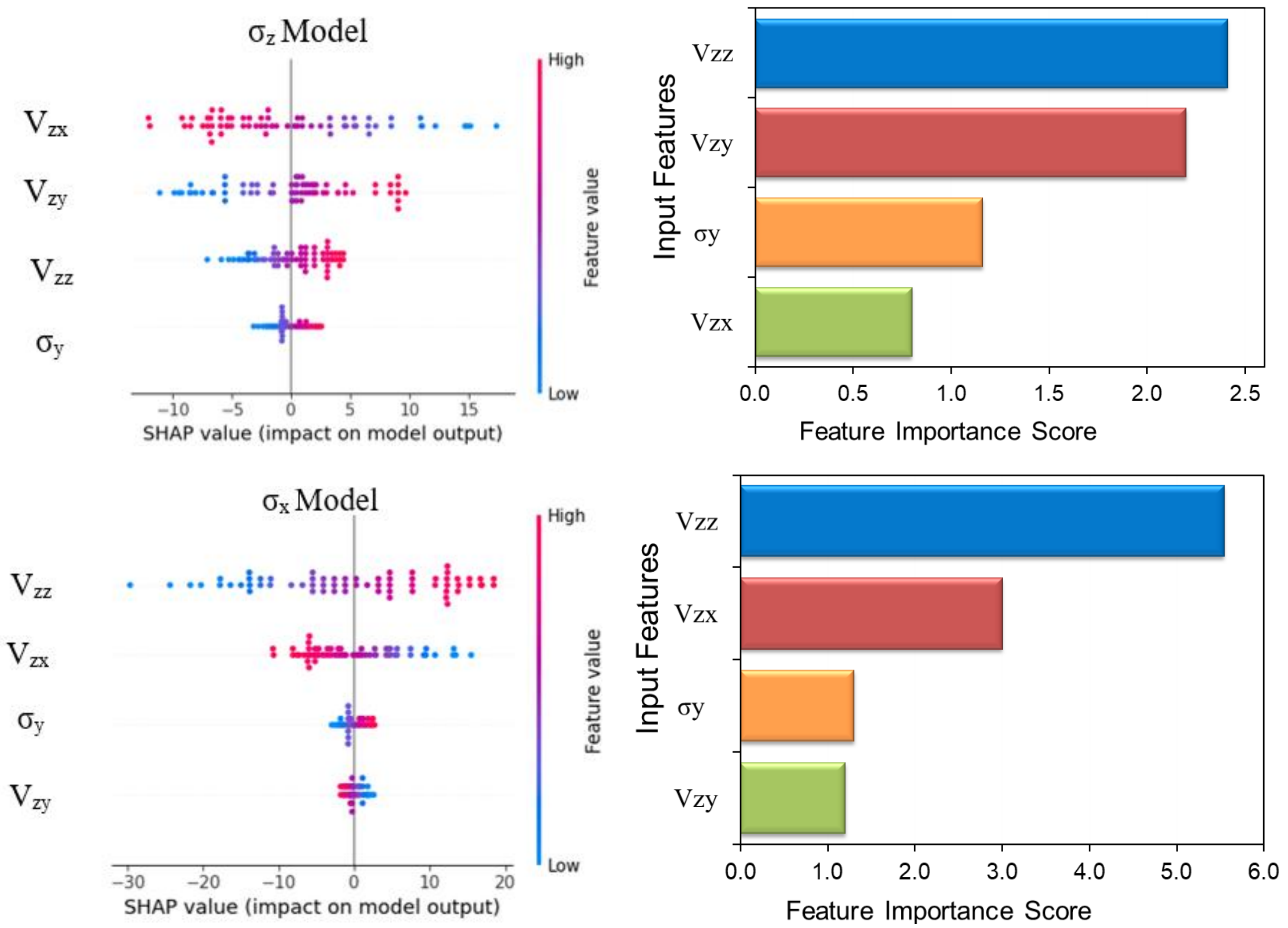

Figure S28: SHAP summary plots (left column) and corresponding feature importance scores (right column) of σ_z and σ_x stress models.

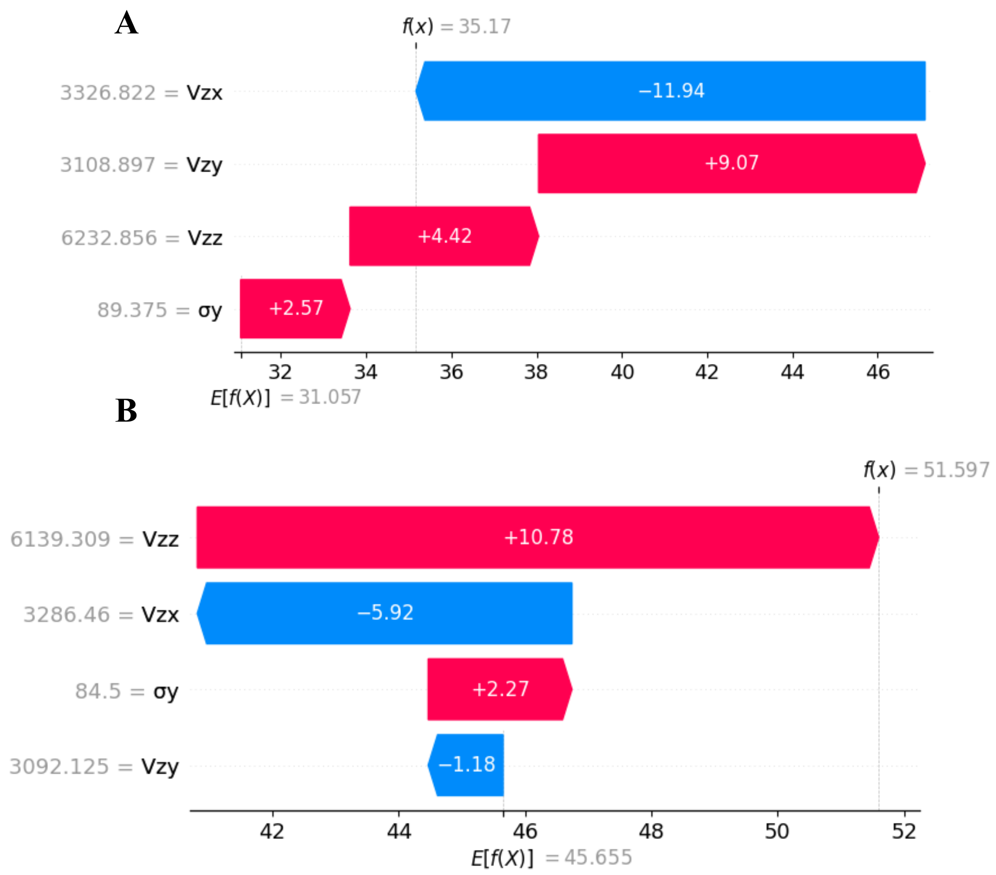

Figure S29: Waterfall plots of SHAP values for a particular data point reflecting effect of each feature on stress predictions; (A) σ_z, (B) σ_x.

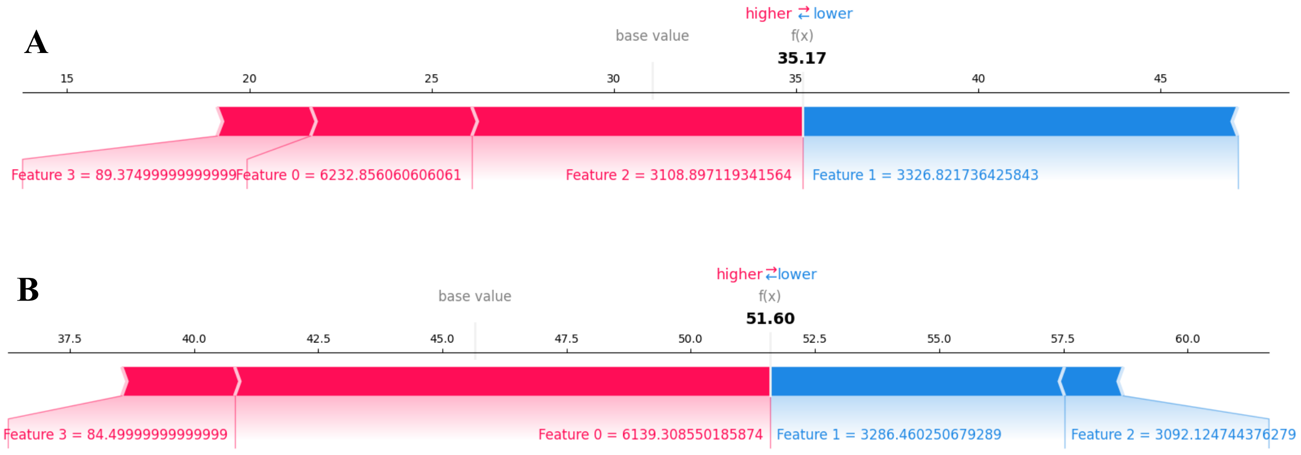

Figure S30: Force plots of SHAP values for a particular data point reflecting effect of each feature in on final stress predictions; (A) σ_z, and (B) σ_x.

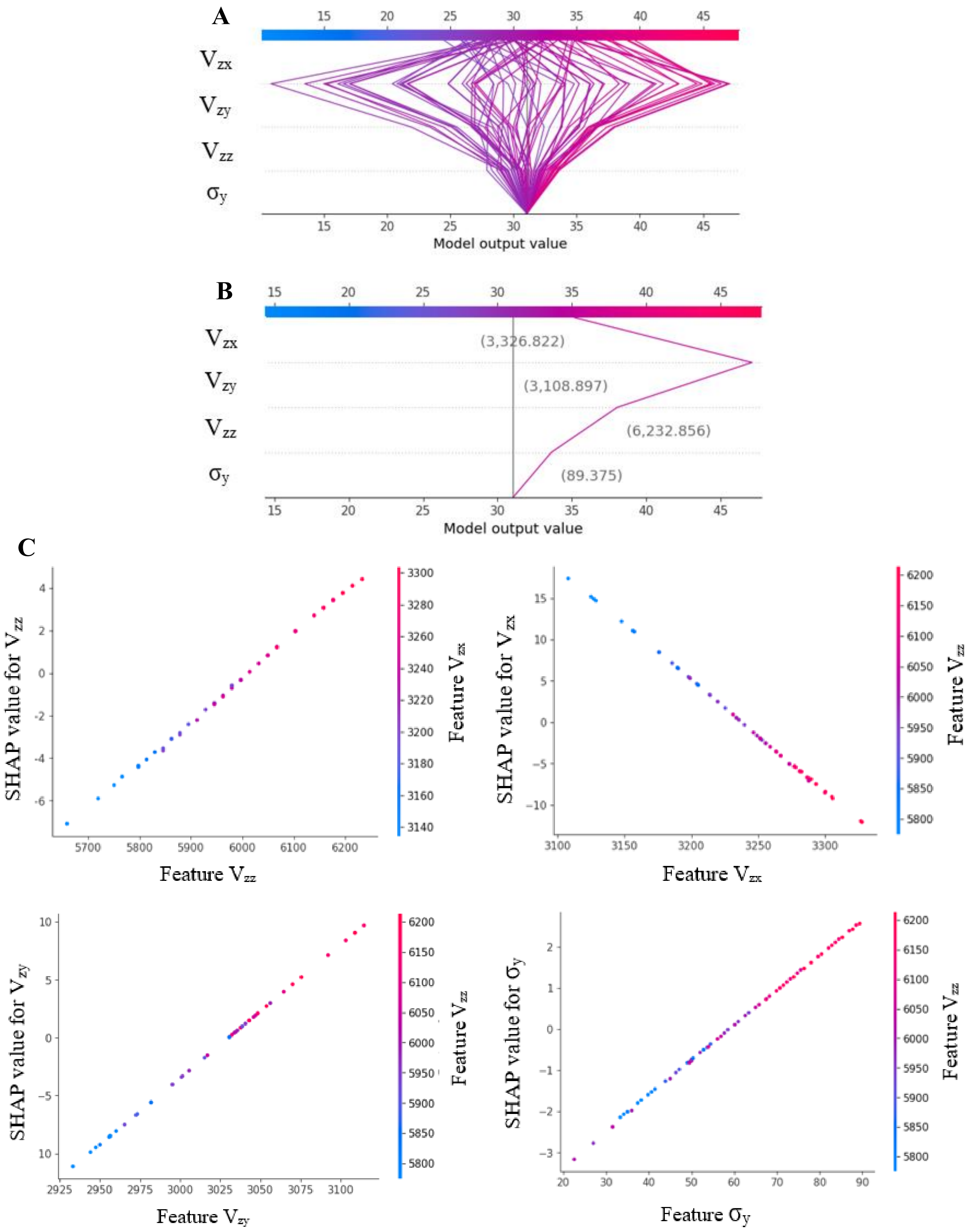

Figure S31: Decision and dependence plots of SHAP values for σ_z stress model; (A) Decision plot of a few data points or instances, (B) Decision plot of a single data point or instance, (C) Dependence plots of input features V_{zx}, V_{zy}, and V_{zz}.

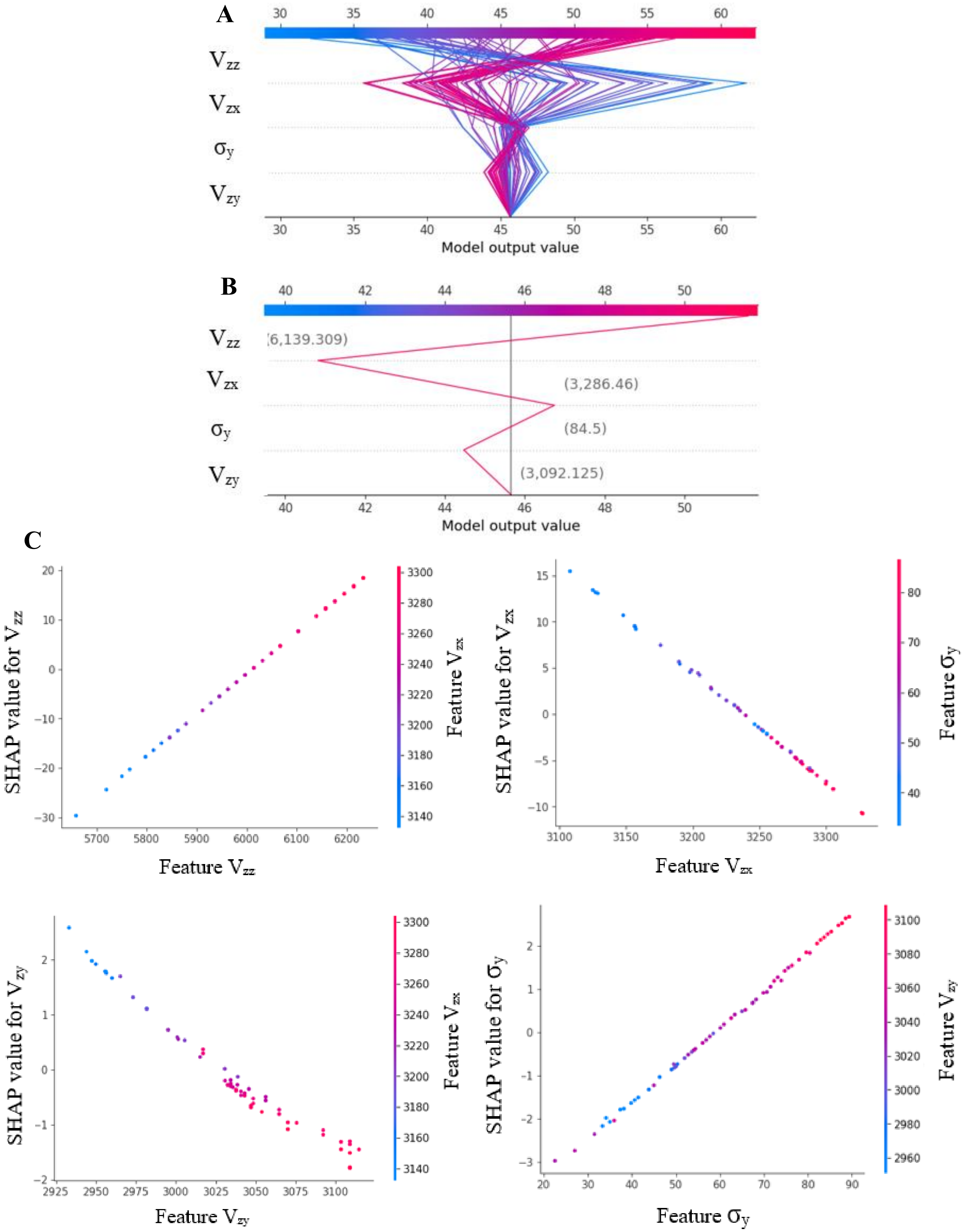

Figure S32: Decision and dependence plots of SHAP values for σ_x stress model; (A) Decision plot of a few data points or instances, (B) Decision plot of a single data point or instance, (C) Dependence plots of input features V_{zx}, V_{zy}, and V_{zz}.

Appendix P: Subsurface rock classification into Petrofacies using unsupervised ML algorithm.

In this phase, the unsupervised K-means clustering method was applied to categorize the subterranean rocks into distinct groups or clusters, adopting a similar methodology outlined by [37] and [38]. Here, the identified clusters are termed as Petrofacies. The well log data, including neutron porosity (NPHI), photoelectric factor (PEF), bulk density (ρ), and gamma ray (GR), were collected over the measured depth range from 4835 to 10872 ft of the same geothermal well 16B(78)-32. Further details regarding the identification and optimization process for clusters (petrofacies) are presented in Appendix P.

Six (06) petrofacies (PF) were optimally recognized in the given depth range and rock properties which illustrates spatial variability in subterranean rocks. The three core samples used in this work are illustrated by three specific PF namely PF-6, PF-5 and PF-1. Core-A, B, and C belong to PF-1 (sky blue), PF-6 (purple), and PF-5 (blue), respectively. The representative PFs are the illustration of petrophysical and formation properties of the subterranean rock amples, thus, demonstrating the constitutive elastic behavior of corresponding core samples. The suite of well logs and the corresponding PF classes the entire well section are shown in Figure S33.

The goal of classifying subsurface rock was to identify the representative petrofacies (PF) corresponding to the depths of the core samples analyzed in this study. Each cluster obtained through this process corresponded to a unique PF, encapsulating a specific combination of petrophysical and formation properties. The K-means algorithm was applied iteratively across varying numbers of clusters to determine the optimal PF count. In each iteration, the algorithm calculated the sum of squared distances from each data point to its nearest centroid commonly referred to as the inertia. These inertia values were then plotted in an elbow chart (Figure S34) to identify the cluster count at which adding more clusters yielded diminishing returns. The resulting PF and their relationships, illustrated via cross-plots of the selected rock properties, are also presented in Figure S34.

The clustering models' effectiveness was evidenced by an overall silhouette index of 0.70, signifying well-defined and cohesive clusters. In particular, the data points within PF-1, PF-4, PF-5, and PF-6 were

concentrated in narrow, separated clusters. This work, however, was mainly focused on PF 1, PF 5 and PF 6, as these clusters represented best the properties of the core samples. On the other hand, PF 2 and PF 3 demonstrated lower compactness and weaker edges, likely as a result of complexity of geology and transition facies that generate gradual changes in rock properties. The representative PF zones were then mapped both above and below the core sampling depths along the wellbore.

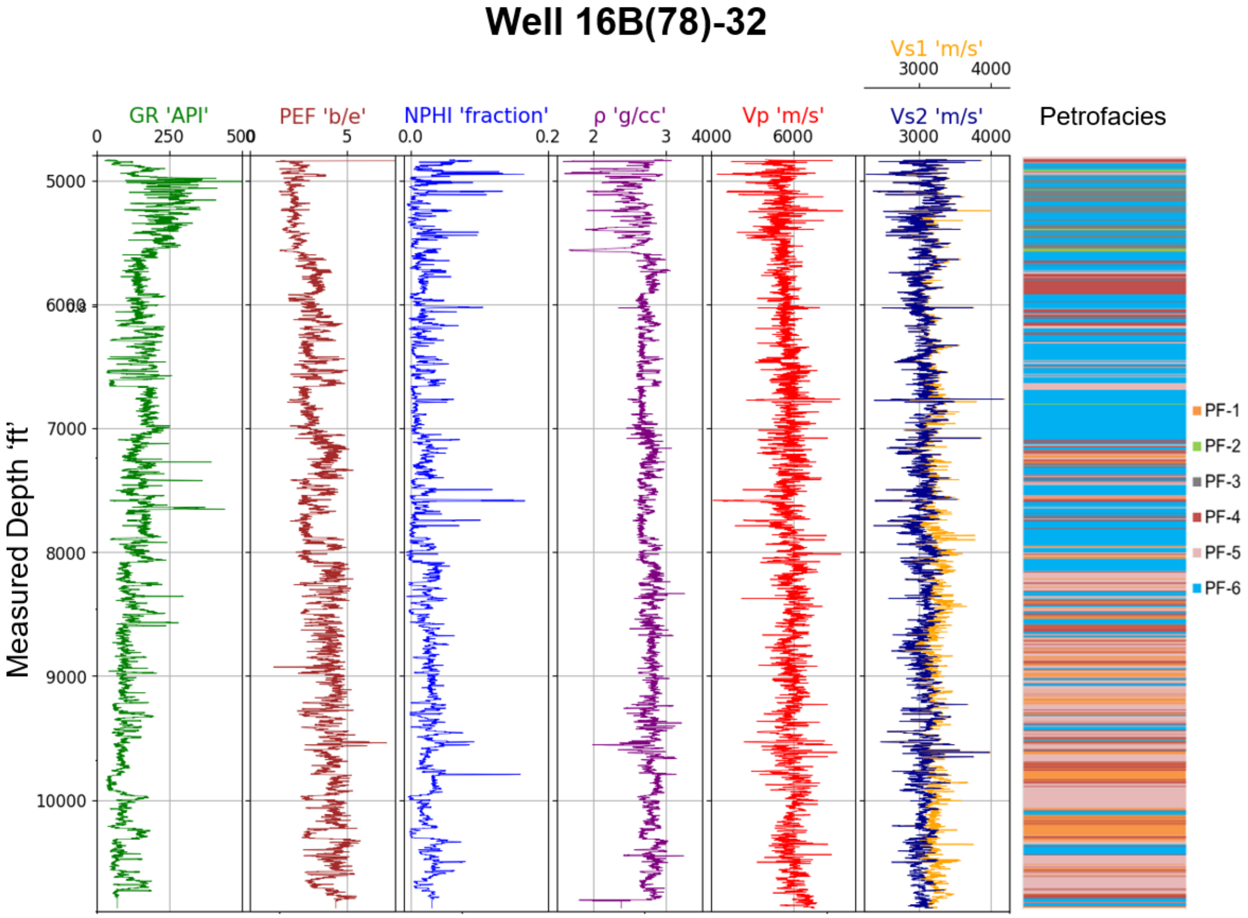

Figure S33: A suite of petrophysical well logs and identified clusters in well 16B(78)-32

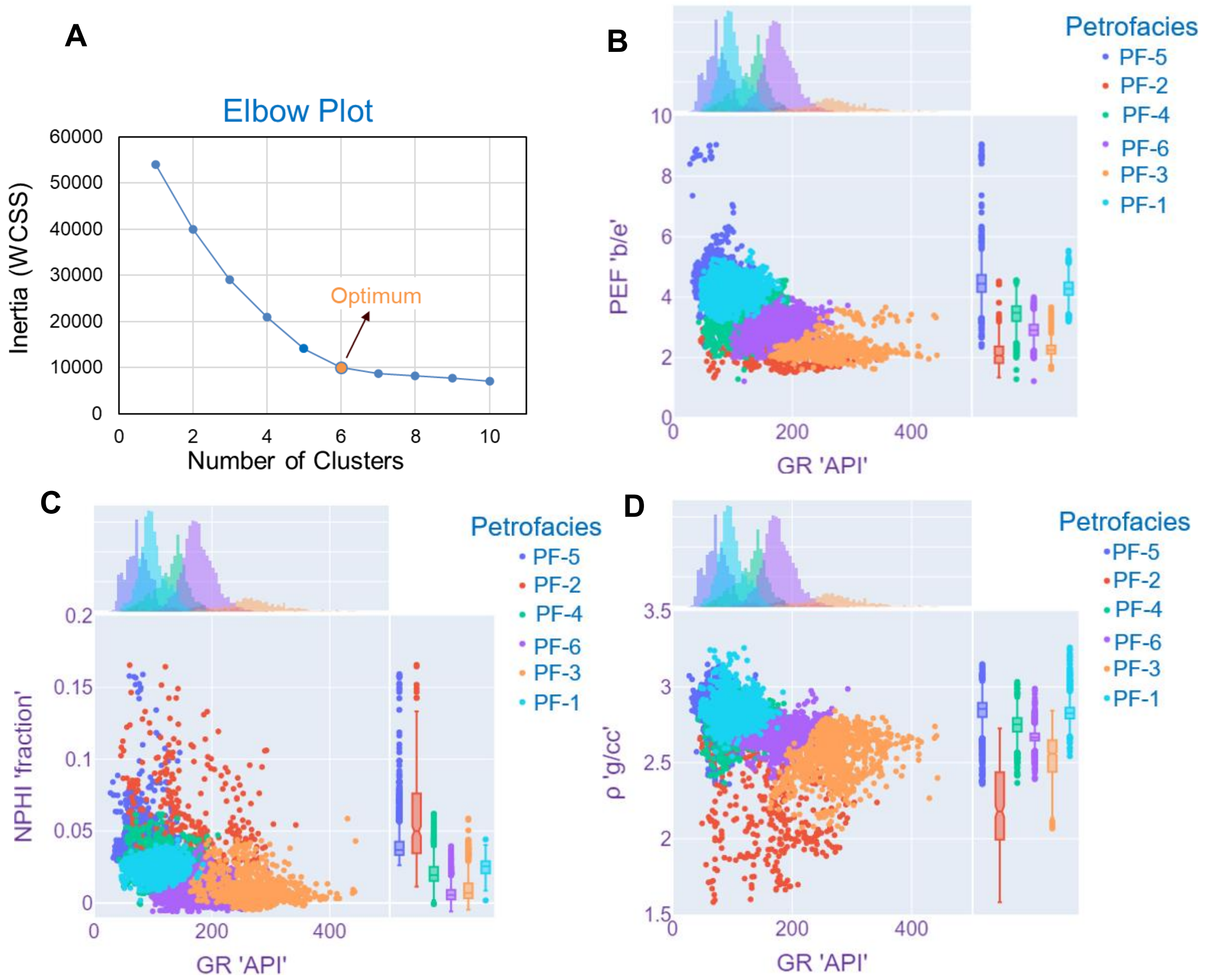

Figure S34: K-means clusters results; (A) Elbow plot, (B) GR vs PEF, (C) GR vs NPHI, (D) GR vs ρ for the depth interval 4835 - 10872 ft of well 16B(78)-32.

REFERENCES

[1] Mozaffari, A., & Azad. N. L. (2014). Optimally pruned extreme learning machine with ensemble of regularization techniques and negative correlation penalty applied to automotive engine coldstart hydrocarbon emission identification. Neurocomputing 131, 143-156.

[2] Mohaghegh, S., Arefi, R., Ameri, S., & Rose, D. (1995). Design and development of an artificial neural network for estimation of formation permeability. SPE Computers Applications 7 (06), 151-154. (SPE-28237-PA) https://doi.org/10.2118/28237-pa.

[3] Mustafa, A., Tariq, Z., Mahmoud, M., Radwan, A. E., Abdulraheem, A., & Abouelresh, M. O. (2022). Data-driven machine learning approach to predict mineralogy of organic-rich shales: An example from Qusaiba Shale, Rub'al Khali Basin, Saudi Arabia. Marine and Petroleum Geology, 137, 105495.

[4] Ali, J. K. (1994). Neural networks: a new tool for the petroleum industry? Proceedings, Society of Petroleum Engineers European Petroleum Computer Conference, SPE/EPCC, Aberdeen, UK, 15-17 Mar, 1994. Society of Petroleum Engineers, 233–242. (SPE-27561-MS). https://doi.org/10.2523/27561-ms

[5] Mustafa, A., Tariq, Z., Mahmoud, M., & Abdulraheem, A. (2023). Machine learning accelerated approach to infer nuclear magnetic resonance porosity for a middle eastern carbonate reservoir. Sci Rep 13, 3956 (2023).

[6] Otchere, D. A., Ganat, T. O. A., Gholami, R., & Ridha, S. (2021). Application of supervised machine learning paradigms in the prediction of petroleum reservoir properties: Comparative analysis of ANN and SVM models. Journal of Petroleum Science and Engineering 200, 108182.

[7] Mustafa, A., Tariq, Z., Abdulraheem, A., Mahmoud, M., Kalam, S., & Khan, R. A. (2022). Shale brittleness prediction using machine learning—A Middle East basin case study. AAPG Bulletin, 106(11), 2275-2296.

[8] Chau, K. W. (2007). Application of a PSO-based neural network in analysis of outcomes of construction claims. Automation in Construction 16 (5), 642-646.

[9] Saggaf, M., Toksoz, M. N., & Mustafa, H. M. (2003). Estimation of reservoir properties from seismic data by smooth neural networks. Geophysics 68, 1969–1983. https://doi. org/10.1190/1.1635051

[10] Rashid, S., Mustafa, A., Iqbal, A., Farooq, M. U., Butt, M. M., & Naeem, M. (2025). Data driven-based machine learning modelling and empirical correlations for predicting snow-covered area in the Swat Region, Pakistan. Next Sustainability, 5, 100074.

[11] Saikia, P., Baruah, R. D., Singh, S. K., & Chaudhuri, P. K. (2020). Artificial Neural Networks in the domain of reservoir characterization: A review from shallow to deep models. Computers and Geosciences 135, 104357.

[12] Mustafa, A., Tariq, Z., Yan, B., Han, Z., & Iqbal, A. (2024). Towards Rapid Prediction of Nuclear Magnetic Resonance-Based Bimodal Porosities: An Example from the Middle Eastern Carbonate Reservoir. Arabian Journal for Science and Engineering, 1-21.

[13] LeCun, Y., Bottou, L., Bengio, Y., & Haffner, P. (1998). Gradient-based learning applied to document recognition. *Proceedings of the IEEE*, 86(11), 2278-2324.

[14] Goodfellow, I., Bengio, Y., & Courville, A. (2016). Deep Learning. MIT Press.

[15] Zhang, Y., Chen, W., Wang, J., & Lin, X. (2017). Deep regression networks for predicting physical properties. IEEE Transactions on Neural Networks and Learning Systems, 28(8), 1715–1726. https://doi.org/10.1109/TNNLS.2016.2566058

[16] Hosseini, S. A., Weissend, M. A., Maier, A., & Christlein, V. (2021). Convolutional neural networks for regression: applications in magnetic resonance imaging. Applied Intelligence, 51, 8411–8429.

[17] Zhu, X., Li, Y., Xia, J., Yan, N., & Wang, Q. (2021). Geospatial Regression From Satellite Imagery With Convolutional Neural Networks. IEEE Journal of Selected Topics in Applied Earth Observations and Remote Sensing, 14, 4920–4933. https://doi.org/10.1109/JSTARS.2021.3076819

[18] Guo, Z., Moosavi, V., & Leitao, J. P. (2022). Data-driven rapid flood prediction mapping with catchment generalizability. Journal of Hydrology, 609, 127726.

[19] Cho, K., van Merriënboer, B., Gulcehre, C., Bahdanau, D., Bougares, F., Schwenk, H., & Bengio, Y. (2014). Learning phrase representations using RNN encoder-decoder for statistical machine translation. arXiv preprint arXiv:1406.1078.

[20] Chung, J., Gulcehre, C., Cho, K., & Bengio, Y. (2014). Empirical evaluation of gated recurrent neural networks on sequence modeling. arXiv preprint arXiv:1412.3555.

[21] Yin, Z., Kann, K., Yu, M., & Schütze, H. (2017). Comparative study of CNN and RNN for natural language processing. arXiv preprint arXiv:1702.01923.

[22] Zhou, Y., Xu, X., Zhang, C., Xiong, Y., & Lin, W. (2016). Beyond Short Snippets: Deep Networks for Video Captioning. In Proceedings of the IEEE Conference on Computer Vision and Pattern Recognition (CVPR), 4707–4715.

[23] Palmer, D. S., O'Boyle, N. M., Glen, R. C., & Mitchell, J. B. O. (2007). Random forest models to predict aqueous solubility. Journal of Chemical Information and Modeling 47(1):150 – 8. https://doi.org/10.1021/ci060164k

[24] Svetnik, V., Liaw, A., Tong, C., Culberson C. J., Sheridan, R. P., & Feuston, B. P. (2003). Random Forest: A Classification and Regression Tool for Compound Classification and QSAR Modeling. Journal of Chemical Information & Computer Sciences 43, 1947 – 58. https://doi.org/10.1021/ci034160g.

[25] Friedman, J., Hastie, T., & Tibshirani, R. (2000). Additive logistic regression: a statistical view of boosting. The Annals of Statistics 28(2), 337-407.

[26] Chen, T., He, T., Benesty, M., Khotilovich, V., Tang, Y., Cho, H., Chen, K., Mitchell, R., Cano, I. & Zhou, T. (2015). Xgboost: extreme gradient boosting. R package version 0.4-2, 1(4), 1-4.

[27] Wang, C., Deng, C., & Wang, S. (2020). Imbalance-XGBoost: leveraging weighted and focal losses for binary label-imbalanced classification with XGBoost. Pattern Recognition Letters 136, 190-197.

[28] Yang, X., Wang, J., & Zhang, I. (2017). Application of XGBoost in iltra-short load forecasting. Electric Drive Automation 39, 21-25.

[29] Zhao, T., Zheng S., Li, W., & Liu, K. (2018). Research on credit risk analysis based on XGBoost. Software Engineering 21 (6), 29-32.

[30] Steinhaus, H. (1956). Sur la division des corps matériels en parties. Bulletin de l'Académie Polonaise des Sciences, Classe III, vol. IV, no. 12, 801–804.

[31] MacQueen, J. (1967). Some methods for classification and analysis of multivariate observations, in: Fifth Berkeley Symposium on Mathematics. Statistics and Probability, University of California Press, Berkeley, CA, pp 281–297.

[32] Jancey, R. C. (1966). Multidimensional group analysis, Australian Journal of Botany 14, 127–130.

[33] Jain, A. K. (2010). Data clustering: 50 years beyond K-means. Pattern Recognition Letters 31(8), 651–666.

[34] Drineas, P., Frieze, A. M., Kannan, R., Vempala, S. S., & Vinay, V. (1999). Clustering in large graphs and matrices, Proceedings, ACM-SIAM Symposium on Discrete Algorithms, (SODA), 17-19 Jan 1999, 291–299.

[35] Ahmad, A., & Khan, S. S. (2019). Survey of state-of-the-art mixed data clustering algorithms, IEEE Access 7, 31883–31902.

[36] Bunger, A. P., Higgins, J., Huang, Y., & Kelley, M. (2023). Core-Based In-Situ Stress Estimation for Utah FORGE Well 16A(78)-32 using Triaxial Ultrasonic Velocity and Deformation Rate Analysis. Project Milestone Report 2.1.1 for Utah FORGE Project 2439. https://gdr.openei.org/submissions/1438

[37] Mustafa, A., Kelley, M., Lu, G., & Bunger, A. P. (2024). An Integrated Machine Learning Workflow to Estimate In-Situ Stresses Based on Downhole Sonic Logs and Laboratory Triaxial Ultrasonic Velocity Data. Journal of Geophysical Research: Machine Learning and Computation, 1(4), e2024JH000318. https://doi.org/10.1029/2024JH000318

[38] Mustafa, A., Lu, G., Bunger, A. P. (2026) Evolution of learning curve and white box machine learning models for estimating in-situ stresses based on velocity-stress relationship. Fuel, Vol. 404, Part B, 136306, https://doi.org/10.1016/j.fuel.2025.136306
